# Exploring Hydrophilic PD-L1 Radiotracers Utilizing Phosphonic Acids: Insights into Unforeseen Pharmacokinetics

**DOI:** 10.3390/ijms242015088

**Published:** 2023-10-11

**Authors:** Fabian Krutzek, Cornelius K. Donat, Sven Stadlbauer

**Affiliations:** 1 Helmholtz Zentrum Dresden-Rossendorf, Institute of Radiopharmaceutical Cancer Research, Department of Medicinal Radiochemistry, Bautzner Landstraße 400, 01328 Dresden, Germany; f.krutzek@hzdr.de (F.K.); c.donat@hzdr.de (C.K.D.); 2 School of Science, Faculty of Chemistry and Food Chemistry, Technical University Dresden, 01069 Dresden, Germany

**Keywords:** PD-L1, organic synthesis, structure-activity relationship, radiotracers, PET imaging, phosphonic acids

## Abstract

Immune checkpoint inhibitor therapy targeting the PD-1/PD-L1 axis in cancer patients, is a promising oncological treatment. However, the number of non-responders remains high, causing a burden for the patient and the healthcare system. Consequently, a diagnostic tool to predict treatment outcomes would help with patient stratification. Molecular imaging provides said diagnostic tool by offering a whole-body quantitative assessment of PD-L1 expression, hence supporting therapy decisions. Four PD-L1 radioligand candidates containing a linker-chelator system for radiometalation, along with three hydrophilizing units—one sulfonic and two phosphonic acids—were synthesized. After labeling with ^64^Cu, log *D*_7.4_ values of less than −3.03 were determined and proteolytic stability confirmed over 94% intact compound after 48 h. Binding affinity was determined using two different assays, revealing high affinities up to 13 nM. µPET/CT imaging was performed in tumor-bearing mice to investigate PD-L1-specific tumor uptake and the pharmacokinetic profile of radioligands. These results yielded an unexpected in vivo distribution, such as low tumor uptake in PD-L1 positive tumors, high liver uptake, and accumulation in bone/bone marrow and potentially synovial spaces. These effects are likely caused by Ca^2+^-affinity and/or binding to macrophages. Despite phosphonic acids providing high water solubility, their incorporation must be carefully considered to avoid compromising the pharmacokinetic behavior of radioligands.

## 1. Introduction

The programmed cell death protein PD-1 (CD279) and its ligand PD-L1 (CD274) form an immune checkpoint, regulating the immune response within the tumor microenvironment [1,2]. Under homeostatic conditions, these checkpoints limit autoimmune or immune-related adverse effects following an inflammatory response. However, malignant tumors often overexpress the corresponding targets, such as PD-L1, allowing them to evade the local immune response within and around the tumor and facilitate proliferation. Inhibitors of this pathway (checkpoint inhibitors) can reactivate the local immune response by blocking PD-1 or PD-L1, allowing immune cells to recognize and attack tumor cells. Hence, the PD-1/PD-L1 axis is an attractive pathway for the therapy of solid cancers. Unfortunately, only an average of 30% of patients respond to antibody-based checkpoint inhibitor monotherapies. This complicates the decision process for the physician and the patient and is an economic dilemma, as antibody therapy is expensive [3,4,5] and can cause adverse effects [6]. Currently, patient stratification and therapy decisions rely on immunohistochemical methods after biopsies, which do not account for a potentially heterogeneous expression of PD-L1 among and within tumor lesions. Additionally, these biopsies—which might be required multiple times—are painful and can cause adverse effects [7,8]. Therefore, diagnostic tools to monitor and support therapy decisions are needed to improve immune checkpoint inhibitor therapies [9]. Non-invasive molecular imaging techniques, such as positron emission tomography (PET) and single-photon emission computed tomography (SPECT), could precisely and quantitatively measure PD-L1 expression over time, lowering the burden for the patient drastically [10]. Various types of radiotracer classes targeting PD-L1 have been developed [9], such as antibodies [11,12,13,14], nanobodies [15,16,17], affibodies [18,19,20], and adnectines [21,22]. Some of these radiotracers [19,21,22] are undergoing clinical trials and show promise as diagnostic tools for immune checkpoint inhibitor therapy [11,15,23,24].

However, applying high molecular weight molecules for in vivo molecular imaging, specifically full-size antibodies, has some drawbacks. The compounds can exhibit prolonged circulation times, low vascular permeability and diffusivity [25]. This necessitates the use of long-living radioisotopes, which in turn increase the radiation burden of the patient. While therapeutic doses of PD-L1 antibodies, specifically atezolizumab, can cause immunogenicity effects (primarily anti-drug antibodies) in ~30% of patients [6], it is less likely to affect imaging due to the low amount injected. Primarily, the cost of antibody-based approaches is problematic, as manufacturing and therefore patient application can be substantial. Furthermore, structural modifications are more challenging to achieve in antibodies when compared to peptides or small molecules [26].

Peptides and small molecules lack some of these issues, making them more promising imaging agents. Specifically, the higher tissue and tumor penetration and short clearance times ideally result in high imaging contrast within minutes and hours. The scalability in production eases manufacturing and commercialization. Over the past few years, several peptide-based PD-L1 radiotracers have been developed and tested in preclinical studies. The most promising example is the cyclic peptide WL12 [27,28,29,30,31] which also already underwent clinical studies [32]. On the other hand, only three radiotracers [33,34,35] based on small molecules were reported for PD-L1, which failed to produce satisfying results. However, the plethora of reported PD-L1 inhibitors provides the basis for the development of small-molecule PD-L1 radiotracers [36,37,38,39,40,41].

In this study, we present the design, synthesis, radiochemistry, and biological evaluation of four new ^64^Cu-labeled radiotracers targeting PD-L1. These radioligands are derived from biphenyl-containing small-molecule inhibitors, which have been extensively investigated in recent years [42,43,44]. To ensure efficient and rapid synthesis, we employed a convergent synthetic route with modular building blocks. Given the reported high lipophilicity of those PD-L1 inhibitors, we attached hydrophilic units to the core structure, aiming at increasing water solubility and renal rather than hepatobiliary clearance [45,46,47]. To this end, we incorporated sulfonic acids in our previous studies [48], which, however, exhibited high albumin binding capacity. To overcome this, we utilized phosphonic acids in the present study, which are not known to exhibit albumin binding. The radiotracers were conjugated with the NODA-GA chelator to allow for labeling with copper-64, a radionuclide with favorable properties for PET imaging. Binding affinities were determined using both saturation and real-time binding. PET/CT studies were performed to investigate the pharmacokinetic profile and tumor accumulation of all ^64^Cu-labeled radioligands.

## 2. Results

### 2.1. Design and Synthethic Strategy

**Figure 1:**
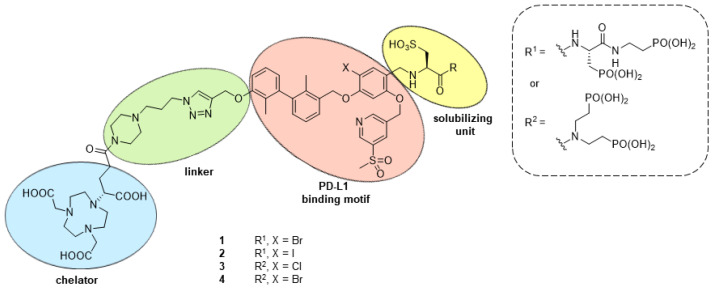
Schematic illustration of phosphonic acid containing PD-L1 radiotracers reported in this study.

The structure of the current PD-L1 radiotracers is based on the biphenyl PD-L1 inhibitor BMS-1166 [49,50] and our previously reported radioligands [48]. Next to the binding biphenyl, the tetrasubstituted chloroaryl and the pyridine moiety contribute with various interactions to the binding to PD-L1 (Figure 1, red). A larger bromine instead of a chlorine atom was found to positively affect affinity [51]. We experimentally tested chlorine, bromine and iodine at this position [48]. Despite lower affinity, we observed a positive influence on tumor uptake, which we attributed to higher B_max_ values for the bromo- and iodo-substituted radioligands. Hence, we again varied the halogen at the central aromatic ring, hoping for a positive influence on the in vivo performance. The linker was attached to the biphenyl via copper(I)-mediated azide-alkyne cycloaddition (Figure 1, green) with its conjugated NODA-GA chelator (Figure 1, blue). The pyrazine ring and NODA-GA chelator already improve the water solubility of the radioligand, however, the incorporation of two sulfonic acids still resulted in hepatobiliary clearance [48]. Therefore, one additional hydrophilizing unit in the solvent-exposed region of the PD-L1 dimer (Figure 1, yellow) was introduced, assuming a negligible influence on affinity. Being aware of bone-seeking properties of bisphosphonates [52,53], we tested different substitution patterns with a substantial spatial distance of both moieties, hoping to avoid calcium affinity.

### 2.2. Retrosynthetical Analysis and Forward Synthesis

**Scheme 1:**
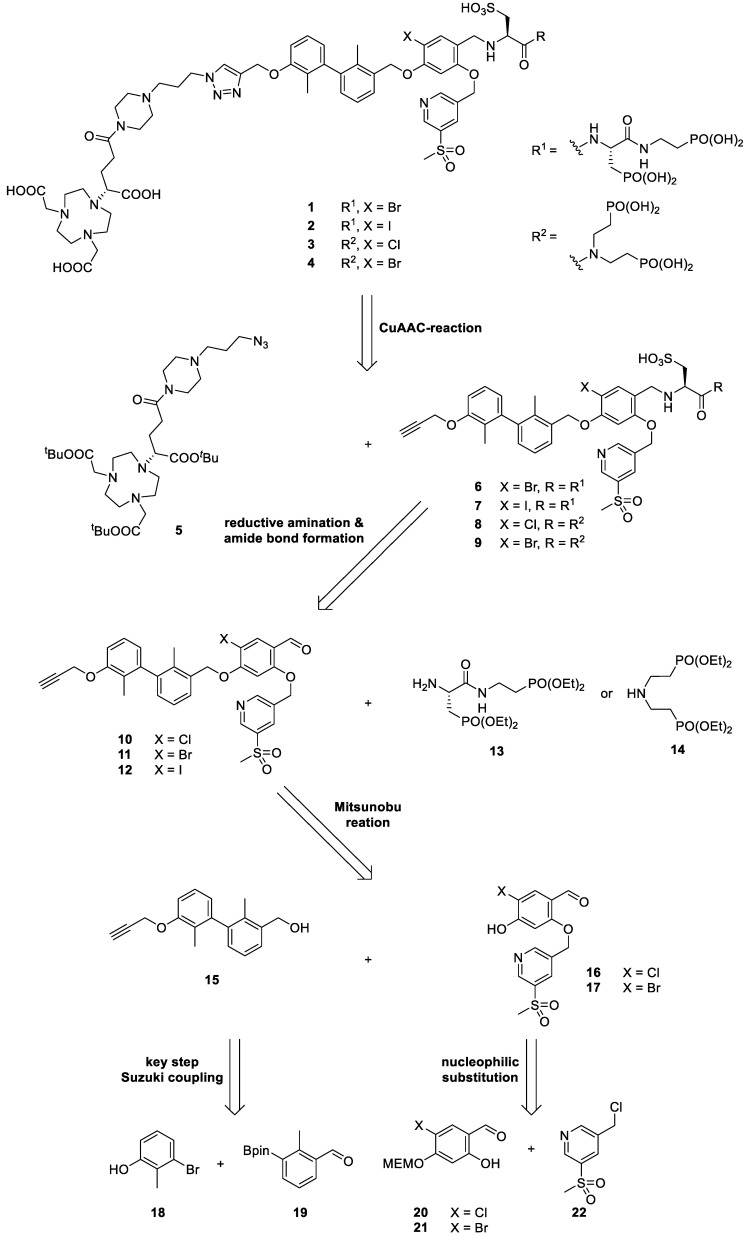
Retrosynthetic analysis of the herein presented PD-L1 ligands.

The synthetic route followed a convergent strategy to achieve a higher yield. In addition, the modular approach enables rapid access to a library of compounds with limited effort and synthetic demand. The final compounds **1**–**4** were obtained by global deprotection of carboxylic and phosphonic acids after CuAAC-reaction of the linker-chelator system **5** with the alkynes **6**–**9**. These alkynes with two phosphonic and one sulfonic acid were synthesized from the corresponding aldehydes **10**–**12** via reductive amination with l-cysteic acid and amide bond formation of amines **13** and **14**, respectively. Whereas amine **14** is known [54], **13** was synthesized from Fmoc and *^t^*Bu protected l-serine in five steps. The aldehydes **10**–**12** were partly reported in our previous study [48]: They are obtained by a key Mitsunobu-reaction of the biaryl **15** with phenols **16** and **17** (for the iodine derivative the route had to be slightly adjusted). Biaryl **15** was obtained via a key Suzuki coupling between commercially available or literature known aromatic building blocks **18** and **19** [55]. The central aromatic ring, substituted with chlorine (**16**) or bromine (**17**) was synthesized from aromatic building blocks **20** and **21** via nucleophilic substitution with sulfonyl pyridine **22** (Scheme 1).

**Scheme 2:**
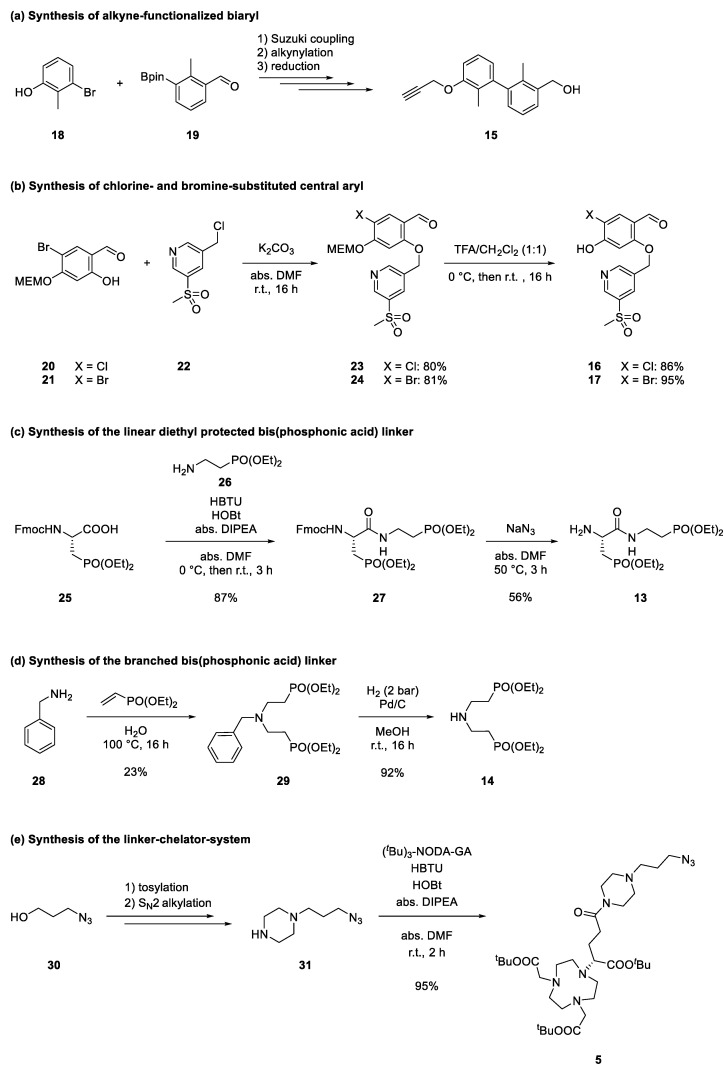
Synthesis of the building blocks required for the convergent synthesis of PD-L1 ligands.

**Scheme 3:**
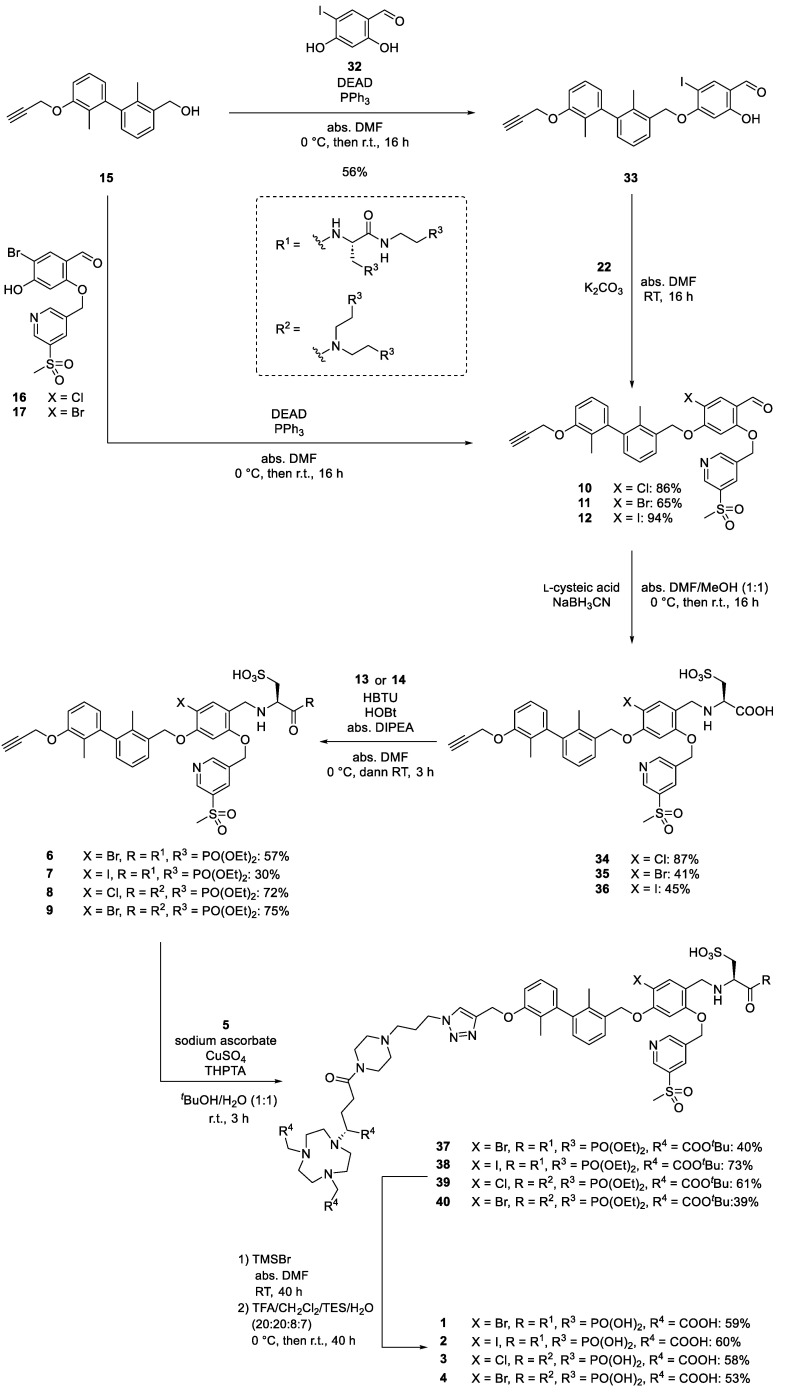
Final reaction steps toward four highly water soluble, phosphonic acid containing PD-L1 ligands **1**–**4**.

The forward synthesis can be divided into the separate syntheses of the five main building blocks (see Scheme 2): Alkyne-functionalized biaryl **15**, central aryl core with either chlorine (**16**) or bromine (**17**) (the synthesis of the iodinated compound followed a linear approach according to Scheme 3), the diethyl protected bis(phosphonate)-structures **12** and **13** and the linker-chelator-system **5**.

We reported the synthesis of the biaryl **15** previously in detail [48]. Briefly, the building blocks **18** and **19** underwent Suzuki coupling followed by nucleophilic substitution with propargyl bromide. Reduction in the aldehyde moiety provided benzylic alcohol **15** [48]. The MEM-protected chlorine (**20**) [48] and bromine (**21**) [48] bearing phenols reacted with benzylic chloride **22** through nucleophilic substitution in excellent yields of 84 and 81%. Cleavage of the MEM-group was achieved with trifluoroacetic acid (TFA) in dichloromethane (DCM), providing the phenols **16** and **17** in excellent yields. In the case of the linear ethyl protected bis (phosphonic acid) **13**, the synthesis started from carboxylic acid **25** (see Supplementary Information, synthetic procedures). Coupling with **26** [56] proceeded in an excellent yield of 87%. Cleavage of the Fmoc-group failed under conventional methods (piperazine, piperazine and 1,8-Diazabicyclo[5.4.0]undec-7-ene (DBU) or morpholine in *N*,*N*-dimethylformamide (DMF)) [57], leading to various side products. Similarly, tetra-*n*-butylammonium fluoride (TBAF) [58] also resulted in a mixture of undesired side products. However, the deprotection with sodium azide in abs. DMF [59] provided the desired amine **12** in an acceptable yield of 56%. The synthesis of the secondary amine **14** bearing two ethyl-protected ciliatine moieties followed the literature [54], slightly modified by using bottled hydrogen gas at room temperature instead of in situ generated hydrogen gas from ammonium formiate for removing the benzyl-protecting group. The linker-chelator-system **5** was synthesized from **31** (two steps from alcohol **30 [60]**) with commercially available (^t^Bu)_3_-NODA-GA and subsequent purification with preparative high-performance liquid chromatography (HPLC, detection wavelength 220 nm) (Scheme 2).

Access to carboxylic acid derivative **35** followed our previously reported synthesis for **34**. In contrast to the chloride and bromide derivatives, the iodide derivative **36** was synthesized in a linear fashion due to low yields utilizing the convergent route. The biaryl **15** reacted with 2,4-dihydroxy-5-iodobenzaldehyde **32 [61]** in a Mitsunobu reaction with the more reactive phenol group to compound **33** in a yield of 56%. This was followed by nucleophilic substitution in an excellent yield of 94%, providing aldehyde **12**. Biaryl **15** reacted with bromoaryl **17** in a Mitsunobu reaction with a 65% yield to **11**. The following reductive amination with l-cysteic acid was performed in a mixture of abs. DMF/MeOH (1:1) to ensure the solubility of all reagents, yielding the carboxylic acids **34**–**36** after HPLC purification (41–87% yield). The amide bond with **13** or **14** was formed by a reaction in abs. DMF (HBTU, HOBt, abs. DIPEA) in yields between 30 and 75%. The CuAAC reaction with piperazine-NODGA-GA building block **5** proved to be difficult. Under aqueous conditions (water/*^t^*BuOH, 1:1) with sodium ascorbate, CuSO_4_ and THPTA as a ligand, none of the four derivatives yielded a complete conversion (r.t., up to 2 d) as monitored by analytical RP-HPLC. An increase of the temperature to 50 °C led to a vast amount of side products which were partly identified by mass spectrometry: Partial *^t^*Bu-deprotection and/or coordination of Cu^2+^ were observed, probably caused by the Lewis-acidity of the Cu^2+^-ion. A similar behavior was detected when the amount of the catalytic mixture was increased. The use of a Cu(I)-catalyst under exclusion of oxygen ([Cu(MeCN)_4_]PF_6_ (0.1 eq.), THPTA (0.1 eq.), degassed DMF) led to a complex mixture at room temperature after several hours. After three hours under aqueous conditions, maximum conversion (max. 80%) was reached and the reaction was purified by semi-preparative RP-HPLC, yielding the compounds **37**–**40** (39–73% yield). The global deprotection was performed in a two-step sequence with one HPLC-purification: The free phosphonic acids were obtained by a McKenna reaction with TMSBr in DMF (monitored by ^31^P-NMR) [62,63] and after quenching with MeOH and solvent removal, the *^t^*Bu-groups were removed with a mixture of TFA/CH_2_Cl_2_/TES/H_2_O (20:20:8:7) under stirring for up to two days. After this two-step procedure, the final compounds **1**–**4** were obtained in yields between 53–60% after RP-HPLC-purification (Scheme 3). The overall yields along the longest linear sequence for **1**–**4** were 5.9, 1.1, 1.0 and 1.3%, respectively.

### 2.3. Radiochemistry

The compounds **1**–**4** were incubated in 1 M aqueous HEPES solution (pH 4) with [^64^Cu]Cu^2+^ at a temperature of 50 °C for 10 min. Quantitative radiolabeling with molar activities up to 70 GBq·µmol^−1^ was confirmed by either radio-TLC or -HPLC. 

With compounds [^64^Cu]Cu-**1**–**4,** distribution coefficients (log *D*_7.4_) were determined using the shake flask method between *n*-octanol and PBS. The obtained values are shown in Table 1, confirming high hydrophilicity. The introduction of three hydrophilizing units and the NODA-GA chelator led to log *D*_7.4_ values up to −4.00 for the bromo-substituted radioligand [^64^Cu]Cu-**1**. The more hydrophobic iodine atom leads to the highest log *D*_7.4_ value in this series (−3.03 for [^64^Cu]Cu-**2**). Since no change in hydrophilizing units, binding motif and chelator was performed, the log *D*_7.4_ values for the other two radioligands [^64^Cu]Cu-**3** and [^64^Cu]Cu-**4** are in a similar range (−3.80 and −3.81, respectively).

**Figure 2:**
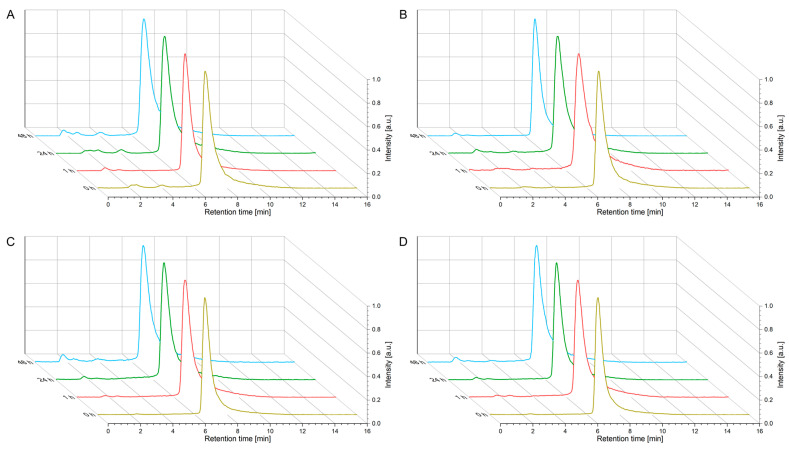
Radio-HPLC chromatograms of ^64^Cu-labeled compounds (**A**) [^64^Cu]Cu-**1**; (**B**) [^64^Cu]Cu-**2**; (**C**) [^64^Cu]Cu-**3**; (**D**) [^64^Cu]Cu-**4** after incubation in human serum at 37 °C and subsequent protein precipitation for 0 h (yellow), 1 h (red), 24 h (green) and 48 h (blue).

Subsequently, proteolytic stability was investigated (Figure 2). Radioligands [^64^Cu]Cu-**1**–**4** were incubated in human serum at 37 °C for up to 48 h. Before incubation (0 h) and 1, 24 and 48 h after incubation, an aliquot was taken, and serum proteins were precipitated. Analysis of radiotracers was performed with radio-HPLC, showing the intact radiotracer (0 h) at 6.6–6.7 min. At all time points, radiotracers remained intact up to 95%.

### 2.4. In Vitro Evaluation

The binding affinities of the ^64^Cu-labeled PD-L1 radiotracers [^64^Cu]Cu-**1**–**4** were determined using two different methods.

**Figure 3:**
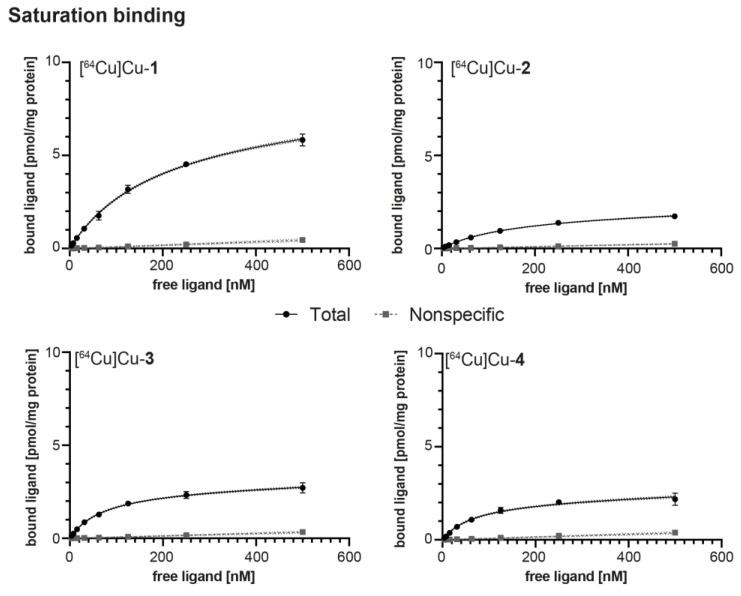
Saturation binding curves of [^64^Cu]Cu-**1**–**4**, with dotted lines representing 95% confidence intervals.

Firstly, saturation binding was performed on PC3 cells, stably overexpressing PD-L1, similar to described before [48] using eight concentrations of the corresponding radioligand (3.91–500 nM). Potential nonspecific binding to plastic was avoided via 2.5% bovine serum albumin (BSA) in the incubation buffer (PBS). Binding affinity (K_D_) and maximum number of binding sites (B_max_) were assessed and are shown in Table 1 and Figure 3, along with log *D*_7.4_ values and molar activities (A_M_).

K_D_ was found to vary depending on the substitution pattern of the phosphonic acids. Radioligands [^64^Cu]Cu-**1** and [^64^Cu]-Cu**2** with linear substitution of phosphonic acids show low binding affinities (190.7 and 154.1 nM, respectively). Affinity of [^64^Cu]Cu-**3** and [^64^Cu]Cu-**4** with branched substitution patterns are more promising, with values of 76.7 and 69.6 nM, respectively. The maximum number of binding sites is in a similar range (1.97 to 2.81 pmol·mg^−1^ protein) for compounds ([^64^Cu]Cu-**2**–**4**) and slightly higher for [^64^Cu]Cu-**1**.

To further validate the binding affinities of our radioligands to PD-L1, we employed real-time radioligand binding (LigandTracer^®^, Ridgeview Instruments AB, Sweden). 

As observed in our previous study [48], the binding affinities were influenced by the addition of BSA. Therefore, we performed this assay both in the presence (2.5%; *n* = 1) and absence of BSA (*n* = 2). This served the purpose of investigating kinetics at a level of BSA similar to the physiological conditions of albumin in the adult mouse blood (2.5 g/dL) (2.5 g/dL) [64].

**Figure 4:**
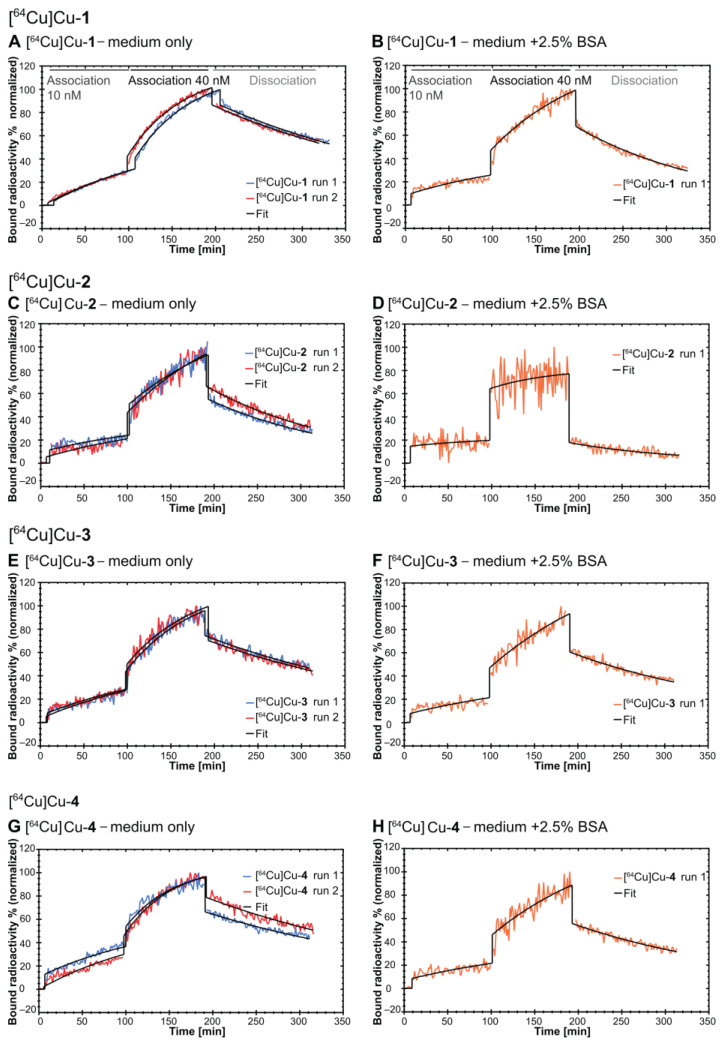
Real-time radioligand binding (trace) of compounds [^64^Cu]Cu-**1**–**4** the absence (**A**,**C**,**E**,**G**) and presence (**B**,**D**,**F**,**H**) of 2.5% bovine serum albumin (BSA).

The binding affinities (K_D_), kinetic parameters (association rate constant k_a_ and dissociation rate constant k_d_) and the normalized maximum number of binding sites are shown in Table 2, with Figure 4 providing the individual traces.

Experiments without BSA in the medium reflect the binding without serum protein interaction and reveal affinities in the low, two-digit nanomolar concentration range (K_D_ of 13.5, 17.4, 12.7 nM for [^64^Cu]Cu-**1**, [^64^Cu]Cu-**3** and [^64^Cu]Cu-**4**, respectively). The iodo-substituted compound [^64^Cu]Cu-**2** shows a dramatically decreased binding affinity of 270 nM.

In the presence of BSA, [^64^Cu]Cu-**1**, [^64^Cu]Cu-**3** and [^64^Cu]Cu-**4**, showed a decrease in binding affinity, except for [^64^Cu]Cu-**2** being in a similar range. These values are comparable to those determined in the saturation binding, using an identical albumin concentration.

### 2.5. In Vivo Evaluation

**Figure 5:**
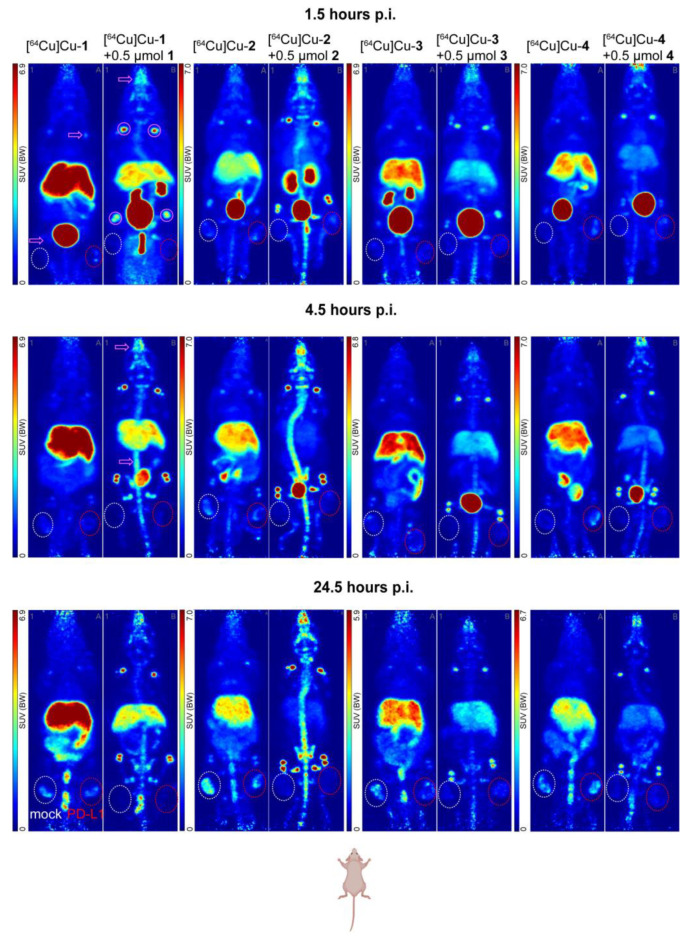
PET in vivo distribution (maximum intensity projections) of ^64^Cu-labeled compounds [^64^Cu]**1**–**4** (left to right) at 1.5, 4.5 and 24.5 h post-injection (p.i.) with 500 nmol of an unlabeled compound as a block. Red dashed circles indicate PC3 PD-L1 overexpressing tumors, white dashed circles indicate PC3 tumor transduced with a mock construct, hence lacking PD-L1 expression. Bone and joint uptake is exemplified for [^64^Cu]Cu-**1**, with solid purple circles indicating joint and purple arrows indicating spine and skull accumulation.

PET imaging was performed for all four radioligands [^64^Cu]Cu-**1**–**4** in nude mice, with PD-L1 positive and negative tumors in the right and left thigh, respectively (Figure 5). Two animals received only the tracer, while the other two animals were preinjected with either 500 or 1000 nmol of the respective, unlabeled compound.

For all compounds, PET images show a varying distribution pattern, with some unexpected accumulation in bone and joints (potentially the synovial space). This has not been found for our other compounds [48] with SUV_max_ (Table S1) in the respective regions usually well below 0.7 (1–2 h p.i.) and 0.4 (4–5 h p.i.). In contrast, all herein-reported compounds showed a much more pronounced uptake in bones and joints. This uptake was primarily observed in shoulder and/or acromioclavicular joints and hip and/or femorotibial joints. In contrast, no substantial uptake in other joints was observed, e.g., those of fore- and hind paws (carpus, tarsus). For the shoulder joint, SUV_max_ was between 1.63 and 2.55 at 1–2 h post-injection with [^64^Cu]Cu-**3** > [^64^Cu]Cu-**4** > [^64^Cu]Cu-**1** > [^64^Cu]Cu-**2**. Interestingly, for animals pre-injected with 500 nmol of respective cold compound (*n* = 2), joint uptake (SUV_max_) was found to be further increased between 3.36 ([^64^Cu]Cu-**4**) and 7.27 ([^64^Cu]Cu-**1**), an increase of 76% to 225% over tracer-only injected animals. Overall, compounds [^64^Cu]Cu-**1**&**2** exhibited a more pronounced bone uptake compared to [^64^Cu]Cu-**3** and [^64^Cu]Cu-**4** (Figure 5).

Excretion patterns also differed across tracer candidates. [^64^Cu]Cu-**2** showed the lowest initial liver uptake (SUV_max_~4.43), which was even lower under blocking conditions (SUV_max_ = 1.97). Early (1–2 h p.i.) renal clearance as defined by tracer uptake in the kidney, was low for [^64^Cu]Cu-**1**, [^64^Cu]Cu-**2** and [^64^Cu]Cu-**4**, while it was high (24.92) for [^64^Cu]Cu-**3**. Again, animals injected with [^64^Cu]Cu-**1** and [^64^Cu]Cu-**2** and 500 nmol of cold ligand showed a substantially increased renal uptake at 1–2 h p.i. (518 and 766%), likely caused by blocking uptake to the liver. In contrast, this pattern was not observed for [^64^Cu]Cu-**3** and [^64^Cu]Cu-**4**. Consequently, radioligand [^64^Cu]Cu-**1** showed a steadily high liver uptake (SUV_max_ > 4.4) over all time points, substantially reduced under blocking conditions.

Overall initial tumor uptake at 1.5 h p.i. was low (SUV_max_ < 2) for all compounds, with [^64^Cu]Cu-**1** and [^64^Cu]Cu-**4** showing the highest specific uptake (SUV_max_ = 1.6 and 1.8) and best contrast (152% and 135% higher uptake in PD-L1 vs. mock tumor). Blocking with 500 nmol of the cold respective compound showed a moderate reduction in tumor uptake between 18 and 43% for [^64^Cu]Cu-**1**, [^64^Cu]Cu-**2** and [^64^Cu]Cu-**4**. Together with the contrast of PD-L1 vs. mock tumor, this points to a moderate in vivo binding specificity of these compounds. In contrast, [^64^Cu]Cu-**3** did not show any blocking effect in the PD-L1 positive tumors.

Mock tumor uptake was largely unaffected by preinjection of an excess of cold ligand. For [^64^Cu]Cu-**3**, there was a moderate change (−21%), while all other compounds showed little ([^64^Cu]Cu-**4**, −16%) or no effect ([^64^Cu]Cu-**1** and [^64^Cu]Cu-**2**).

At 4.5 h p.i., no substantial increase in PD-L1 tumor uptake was noted compared to 1–2 h p.i., except for [^64^Cu]Cu-**3** (74%). All other compounds exhibited no change or a slight loss (−20 to −40%). However, presumed nonspecific uptake into the mock tumor increased for all compounds, from very little (5%) for [^64^Cu]Cu-**4** to substantial (135%) for [^64^Cu]Cu-**1**. Consequently, the contrast between PD-L1 and mock tumor decreased for all compounds except [^64^Cu]Cu-**3**. Notably, the effect of pre-injection of cold ligands seemed to increase over time for [^64^Cu]Cu-**1**, [^64^Cu]Cu-**2** and [^64^Cu]Cu-**3**, with reductions in tumor uptake >55%. This pattern continued at 24.5 h p.i., with the contrast between PD-L1 positive and mock tumors lost for all tracer candidates. Furthermore, tumor uptake generally increased, regardless of whether the target was expressed or not. Hence, it can be assumed that this accumulation reflects mostly unspecific binding. Interestingly, the blocking effect was still quite pronounced for [^64^Cu]Cu-**1**, [^64^Cu]Cu-**2** and [^64^Cu]Cu-**3**, with 50 to 90%.

## 3. Discussion

Immunotherapy targeting the PD-1/PD-L1 axis has opened a new treatment avenue for malignant tumors in the last decade. By reactivating the local immune response, cancer cells can be attacked and destroyed by the host’s immune cells.

However, decision on such a treatment would benefit from a better assessment of PD-L1 status in the whole tumor and over time, e.g., using PET or SPECT. The herein-reported PD-L1 radiotracers [^64^Cu]Cu-**1**–**4** were designed based on our experience that at least three hydrophilizing units are required to overcome the deep hydrophobic nature of the binding motif. Additionally, larger halogen atoms were associated with higher tumor uptake, which we wanted to test by the introduction of Cl, Br or I at the central, aromatic core.

The fast modification was achieved through our convergent route, leading to a variety of biphenyl-based PD-L1 ligands. After setting the stage by modifying the PD-L1 binding motif with an alkyne-functionality at the biphenyl core, we introduced all hydrophilizing units in the solvent-exposed region of the PD-L1 protein dimer. The alkyne underwent a CuAAC-reaction with the linker-chelator system to yield the final compounds **1**–**4**. We aimed at minimizing the loss of binding affinities and potentially increasing PD-L1 tumor uptake. Our previously reported ligands (log *D*_7.4_ values between −2.73 to −3.50) with exclusively sulfonic acids as hydrophilizing units showed extensive albumin binding capacities. Exchange of these sulfonic acids with phosphonic acids was thought to reduce albumin affinity, which we partially achieved, based on the affinity differences in the real-time binding. The modifications with sulfonic/phosphonic acids lead to deeply hydrophilic structures with log *D*_7.4_ values between −3.03 and −4.00. The substitution with iodine at the central aromatic core resulted in the highest log *D*_7.4_ value in this series, attributable to the hydrophobic nature of this bulky atom. Additionally, this did not strongly impact the binding affinity, neither positively nor negatively. That was demonstrated by both saturation and real-time binding and in comparison to the PD-L1 inhibitor BMS-1166,(IC_50_ = 1.4 nM) [49]. For the linear substitution pattern ([^64^Cu]Cu-**1** and [^64^Cu]Cu-**2**), affinity was lower in comparison to the branched substitution pattern ([^64^Cu]Cu-**3** and [^64^Cu]Cu-**4**) in the saturation binding assay, while no difference between both groups was observed in the real-time binding assay. Within these groups, the K_D_ values were in a similar range, indicating no remarkable influence of the halogen substituted in vitro. However, it was observed that the binding affinity decreased significantly upon iodine substitution, thereby indicating that the size of the bulky iodine atom exceeds the steric capacity of the PD-L1 dimeric tunnel [43,65].

The experiments with 2.5% BSA in the medium for all radioligands showed largely similar affinity as found in saturation binding, given the different nature of these techniques. However, it has to be noted that the curves in the presence of BSA show less curvature, especially for [^64^Cu]Cu-**2** and are derived from a single experiment only, hence they should be treated cautiously. In the absence of BSA, the increase in bound radioactivity at the beginning of both association phases is higher compared to the end of each phase which reflects the expected binding kinetics. For radioligand [^64^Cu]Cu-**2** and in the presence of BSA, the curve shows a different behavior: A sharp increase at the beginning of the association phase with only a small curvature indicating an instant binding, presumably to albumin/BSA. For the non-iodine substituted radioligands [^64^Cu]Cu-**1**, [^64^Cu]Cu-**3** and [^64^Cu]Cu-**4**, the curves in the presence of BSA are not dominated by this instantaneous, sharp increase at the beginning of each association phase. Based on the limited data, it could be concluded that these radioligands have a lower serum protein binding capacity compared to [^64^Cu]Cu-**2**. However, this hypothesis would have to be tested further, e.g., via gel electrophoresis separation in the presence of albumin or other methods.

In vivo, all four radioligands [^64^Cu]Cu-**1**–**4** showed largely similar and unexpected pharmacokinetic behavior. Copper-64 radiolabeling allowed us to investigate the pharmacokinetic profile up to 24 h post-injection, revealing that the uptake pattern at later time points was mainly driven by nonspecific binding. More importantly, a very pronounced uptake of all tracers was observed in joints and bones, the latter being also more pronounced at later time points. Liver uptake was relatively stable over the observed period. However, this does not indicate a primarily hepatobiliary clearance, as evidenced by the absence of tracer accumulation at early and mid-time points in the intestines. In contrast, intestinal radioactivity accumulation was observed for our first- and second-generations compounds [48]. Furthermore, the high hydrophilicity (log *D*_7.4_ between −3.03 and −4.00) of these compounds is not conducive to hepatobiliary clearance. Interestingly, initial renal clearance is only observed for [^64^Cu]Cu-**3**. This, together with the liver but lack of intestinal uptake would rather point to the tracers getting trapped in the liver. A possible explanation would be a first-pass effect of phosphonic acids in the liver or a macrophage-specific uptake there [66]. This is further substantiated by the fact that pre-injection of an excess of cold compound reduced tracer uptake to the liver and stimulated renal clearance, specifically for [^64^Cu]Cu-**1** and [^64^Cu]Cu-**2**. It implicates a specific binding to biological structures in the liver, as it seems to serve as a sink for the radioligands. When this sink is being saturated by a large amount of unlabeled compound, more free radioligand exists, allowing longer circulation times. Consequentially, it can be excreted renally (as found for [^64^Cu]Cu-**1** and [^64^Cu]Cu-**2**) or nonspecifically accumulate in joints, skull and spine. At the 4.5 h time point, no activity in the bladder is observed for all four radioligands (in contrast to the blocking condition), strengthening the hypothesis that the liver serves as a sink and lowers the fraction of the free radioligand drastically. Additionally, the circulation time of these compounds seems rather long, probably due to high plasma protein binding (e.g., albumin) which further limits the amount of free tracer available for tumor uptake. Together with a potential liver metabolization, this provides an explanation for the unspecific accumulation in both target-positive and negative tumors at 24 h, making future in vivo applications (e.g., large animal models) unlikely.

While the exact mechanism of joint and bone accumulation is not clear and not necessarily similar for joints and the skull/spine, a few mechanisms can be hypothesized. The joints, specifically the synovial membrane contain both synovial fibroblasts and resident synovial tissue macrophages. As the latter could accumulate phosphonic acids similar to those of the liver, a sufficiently sized joint could provide enough cells to provide an explanation for the tracer accumulation. However, as no PD-L1-specific radiolabeled antibody/peptide binding to joints has been reported in healthy but tumor-bearing animals, it is unlikely that the observed accumulation represents true binding. Besides the accumulation in the larger joints, some uptake to the skull and spine was observed as well. This may indicate a Ca^2+^-mediated uptake, which is well-described for bisphosphonates. Hence, we designed our compounds with this possible interaction in mind. By incorporating a greater spatial distance between both phosphonate groups, we hoped to avoid Ca^2+^-mediated uptake, e.g., into bones, rather than bridging both groups with just a methylene group. The spatial distance apparently plays a crucial role in influencing the Ca^2+^-affinity. While we have not performed a systematic analysis, our hypothesis is based on the in vivo distribution of our four candidates. For the radioligands with the branched bisphosphonate groups ([^64^Cu]Cu-**3**, [^64^Cu]Cu-**4**) accumulation in the skull, spine and joints is lower compared to the linearly incorporated phosphonic acid groups ([^64^Cu]Cu-**1** [^64^Cu]Cu-**2**). This is in contrast to the peptidomimetic radioligands for the vitronectin receptor reported by Harris et al. (2009). They introduced two directly linked phosphono-l-alanine and did not observe an accumulation in the liver or bones in ex vivo biodistribution of the ^177^Lu-labelled analog [67]. Hence, the incorporation of phosphonic acids into radiotracers could be an approach to enhancing hydrophilicity. However, its application necessitates individual study-based exploration. Target density and expression, tracer metabolization along with the employed mouse strain and its immunocompetency might influence the pharmacokinetic profile of phosphonic acid-containing radiotracers.

## 4. Materials and Methods

### 4.1. Radiochemistry

As part of the radiolabeling process, a 2 M stock solution of the corresponding precursor in DMSO was generated, using 8 nmol for in vitro and in vivo experiments. ^64^Cu was produced in-house through the nuclear reaction ^64^Ni(p,n)→^64^Cu, as previously reported in the literature [68]. The resulting ^64^Cu solution in 0.1–0.01 M HCl was mixed with 100–200 µL of a 1 M HEPES solution (pH of 4.5, adjusted with 1 M HCl). The precursor was then incubated with ^64^Cu at 50 °C for 10 min (at 300 rpm) in 1.5 mL DNA LoBind^®^ Eppendorf Tubes (Hamburg, Germany). A small aliquot was placed on iTLC-SG chromatography paper (Agilent, Santa Clara, California, United States), as the stationary phase and then developed with a 0.1 M citrate solution with a pH of 4 (adjusted with 1 M NaOH) as the mobile phase. The radioisotope thin layer analyzer (Rita Star, Elysia-Raytest GmbH, Straubenhardt, Germany) was used to analyze the chromatography paper. Labeling efficiency was determined by the ratio of unbound radiometal (R_f_ = 0.9) and radio-metal-complex (R_f_ = 0) by integration of the respective areas. All radiotracers achieved labeling efficiencies with ^64^Cu of >95%.

### 4.2. Log D_7.4_ Determination

The shake-flask method was used to determine the *n*-octanol/water distribution coefficients for each radioligand. Each experiment was performed in triplicate. 30 µL of the reaction mixture was combined with 570 µL of PBS (pH 7.4) and 600 µL of *n*-octanol in a 1.5 mL Eppendorf tube. The mixture was shaken at 1500 rpm (room temperature) for 5 min and then centrifuged. An aliquot was withdrawn from each phase, and radioactivity was measured using a γ-counter (ISOMED2160, NUVIA Instruments GmbH, Dresden, Germany). The mean values were calculated and corrected for the background activity. The log *D*_7.4_ value was calculated using the following formula:

$$\log D_{7.4}=\log(\frac{A_{n\text{-}octanol}}{A_{PBS}})$$

### 4.3. Kinetic Stability Studies (Human Serum)

A total of 8 nmol of the radiotracer was mixed with approximately 500 MBq of ^64^Cu in 50 µL of HEPES solution (adjusted to pH 4.5), as described above. After successful labeling, 450 µL of human serum (filtered) was added, and the solution was incubated at 37 °C under shaking at 300 rpm. At each time point (1, 24, and 48 h), an aliquot was withdrawn and added in a 1:2 ratio to a detergent mixture consisting of water containing 20% *v/v* EtOH, 5% *v/v* 5 mM aqueous EDTA, 0.5% *v/v* Triton X-100, 0.1% *m*/*v* saponin, and 0.05% *v/v* 0.5 mM aqueous *o*-phenanthroline to precipitate serum proteins. The suspension was cooled on ice for 5 min and then centrifuged for 5 min at 12,000× *g*. A small amount of the supernatant was analyzed by analytical radio-RP-HPLC (Kintetex^®^ 5 µm Phenyl-Hexyl 100 Å with 5–95% acetonitrile (0.1% TFA) in water (0.1% TFA) in a linear gradient over 16 min, 1 mL/min, γ-detection, Phenomenex, Aschaffenburg, Germany), and evaluation and graphical plotting were performed using OriginPro^®^ 9.0 (OriginLab, Northampton, MA, USA).

### 4.4. Cell Lines and Cell Culture

Prior to all experiments, PC3 cells were transduced to stably overexpress human PD-L1 (PC3-PD-L1). These cells were cultured along with PC3 cells transduced with a control plasmid (PC3-mock). Subsequently, all cells were cultured under identic conditions (RPMI-1640 medium (Sigma Aldrich, Darmstadt, Germany); (*v*/*v*) 10% fetal bovine serum (Sigma Aldrich), 1% penicillin/streptomycin (Gibco, Waltham, MA, USA), 1% nonessential amino acids and 1% alanine/glutamine, Sigma Aldrich) as described before [48] under normoxic (5% CO_2_; 37 °C) conditions and passaged upon reaching ~90% confluency. Maximum number of passages was 20. 

For saturation binding, cells were trypsinized (0.05% Trypsin-EDTA, Gibco), counted and diluted to ~160,000 cells/mL. 0.25 mL of this suspension was then seeded to each well of a 48-well plate (Falcon Multiwell #353078, Corning, Glendale, AZ, USA) and grown for at least 2 days. For real-time radioligand binding, 350,000–500,000 cells/mL were seeded into one side of petri dishes (in 3 mL), ~24 h before the experiments.

### 4.5. Saturation Binding Studies

On the day of the experiments, cells were brought to room temperature and then cooled on ice (each 15 min). All medium was removed and replaced with 200 µL assay buffer [PBS + 2.5% (*w*/*v*) bovine serum albumin (Carl Roth, Karlsruhe, Germany) for total binding (TB). For determining the nonspecific binding, assay buffer containing 300 µM of BMS-1166 (in DMSO, resulting in 0.03% *v*/*v*) was used and cells were incubated for 15 min. Thereafter, 200 µL of eight serial 1:1 dilutions (500 to 3.91 nM) of the respective radioligand were added (in triplicate) and incubated for 90 min at 4 °C. This was followed by washing (3 × 1 min) and lysis (500 µL 0.1 M NaOH + 1%SDS). Radioactivity of the lysate was determined by gamma counting and decay corrected to a reference time (end of radiolabeling). Using the same conditions but lacking radioligand and BSA-buffer, an additional plate served as protein control. The protein content of these lysates was determined by bicinchoninic acid assay (BCA). Together with counts per minute (CPM) measurements, final values (pmol/mg protein) were calculated using mean protein content and molar activity. For each compound, three individual experiments were performed.

Processed data were analyzed using non-linear iterative curve fitting (GraphPad Prism 9, GraphPad Software, Boston, MA, United States) providing K_D_ (in nM) and B_max_ (in pmol/mg protein).

### 4.6. Real-Time Radioligand Binding Studies

A LigandTracer Yellow (Ridgeview Instruments AB, Uppsala, Sweden) was utilized for real-time binding. This allowed us to assess kinetics (association rate constant k_a_ and dissociation rate constant k_d_) and dissociation constant (K_D_). Previously prepared cells in culture dishes were subjected to two increasing concentrations of the radioligand (10 and 40 nM, 90 min each) in a CO_2_-independent medium at room temperature (either with or without 2.5% BSA). This was followed by dissociation for at least 120 min, where the radioligand solutions were exchanged with fresh medium. The binding curves (traces) were acquired in decay-corrected counts per second (CPS) and evaluated using TraceDrawer 1.9.2 (Ridgeview Instruments AB, Uppsala, Sweden). Data were checked, potential spikes removed and then normalized to their own baseline (=0%) and the highest value (=100%). The obtained traces were then fitted using a one-to-one interaction accounting for bulk effect.

### 4.7. Animals and PET Imaging

Athymic NMRI-nude mice (male, Rj:NMRI-Foxn1nu, Janvier Labs, Le Genest-Saint-Isle, France) of 8 to 22 weeks of age were used. General anesthesia (~10% desflurane in 30 vol% oxygen + air) was induced under gentle warming. Subsequently, 3–5 × 10^6^ PC3-PD-L1 and PC3-mock cells [in 50 µL PBS + 50 µL Matrigel, (Corning, Glendale, CA, USA)], were subcutaneously injected into the right and left thigh. Growth was monitored thrice a week and once tumors reached 7 mm, animals were included in the PET cohort. 

For this, animals were again anesthetized, and PET/X-ray computed tomography (CT) was performed (nanoScan PET/CT, Mediso, Münster, Germany). CT images served as attenuation correction and anatomical referencing. All animals received three individual scans: 0–2 h, 4–5 h and 24–25 h post-injection. The tracers were dissolved in 300 µL sterile 0.9% NaCl/HEPES buffer, pH 6–7 and delivered via the lateral tail vein over 30 s. PET acquisition was started with begin of tracer infusion. Molar activities were above 12 GBq/µmol and injected activities (mean MBq ± SD) were for [^64^Cu]Cu-**1**: 12.20 ± 0.59 MBq, [^64^Cu]Cu-**2**: 12.37 ± 0.84 MBq, [^64^Cu]Cu-**3**: 10.49 ± 0.52 MBq and [^64^Cu]Cu-**4**: 12.17 ± 0.36 MBq. 

Three-dimensional list mode data were recorded and binned using the 400–600 keV energy window. For 0–2 h p.i., data were sorted into 35-time frames (15 × 10 s, 5 × 30 s, 5 × 60 s, 4 × 300 s, 3 × 600 s, 6 × 600 s and 3 × 1200 s). Frames were reconstructed with a voxel size of 0.4 mm, correcting for decay, scatter, and attenuation. Images were post-processed and analyzed using Rover (ABX GmbH, Radeberg, Germany) and displayed as maximum intensity projections (MIPs) at indicated time points and scaling.

Three-dimensional volumes of interest were delineated by applying fixed thresholding. Standardized uptake values (SUV = [MBq detected activity/mL tissue]/[MBq injected activity/g body weight], mL/g) were determined in selected volumes of interest (PD-L1 and mock tumor, liver, kidney and left shoulder joint).

### 4.8. Data and Statistical Analysis

Data are presented as mean values ± standard deviation. Plotting and nonlinear curve fitting were performed with GraphPad Prism 9. 

### 4.9. Synthesis & General Remarks

Experimental procedures and analytical data for literature unknown compounds are provided in the Supplementary Information (Figures S1–S36: NMR spectra, S37–S55: IR spectra, S56–S72: HPLC chromatograms, S73–S94: MS spectra)

All manipulations that required the exclusion of oxygen and moisture were carried out under an argon gas atmosphere in heat-gun dried flasks utilizing the Schlenk technique. Solvents and chemicals were purchased from Sigma-Aldrich Laborchemikalien GmbH (Taufkirchen, Germany), Acros Organics (Waltham, MA, USA), abcr GmbH (Karlsruhe, Germany), and Fisher Scientific (ThermoFisher Scientific, Waltham, MA, USA) and were used without any purification. For NMR studies, deuterated solvents were purchased from Deutero GmbH (Kastellaun, Germany). The anhydrous solvents DMF, dichloromethane, tetrahydrofuran and methanol were purchased from Sigma–Aldrich Laborchemikalien GmbH (Sure/Seal™ bottles). Thin-layer chromatography (TLC) analysis was performed on Merck pre-coated plates (silica gel 60 F_254_, Art 5715, 0.25 mm, Darmstadt, Germany) and visualized was accomplished with UV light or KMNO_4_ stain. Attenuated Total Reflectance Fourier Transform Infrared (ATR-FTIR) spectra were recorded with a Thermo Scientific Nicolet™ iS™ 5 FT-IR-device and band intensity was described as weak (w), medium (m), strong (s) and broad signal (bs). NMR spectroscopy was performed with an Agilent DD2-400 MHz NMR or an Agilent DD2-600 MHz NMR spectrometer with ProbeOne (Agilent, Santa Clara, CA, USA). Chemical shifts of ^1^H, ^13^C and ^31^P signals were reported in parts per million (ppm) at 25 °C with TMS as the internal standard. Spectra were calibrated to the respective solvent signal. High-resolution mass spectra (HR-MS) were acquired on a TOF (Q-TOF MS; electrospray ionization), Agilent 1260 Infinity II HPLC (Santa Clara, CA, USA; pump G7111B, autosampler G7129A, column oven G7116N, UV detector G7717C, eluent acetonitrile/water acidified with 0.1% formic acid, bypass mode) coupled to UHD Accurate Mass Q-TOF LC MS G6538A. Analytical reversed HPLC was conducted on an Agilent C18 column (Agilent Zorbax 300SB-C18, 100 mm × 4.6 mm, Agilent, Santa Clara, CA, USA)) with acetonitrile/water (0.1% TFA each) as mobile phase. Preparative and semi-preparative reversed HPLC separations were performed on the Knauer (Berlin, Germany) Azura on Zorbax SB C-18 5 μm 80 Å, 9.4 × 250 mm as a stationary phase with acetonitrile/water (0.1% TFA each) as mobile phase.

## 5. Conclusions

In conclusion, we synthesized a set of four novel radioligands for PET imaging of PD-L1 expression. Along a NODA-GA chelator, we introduced three hydrophilic units (one sulfonic and two phosphonic acids) to the binding motif with two different substitution patterns to improve the pharmacokinetic profile of the deeply hydrophobic lead structure. Furthermore, we tested whether a halogen atom at the central aryl core could improve binding affinity and tumor uptake. After labeling with ^64^Cu, the proteolytic stability of these radioligands was confirmed. We managed to further reduce hydrophobicity, an important property of whole-body imaging agents. Binding affinities were minimally affected by the structural changes, as evidenced by low two-digit nM affinity. However, the structural modifications resulted in an unexpected pharmacokinetic in vivo profile. Despite low log *D*_7.4_ values, high liver accumulation along with bone, bone marrow, and joint uptake was observed. Both effects were attributed to the phosphonic acid-mediated binding to macrophages and/or their affinity toward calcium ions. In future studies, the partial substitution of highly hydrophilic phosphonic acids with carboxylic and/or sulfonic acids could be explored to develop biphenyl-based PD-L1 radioligands with higher tumor uptake.

## 6. Patents

F.K. and S.S. are inventors of the European patent application EP21212444 (submitted on 6 December 2021) and EP23167021.7 (submitted on 6 April 2023) for biphenyl-based imaging and therapy agents targeting PD-L1.

## Figures and Tables

**Table 1:** Summary of in vitro data (K_D_ and B_max_), log *D*_7.4_ values and molar activities used for the assay for the radioligands [^64^Cu]Cu-**1**–**4**.

Radioligand	K_D_ [nM] ^1^	B_max_ [pmol × mg^−1^] ^1^	log *D*_7.4_ ^2^	A_M_ [GBq × µmol^−1^]
[^64^Cu]Cu-**1**	190.7 ± 10.2	7.50 ± 0.22	−4.00 ± 0.14	13.5 ± 0.02
[^64^Cu]Cu-**2**	154.1 ± 26.2	1.97 ± 0.23	−3.03 ± 0.05	12.9 ± 0.05
[^64^Cu]Cu-**3**	76.7 ± 23.5	2.81 ± 0.32	−3.80 ± 0.02	13.1 ± 0.02
[^64^Cu]Cu-**4**	69.6 ± 8.10	2.22 ± 0.18	−3.81 ± 0.08	13.0 ± 0.03

^1^ Affinity (dissociation constant K_D_) and maximum number of binding sites (B_max_) as determined under identical conditions in a saturation binding assay using live PC3 cells overexpressing PD-L1. Data is mean ± (SD), derived from at least three independent experiments (each in triplicate), ^2^ Data is mean ± SD, derived from three separate shaking flask experiments.

**Table 2:** Kinetic parameters (association rate constant k_a_, dissociation rate constant k_d_ and dissociation constant K_D_) for radioligands [^64^Cu]Cu-**1**–**4**.

Radioligand	k_a_ [(M × s)^−1^]	k_d_ [s^−1^]	K_D_ [nM]	B_max_ [%]
medium only (*n* = 2; mean ± S.D.)				
[^64^Cu]Cu-**1**	(4.84 ± 0.38) × 10^3^	(6.48 ± 0.20) × 10^−5^	13.5 ± 0.64	137.8 ± 4.06
[^64^Cu]Cu-**2**	(0.38 ± 0.07) × 10^3^	(10.5 ± 0.28) × 10^−5^	279 ± 45.3	949 ± 140
[^64^Cu]Cu-**3**	(3.74 ± 0.14) × 10^3^	(6.50 ± 0.17) × 10^−5^	17.4 ± 0.14	135.6 ± 8.22
[^64^Cu]Cu-**4**	(4.68 ± 0.73) × 10^3^	(5.83 ± 0.64)×10^−5^	12.7 ± 3.39	118 ± 27.5
medium + 2.5% BSA (*n* = 1)				
[^64^Cu]Cu-**1**	10.2 × 10^2^	1.09 × 10^−4^	107	382.6
[^64^Cu]Cu-**2**	6.14 × 10^2^	1.47 × 10^−4^	239	224
[^64^Cu]Cu-**3**	5.42 × 10^2^	0.77 × 10^−4^	143	567.3
[^64^Cu]Cu-**4**	9.10 × 10^2^	0.79 × 10^−4^	86.9	328.2

## Data Availability

All data of this study are presented in the manuscript and Supplementary Material.

## Supplementary Materials

The following supporting information can be downloaded at: https://www.mdpi.com/article/10.3390/ijms242015088/s1. Copies of ^1^H and ^13^C NMR spectra, IR spectra, HPLC chromatograms and biological data of intermediate and final compounds.
