# Supplementary material for: Exploring Hydrophilic PD-L1 Radiotracers Utilizing Phosphonic Acids: Insights into Unforeseen Pharmacokinetics
Source: Int J Mol Sci. 2023 Oct 11;24(20):15088. doi: 10.3390/ijms242015088 (PMC10606431; doi:10.3390/ijms242015088)
Supplement: Supplementary file 1 [file ijms-24-15088-s001.zip › ijms-2638568-supplementary.pdf]

Exploring Hydrophilic PD-L1 Radiotracers Utilizing Phosphonic Acids: Insights Into Unforeseen Pharmacokinetics

Fabian Krutzek [1], Cornelius K. Donat [1], Sven Stadlbauer [1,2,*]

[1] Helmholtz-Zentrum Dresden-Rossendorf, Institute of Radiopharmaceutical Cancer Research, Bautzner Landstraße 400, 01328 Dresden, Germany

[2] School of Science, Faculty of Chemistry and Food Chemistry, Technical University Dresden, 01069 Dresden, Germany

Content

1. Organic Synthesis 2

1.1 General Procedures 2

1.2 HPLC-Systems 3

1.3 Synthetic Procedures 3

2. ^{1}H and ^{13}C NMR spectra of unknown compounds 20

3. IR spectra of literature unknown compounds 38

4. HPLC-chromatograms of HPLC purified compounds 45

5. Mass spectra of literature unknown compounds 51

6. Biological data 65

1. Organic Synthesis

Supplier for reagents: *N*,*N*-Diisopropylethylamine (DIPEA, ≥99.%), *N*,*N*-Dimethylformamide (DMF, ≥99.8%), trifluoroacetic acid (TFA, ≥99%), methanol (MeOH, ≥99.8%) and *tert*-butanol (^{t}BuOH, ≥99%) were purchased from Sigma Aldrich (Taufkirchen, Germany). Triethylphosphite (>98%) and (*R*)-2-((((9*H*-fluoren-9-yl)methoxy)carbonyl)amino)-3-iodopropanoate (≥98%) were purchased from Sigma Aldrich (Taufkirchen, Germany). 2-(1*H*-benzotriazol-1-yl)-1,1,3,3-tetramethyluronium hexafluorophosphate (HTBU, >98%) was purchased from BLDPharm (Reinbek, Germany). Sodium azide (≥99.5%), potassium carbonate (>98%), triphenylphosphine (>99%), di-2-methoxyethyl azodicarboxylate (DMEAD, >90%) and L-cysteic acid (>99%) were purchased from Sigma Aldrich (Taufkirchen, Germany). Sodium cyanoborohydride (>95%) was purchased from Acros Organics (Schwerte, Germany). 4-(4,7-Bis(2-(*tert*-butoxy)-2-oxoethyl)-1,4,7-triazacyclononan-1-yl)-5-(*tert*-butoxy)-5-oxopentanoic acid ((*R*)-NODAGA(^{t}Bu)$_3$) was purchased from CheMatech (Dijon, France). [Bis(dimethylamino)methylene]-1*H*-1,2,3-triazolo[4,5-b]pyridinium 3-oxid hexafluorophosphate (HAUT, >98%) was purchased from BLDPharm (Reinbek, Germany). Tris(3-hydroxypropyltriazolylmethyl)amine (THPTA, ≥97%) was purchased from Carl Roth (Karlsruhe, Germany). Copper(II)-sulfate pentahydrate (≥99%), (+)-sodium-L-ascorbate (≥98%) and bromotrimethylsilan (97%) were purchased from Sigma Aldrich (Taufkirchen, Germany).

1.1 General Procedures

Amide bond formation 1.1.1

The carboxylic acid, base, coupling reagent and HOBt were dissolved in abs. DMF and stirred at room temperature for 5 min. After cooling to 0 °C, the amine was added. The reaction progress was monitored via analytical RP-HPLC (system A). After complete conversion, the solvent was removed, and the residue was purified with semi-preparative RP-HPLC. After lyophilization the amide was obtained.

Fmoc-Deprotection 1.1.2

The Fmoc-protected compound (1.0 eq.) was dissolved in abs. DMF, sodium azide (5 eq.) was added and the reaction mixture stirred at 60 °C for 3 h. After complete conversion (monitored via analytic RP-HPLC, system A), the solvent was removed *in vacuo*. The residue was purified with semi-preparative RP-HPLC and after lyophilization, the deprotected amine was obtained.

Reductive Amination 1.1.3

The aldehyde (1.0 eq.) and the amine (3.0–5.0 eq.) were dissolved in the appropriate solvent mixture and stirred at room temperature for 10 minutes. The reaction mixture was then cooled

to 0°C and $NaBH_3CN$ (1.5 eq.) was added in small portions. The reaction mixture was stirred at room temperature for the appropriate time, solvent then removed under vacuum. Purification was performed by semi-preparative RP-HPLC and after lyophilization, the compound was obtained.

Global Deprotection 1.1.4

For global deprotection of phosphonic and carboxylic acids, the protected compound was dissolved in abs. DMF in a NMR tube in argon atmosphere. TMSBr (20 eq.) was added and the reaction was monitored with ^{31}P NMR spectroscopy. After formation of bis(trimethylsilyl)phosphonate was completed, 50 µL of MeOH was added. In order to hydroylyze TMS-esters, the NMR tube was shaken, which was then confirmed by ^{31}P NMR spectroscopy. The solvent was removed *in vacuo* and the deprotection cocktail (TFA:CH_2Cl_2:TES:H_2O, 20:20:8:7, *v*/*v*) added. The reaction mixture was stirred at room temperature for at least 40 h. Complete deprotection was confirmed with analytical RP-HPLC (system B) and the solvent was then removed under reduced pressure. Purification with semi-preparative RP-HPLC with subsequent lyophilization provided the final compound as a colorless powder.

1.2 HPLC-Systems

System A: RP-HPLC, analytical (Agilent Zorbax 300 C-18, 5 µm, 4,6 x 150 mm) with 10–95% MeCN (0.1% TFA) in H_2O (0.1% TFA) with a linear gradient over 15 min, 1 mL/min.

System B: RP-HPLC, analytical (Agilent Zorbax 300 C-18, 5 µm, 4,6 x 200 mm) with 10–95% MeCN (0.1% TFA) in H_2O (0.1% TFA) with a linear gradient over 30 min, 1 mL/min.

System C: RP-HPLC, semi-preparative (Agilent Zorbax SB C-18 5 µm 80 Å, 9,4 x 250 mm) with 25–80% MeCN (0.1% TFA) in H_2O (0.1% TFA) with a linear gradient over 45 min, 6 mL/min.

System D: RP-HPLC, semi-preparative (Agilent Zorbax SB C-18 5 µm 80 Å, 9,4 x 250 mm) with 40–90% MeCN (0.1% TFA) in H_2O (0.1% TFA) with a linear gradient over 45 min, 6 mL/min.

1.3 Synthetic Procedures

*Synthesis of (R)-2-((((9H-Fluoren-9-yl)methoxy)carbonyl)amino)-3-(diethoxyphosphoryl)propanoic acid (**25**)*

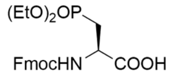

Triethyl phosphite (3.98 mL, 23.2 mmol, 5.00 equiv.) was degassed (argon, 30 min) and then *tert*-butyl (*R*)-2-((((9*H*-fluoren-9-yl)methoxy)carbonyl)amino)-3-iodopropanoate (2.03 g, 4.64 mmol, 1.00 equiv.) added. The solution was stirred at 140 °C for 16 h in an oxygen-free atmosphere. Subsequently, all

volatiles were removed by vacuum distillation (90 °C, 10^{-3}mbar) and the yellowish residue was used in the next step without further purification. It was dissolved in a 1:1 mixture of TFA/DCM (20 mL) and stirred at room temperature for 5 h. All solvents were removed *in vacuo*, followed by addition of DCM (200 mL) and sodium bicarbonate solution (400 mL) to adjust the aqueous phase to pH 8. The phases were separated, the aqueous one was washed with DCM (150 mL) and then adjusted to pH 2 with 1 M hydrochloric acid. The aqueous phase was extracted with DCM (3×200 mL), the combined organic extracts were dried over sodium sulfate, filtered and concentrated under reduced pressure. Flash column chromatography on SiO_2 (EA:MeOH:AcOH, 93:7:1, R_f = 0.27) yielded **25** (1.04 g, 2.33 mmol, 57% over two steps) as a yellowish oil. R_t = 10.90 min (system A), purity = 100%. ^{1}H NMR (400 MHz, CD_2Cl_2) δ = 7.73 (d, ^3J = 7.5 Hz, 2H), 7.54–7.56 (m, 2H), 7.35 (t, ^3J = 7.4 Hz, 2H), 7.24 (t, ^3J = 7.2 Hz, 2H), 4.02–4.46 (m, 7H), 1.20–1.22 ppm (m, 6H). ^{13}C NMR (101 MHz, CD_2Cl_2) δ = 176.7, 156.9, 144.4, 141.8, 128.2, 127.6, 125.8, 120.4, 67.8, 63.6 (d, ^2J = 33.5 Hz), 51.0, 25.5 (d, ^1J = 150.2 Hz), 16.5 ppm (d, ^3J = 5.6 Hz). ^{31}P NMR (162 MHz, CD_2Cl_2) δ = 28.7 ppm. IR (ATR): $\tilde{\nu}$ = 1682 (s), 1607 (m), 1519 (w), 1448 (m), 1206 (s), 1137 (s), 1023 (s), 971 (w), 840 (w), 799 (w), 759 (w), 738 (m), 722 cm^{-1} (m). MS (HR-ESI$^+$): Exact mass calculated for $[M+H]^+$: m/z = 448.1480, measured: m/z = 448.1518.

*Synthesis of (9H-Fluorene-9-yl)methyl (R)-(3-(diethoxyphosphoryl)-1-((2-(diethoxy-phosphoryl)ethyl)-amino)-1-oxopropane-2-yl)carbamate (**27**)*

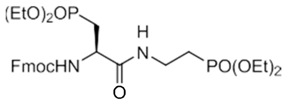

The carboxylic acid **25** (1.22 g, 2.73 mmol, 1.00 eq.), diethyl(2-aminoethyl)phosphonate **26**, HBTU (0.54 g, 3.00 mmol, 1.10 eq.), HOBt (0.37 g, 2.73 mmol, 1.00 eq.) and abs. DIPEA (0.95 mL, 5.46 mmol, 2.00 eq.) were reacted in abs. DMF (5 mL) according to procedure 1.1.1. The solvent was removed under vacuum and the crude product was purified on SiO_2 (EtOAc:MeOH, 90:10, R_f = 0.15), yielding compound **27** (1.45 g, 2.38 mmol, 87%) as a yellow oil.

R_t = 11.10 min (system A), purity (HPLC) = 95.5%. ^{1}H-NMR (400 MHz, MeCN-d_3) δ = 7.84 (d, ^3J = 7.5 Hz, 2H), 7.68 (d, ^3J = 7.2 Hz, 2H), 7.42 (t, ^3J = 7.5 Hz, 2H), 7.34 (t, ^3J = 7.4 Hz, 2H), 7.16 (bs, 1H), 6.31 (d, ^3J = 7.2 Hz, 1H), 4.37–4.40 (m, 2H), 4.25–4.27 (m, 2H), 3.97–4.06 (m, 8H), 3.35–3.40 (m, 2H), 2.11–2.32 (m, 3H), 1.93–1.95 (m, 4H), 1.22–1.28 ppm (m, 12H). ^{13}C-NMR (101 MHz, MeCN-d_3) δ = 171.3, 171.2, 156.8, 145.1, 142.2, 128.7, 128.1, 126.2, 121.0, 67.5, 62.6–63.0 (m), 51.4, 48.1, 34.5 (d, ^3J = 2.6 Hz), 28.2 (d, ^2J = 141.1 Hz), 26.2 ppm (d, ^2J = 138.2 Hz). ^{31}P-NMR (162 MHz, MeCN-d_3) δ = 28.5 (s, 1H), 27.4 ppm (s, 1H). IR (ATR): v = 1673 (m), 1525 (w), 1449 (w), 1208 (m), 1023 (s), 968 (m), 842 (s), 733 (s), 701 (w), 558 cm^{-1} (w). MS (HR-ESI+): Exact mass calculated for $[M+H]^+$: m/z = 611.2287, measured:

m/z = 611.2278.

*Synthesis of Diethyl (R)-(2-amino-3-((2-(diethoxyphosphoryl)ethyl)amino)-3-oxopropyl)phosphonate (**13**)*

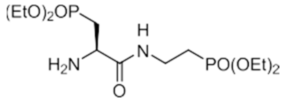

The compound **27** (850 mg, 1.39 mmol, 1.00 eq.) was dissolved in abs. DMF (10 mL) and the Fmoc deprotection was carried out using sodium azide (452 mg, 6.95 mmol, 5.00 eq.) according to procedure 1.1.2. Column chromatography on basic alumina (EtOAc then EtOH/MeCN, 1:1 R_f = 0.2) afforded compound **13** (300 mg, 0.77 µmol, 56%) as a yellowish oil.

^{1}H-NMR (400 MHz, CD_3OD) δ = 4.10–4.16 (m, 8H), 3.56–3.63 (m, 1H), 3.42–3.49 (m, 2H), 2.31–2.40 (m, 1H), 2.00–2.13 (m, 3H), 1.32–1.36 ppm (m, 12H). ^{13}C-NMR (101 MHz, CD_3OD) δ = 175.8, 63.4–63.6 (m), 51.7 (d, ^3J = 4.0 Hz), 34.6 (d, ^3J = 2.3 Hz), 31.5 (d, ^2J = 141.2 Hz), 26.2 (^2J = 139.2 Hz), 16.7–16.7 ppm, (m). ^{31}P-NMR (162 MHz, CD_3OD) δ = 29.6 (s, 1H), 29.4 ppm (s, 1H). IR (ATR): v = 1982 (w), 1661 (m), 1524 (w), 1393 (w), 1223 (m), 1163 (w), 1019 (s), 957 (s), 836 (w), 785 cm^{-1} (w). MS (ESI+): Mass calculated for $[M+H]^+$: *m/z* = 389.1, measured: *m/z* = 389.1.

*Synthesis of 5-Bromo-4-((2-methoxyethoxy)methoxy)-2-((5-(methylsulfonyl)pyridin-3-yl)methoxy)-benzaldehyde (**24**)*

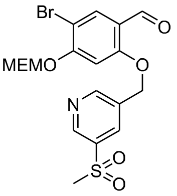

5-Bromo-2-hydroxy-4-((2-methoxyethoxy)methoxy)benzaldehyde (**21**) (371 mg, 1.21 mmol, 1.00 eq.) was reacted with 3-(bromomethyl)-5-(methylsulfonyl)pyridine (**22**) (272 mg, 1.33 mmol, 1.10 eq.) and potassium carbonate (251 mg, 1.82 mmol, 1.50 eq.) in abs. DMF (10 mL) at room temperature for 16 h. The solvent was removed, and purification conducted by column chromatography on SiO_2(EE, R_f = 0.3) to provide phenol **24** (474 mg, 0.98 mmol, 81%) as a colorless solid.

m.p. = 138–141 °C. ^{1}H-NMR (400 MHz, $CDCl_3$) δ = 10.26 (s, 1H), 8.40 (s, 1H), 8.04 (s, 1H), 6.99 (s, 1H), 5.42 (s, 2H), 5.29 (s, 1H), 3.88–3.90 (m, 2H), 3.57–3.59 (m, 2H), 3.37 (s, 3H), 3.16 ppm (s, 3H). ^{13}C-NMR (101 MHz, $CDCl_3$) δ = 186.6, 160.6, 159.8, 134.0, 120.8, 150.7, 100.9, 94.3, 71.5, 68.5, 59.2, 59.1, 45.0 ppm. IR (ATR): v = 1674 (m), 1660 (s), 1594 (s), 1572 (m), 1420 (m), 1383 (m), 1316 (m), 1305 (m), 1269

(s), 1171 (m), 1135 (s), 1105 (s), 1052 (m), 1005 (m), 943 (m), 859 (m), 768 (m), 696 (m), 636 cm^{-1} (w). MS (ESI+): Mass calculated for $[M+H]^+$: *m/z* = 474.01, measured: *m/z* = 474.18.

*Synthesis of 5-Bromo-4-hydroxy-2-((5-(methylsulfonyl)pyridine-3-yl)methoxy)benzaldehyde (**17**)*

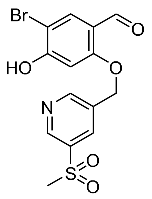

The MEM-protected phenol **24** (460 mg, 970 µmol, 1.00 eq.) was dissolved in abs. CH_2Cl_2 (10 mL) and the solution was cooled to 0°C. After addition of TFA (5 mL), the reaction mixture was stirred at room temperature for 16 h. EtOAc (100 mL) was added and the resulting solid was isolated by filtration. The resulting pale-yellow substance was identified as aldehyde **17** (355 mg, 919 mmol, 94%), which was used in the next step without further purification.

m.p. = 228–232°C °C. ^{1}H-NMR (400 MHz, DMSO-d_6) δ = 11.66 (s, 1H), 10.12 (s, 1H), 9.09 (s, 1H), 9.08 (s, 1H), 8.47 (s, 1H), 7.81 (s, 1H), 6.79 (s, 1H), 5.38 (s, 2H), 3.37 ppm (s, 3H). ^{13}C-NMR (101 MHz, DMSO-d_6) δ = 186.3, 160.9, 160.9, 153.2, 147.4, 137.0, 134.2, 132.7, 118.4, 102.2, 101.2, 67.0, 43.6 ppm. IR (ATR): v = 1688 (m), 1668 (m), 1596 (m), 1516 (w), 1450 (m), 1406 (m), 1396 (s), 1367 (m), 1302 (s), 1283 (m), 1213 (s), 1187 (s), 1141 (s), 1065 (w), 1018 (w), 970 (m), 837 (w), 816 (m), 774 (m), 762 (m), 692 (m), 555 cm^{-1} (w). MS (ESI+): Mass calculated for [M+H]+: *m/z* = 385.97, measured: *m/z* = 386.08.

*Synthesis of 5-Bromo-4-((2,2'-dimethyl-3'-(prop-2-yn-1-yloxy)-[1,1'-biphenyl]-3-yl)-methoxy)-2-((5-(methylsulfonyl)pyridine-3-yl)methoxy)benzaldehyde (**11**)*

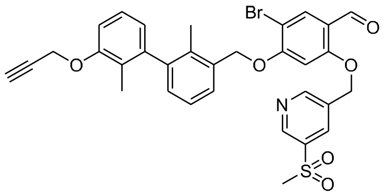

(2,2'-Dimethyl-3'-(prop-2-yn-1-yloxy)-[1,1'-biphenyl]-3-yl)methanol (**15**) (100 mg, 376 µmol, 1.00 eq.), phenol **17** (174 mg, 451 µmol, 1.20 eq.), and triphenylphosphine (118 mg, 451 µmol, 1.20 eq.) were dissolved in abs. DMF (3 mL) and reacted with DMEAD (106 mg, 451 µmol, 1.20 eq.) at 0 °C for 30 min. Thereafter, the reaction mixture was allowed to reach room temperature and was stirred for additional 16 h. The solvent was removed and purification was performed with column chromatography on SiO_2 (CH_2Cl_2:EtOAc, 90:10, R_f = 0.22) to provide compound **11** (155 mg,

244 µmol, 65%) as a colorless solid.

m.p. = 179–180°C. ^{1}H-NMR (400 MHz, $CDCl_3$) δ = 10.24 (s, 1H), 9.18 (s, 1H), 9.00 (s, 1H), 8.41 (s, 1H), 8.08 (s, 1H), 7.48 (d, ^3J = 7.4 Hz, 1H), 7.16–7.29 (m, 3H), 6.99 (d, ^3J = 8.1 Hz, 1H), 6.80 (d, ^3J = 7.5 Hz, 1H), 6.67 (s, 1H), 5.26 (s, 4H), 4.76 (s, 2H), 3.17 (s, 3H), 2.54 (s, 1H), 2.10 (s, 3H), 1.93 ppm (s, 3H). ^{13}C-NMR (101 MHz, $CDCl_3$) δ = 186.5, 161.0, 160.9, 156.0, 153.1, 148.6, 142.8, 142.6, 137.5, 134.8, 134.6, 134.3, 133.3, 132.4, 130.2, 127.5, 126.1, 125.9, 125.6, 122.8, 120.1, 119.1, 110.7, 105.8, 98.6, 79.1, 75.5, 70.7, 68.0, 56.3, 45.0, 15.9, 13.1 ppm. IR (ATR): ν = 3270 (m), 1659 (s), 1589 (s), 1568 (m), 1456 (m), 1409 (m), 1374 (w), 1305 (s), 1271 (s), 1192 (m), 1172 (m), 1135 (s), 1018 (m), 957 (w), 769 (w), 699 cm^{-1} (w). MS (ESI+): Mass calculated for $[M+H]^+$: m/z = 634.1, measured: m/z = 634.0.

*Synthesis of (5-Bromo-4-((2,2'-dimethyl-3'-(prop-2-yn-1-yloxy)-[1,1'-biphenyl]-3-yl)- methoxy)-2-((5-(methylsulfonyl)pyridine-3-yl)methoxy)benzyl)(sulfo)-D-alanin (**35**)*

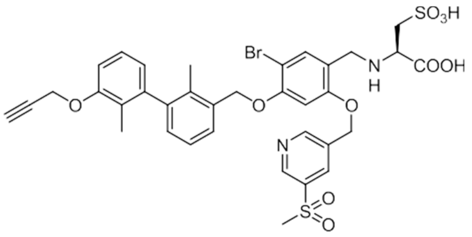

The aldehyde **11** (160 mg, 252 µmol, 1.00 eq.), L-cysteic acid (128 mg, 756 µmol, 3.00 eq.), and $NaBH_3CN$ (23.8 mg, 378 µmol, 1.50 eq.) were reacted in a mixture of abs. MeOH/DMF (2 mL) according to procedure 1.1.3. After reaction was completed (analytical RP-HPLC, system A), the solvent was removed under vacuum and the residue was purified by semi-preparative RP-HPLC (Agilent Zorbax SB C-18 5 µm 80 Å, 9.4 x 250 mm with 45–90% MeCN (0.1% TFA) in H_2O (0.1% TFA) in a linear gradient over 45 min, 6 mL/min, R_t = 6 min). Subsequent lyophilization yielded the carboxylic acid **35** (81.0 mg, 103 µmol, 41%) as a colorless powder.

m.p. = 160–163 °C. R_t = 12.20 min (system A), purity (HPLC) = 94.2%. ^{1}H-NMR (400 MHz, DMSO-d_6) δ = 9.33 (bs, 1H), 9.10 (s, 1H), 9.07 (s, 1H), 8.51 (s, 1H), 7.67 (s, 1H), 7.50 (d, ^3J = 7.6 Hz, 1H), 7.21–7.29 (m, 3H), 7.04–7.10 (m, 2H), 6.74 (d, ^3J = 7.5 Hz, 1H), 5.47 (s, 2H), 5.31 (s, 2H), 4.85 (s, 2H), 4.20–4.32 (m, 3H), 3.59 (s, 1H), 3.36 (s, 3H), 3.03–3.07 (m, 1H), 2.87–2.94 (m, 1H), 2.04 (s, 3H), 1.83 ppm (s, 3H). ^{13}C-NMR (101 MHz, DMSO-d_6) δ = 168.4, 157.1, 156.4, 155.4, 153.5, 147.2, 142.3, 141.5, 136.9, 135.3, 134.8, 134.6, 134.5, 132.7, 129.3, 127.7, 126.2, 125.2, 124.1, 122.1, 113.7, 110.9, 79.5, 78.2, 69.8, 67.3, 56.3, 55.8, 48.6, 44.6, 43.6, 15.4, 12.8 ppm. IR (ATR): ν = 1732 (w), 1604 (w), 1575 (w), 1504 (w), 1454 (w), 1304 (m), 1144 (s), 1038 (m), 768 (w), 721 (w), 586 cm^{-1} (w). MS (ESI+): Mass calculated for $[M+H]^+$: m/z = 787.1, measured: m/z = 787.0.

*Synthesis of (R)-2-((5-Bromo-4-((2,2'-dimethyl-3'-(prop-2-yn-1-yloxy)-[1,1'-biphenyl]- 3-yl)methoxy)-2-((5-(methylsulfonyl)pyridine-3-yl)methoxy)benzyl)amino)-3-(((R)-3-(diethoxyphosphoryl)-1-((2-(diethoxyphosphoryl)ethyl)amino)-1-oxopropane-2-yl)amino)-3-oxopropan-1-sulfonic acid (**6**)*

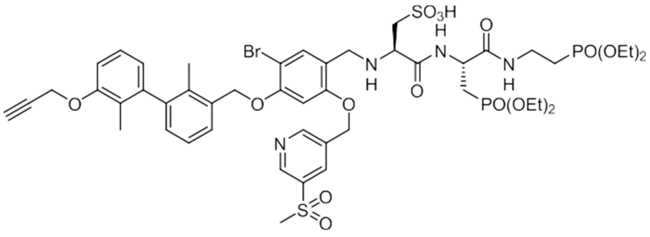

The carboxylic acid **35** (80.0 mg, 102 µmol, 1.00 eq.), the amine **13** (43.4 mg, 112 µmol, 1.10 eq.), HBTU (40.4 mg, 107 µmol, 1.05 eq.), HOBt (13.7 mg, 102 µmol, 1.00 eq.) and abs. DIPEA (26.7 µL, 203 µmol, 2.00 eq.) were reacted in abs. DMF (3 mL) according to procedure 1.1.1. Purification was performed using semi-preparative RP-HPLC (Agilent Zorbax SB C-18 5 µm 80 Å, 9.4 x 250 mm with 42–90% MeCN (0.1% TFA) in H_2O (0.1% TFA) in a linear gradient over 45 min, 6 mL/min, R_t = 10 min). Subsequent lyophilization yielded the alkyne **6** (67.0 mg, 57.9 µmol, 57%) as a colorless powder.

m.p. = 165–170 °C (decomposition). R_t = 12.50 min (system A), purity (HPLC) = 89.4%. ^{1}H-NMR (400 MHz, DMSO-d$_6$) δ = 9.37–9.39 (m, 2H), 9.13 (s, 1H), 9.08–9.09 (m, 1H), 8.88 (bs, 1H), 8.53 (s, 1H), 8.34 (t, ^3J = 5.3 Hz, 1H), 7.80 (s, 1H), 7.50–7.52 (m, 1H), 7.20–7.29 (m, 3H), 7.04–7.10 (m, 2H), 6.74 (d, ^3J = 7.5 Hz, 1H), 5.41–5.51 (m, 2H), 5.27–5.36 (m, 2H), 4.85–4.86 (m, 2H), 4.57–4.65 (m, 1H), 4.28–4.31 (m, 1H), 3.95–4.09 (m, 9H), 3.58 (s, 1H), 3.36 (s, 3H), 3.14–3.25 (m, 3H), 2.90–2.95 (m, 1H), 2.42–2.50 (m, 1H), 2.04–2.13 (m, 4H), 1.88–1.96 (m, 2H), 1.83 (s, 3H), 1.19–1.25 ppm (m, 12H). ^{13}C-NMR (101 MHz, DMSO-d$_6$) δ = 169.2, 169.1, 166.2, 157.1, 156.4, 156.4, 155.4, 153.5, 147.2, 142.3, 141.5, 136.9, 134.7, 134.6, 134.6, 134.5, 132.7, 129.3, 127.8, 126.2, 125.5, 124.1, 122.0, 113.7, 110.9, 101.7, 99.9, 79.5, 78.1, 69.7, 67.3, 61.1–61.6, (m), 56.9, 55.8, 50.0, 47.9, 43.7, 43.6, 33.5, 26.9 (d, ^2J = 142.8 Hz), 25.1 (d, ^2J = 136.0 Hz), 16.2–16.3 (m), 15.4, 12.8 ppm. ^{31}P-NMR (162 MHz, DMSO-d$_6$) δ = 28.2 (s, 1H), 27.7 ppm (s, 1H). IR (ATR): ν = 2984 (w), 1673 (m), 1603 (w), 1575 (m), 1446 (w), 1307 (m), 1221 (m), 1162 (s), 1145 (s), 1093 (w), 1019 (s), 963 (m), 731 (m), 701 cm^{-1} (w). MS (ESI+): Mass calculated for $[M+H]^+$: m/z = 1157.2, measured: m/z = 1157.3.

*Synthesis of Di-tert-butyl 2,2'-(7-(5-(4-(3-azidopropyl)piperazin-1-yl)-1-(tert-butoxy)-1,5-dioxopentan-2-yl)-1,4,7-triazonane-1,4-diyl)(R)-diacetate (**5**)*

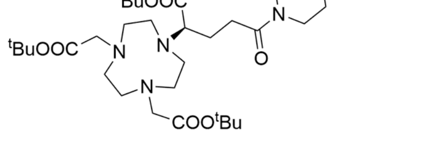

(*R*)-NODAGA(^{t}Bu)$_3$ (30.0 mg, 55.2 µmol, 1.00 equiv.), 1-(3-azidopropyl)piperazine (**31**) (14.0 mg, 82.8 µmol, 1.50 equiv.), HOBt (7.5 mg, 55.2 µmol, 1.00 eq.), HATU (25.2 mg, 66.2 µmol, 1.20 equiv.) and abs. DIPEA (14.6 µL, 110.4 µmol, 2.00 equiv.) reacted in abs. DMF (1 mL) according to procedure 1.1.1. The solvent was removed under vacuum and the crude product purified by semi-preparative RP-HPLC (Agilent Zorbax SB C-18 5 µm 80 Å, 9.4 x 250 mm with 30–80% acetonitrile (0.1% TFA) in water (0.1% TFA) in a linear gradient over 45 min, 6 mL/min, R_t = 8 min, detection at 220 nm). Subsequent lyophilization yielded linker structure **5** (36.5 mg, 52.4 µmol, 95%) as a colorless powder. R_t = 10.20 min (system A), purity = 97.0%. ^{1}H NMR (400 MHz, CD_3OD) δ = 3.97–3.98 (m, 3H), 2.59–3.66 (m), 1.91–2.13 (m, 5H), 1.45–1.50 ppm (m, 27H). ^{13}C NMR (101 MHz, CD_3OD) δ = 173.0, 171.2, 168.7, 162.7, 162.4, 119.4, 116.5, 84.5, 83.4, 64.7, 56.3, 56.3, 55.7, 52.9, 52.7, 51.6, 51.1, 47.0, 46.3, 43.5, 39.7, 31.0, 28.5, 28.5, 28.4, 26.7, 24.7 ppm. IR (ATR): $\tilde{\nu}$ = 2102 (m), 1727 (m), 1683 (s), 1456 (w), 1139 (m), 1196 (m), 1149 (s), 1127 (s), 976 (w), 830 (w), 735 (m), 719 cm^{-1} (m). MS (ESI$^+$): Mass calculated for $[M+H]^+$: m/z = 695.5, measured: m/z = 695.5.

*Synthesis of (R)-2-((4-((3'-((1-(3-(4-((R)-4-(4,7-bis(2-(tert-Butoxy)-2-oxoethyl)-1,4,7-triazonane-1-yl)-5-(tert-butoxy)-5-oxopentanoyl)piperazine-1-yl)propyl)-1H-1,2,3-triazol-4-yl)methoxy)-2,2'-di-methyl-[1,1'-biphenyl]-3-yl)methoxy)-5-bromo-2-((5-(me-thylsulfonyl)pyridine-3-yl)methoxy)-benzyl)amino)-3-(((R)-3-(diethoxyphosphoryl)-1-((2-(diethoxyphosphoryl)ethyl)amino)-1-oxopropane-2-yl)amino)-3-oxopropane-1-sulfonic acid (**37**)*

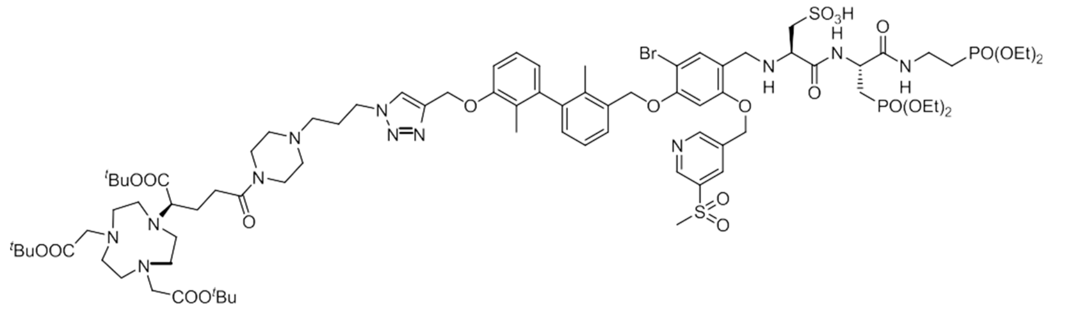

The alkyne **6** (20.0 mg, 17.5 µmol, 1.00 eq.) was reacted with the azide **5** (12.8 mg, 18.4 µmol, 1.05 eq.) in a mixture of $CuSO_4$ (0.4 mg, 2.6 µmol, 0.15 eq.), THPTA (0.8 mg, 1.7 µmol, 0.10 eq.), and sodium ascorbate (17.3 mg, 87.5 µmol, 5.00 eq.) in a 1:1 mixture of H_2O/^{t}BuOH (2 mL) at room temperature for 16 hours. The reaction solution was purified by semi-preparative RP-HPLC (Agilent Zorbax SB C-18 5 µm 80 Å, 9.4 x 250 mm with a linear gradient of 37–90% MeCN (0.1% TFA) in H_2O (0.1% TFA) over 45 min, 6 mL/min, R_t = 11 min) followed by lyophilization to yield the NODA-GA-tris(^{t}Bu)$_3$ conjugate **37** (13.0 mg, 7.1 µmol, 40%) as a colorless powder.

R_t = 12.20 min (system A), purity (HPLC) = 91,5%. IR (ATR): ν = 1667 (m), 1574 (w), 1406 (m), 1306

(m), 1199 (s), 1174 (s), 1145 (s), 1041 (m), 799 (w), 720 cm^{-1} (w). MS (ESI+): Mass calculated for $[M/2+H]^+$: *m/z* = 926.4, measured: *m/z* = 926.5.

*Synthesis of 2,2′-(7-((R)-4-(4-(3-(4-(((3′-((2-Bromo-5-((5-(methylsulfonyl)pyridin-3-yl)-methoxy)-4-((((R)-1-oxo-1-(((R)-1-oxo-3-phosphono-1-((2-phosphonoethyl)amino)- propane-2-yl)amino)-3-sulfopropane-2-yl)amino)methyl)phenoxy)methyl)-2,2′-dimethyl-[1,1′-biphenyl]-3-yl)oxy)methyl)-1H-1,2,3-triazol-1-yl)propyl)piperazine-1-yl)-1-carboxy-4-oxobutyl)-1,4,7-triazonane-1,4-diyl)diacetic acid (**1**)*

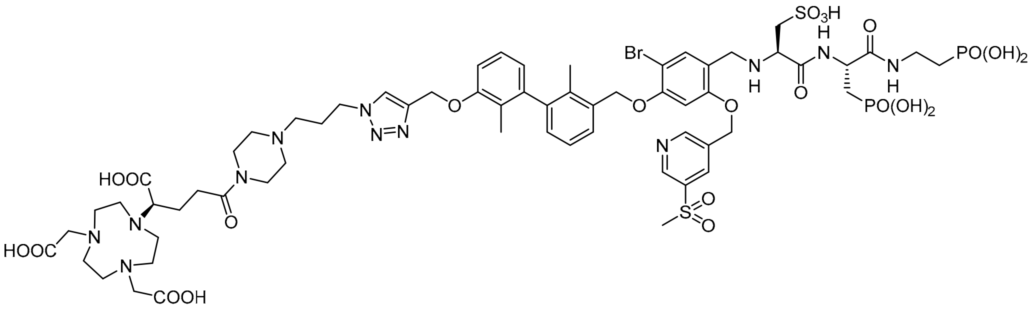

The dealkylation of the phosphonic acid esters and subsequent *tert*-butyl deprotection of the carboxylic acid esters of the NODA-GA-tris(^{t}Bu)$_3$ conjugate **37** (13.0 mg, 7.1 µmol, 1.00 eq.) was carried out using TMSBr (18.7 µL, 142 µmol, 20.0 eq.) and 500 µL of the deprotection cocktail according to procedure 1.1.4. Purification was performed by semi-preparative RP-HPLC (system C, R_t = 8 min) followed by lyophilization, which provided the NODA-GA conjugate **1** (6.5 mg, 4.2 µmol, 59% yield over two steps) as a colorless powder.

R_t = 15.87 min (system B), purity = 99.9%. IR (ATR): v = 1682 (m), 1304 (m), 1144 (s), 1037 (m), 799 (w), 751 cm^{-1} (w). MS (ESI+): Mass calculated for $[M/2+H]^+$: *m/z* = 787.20, measured: *m/z* = 787.06.

*Synthesis of 5-Iodo-4-((2,2′-dimethyl-3′-(prop-2-yn-1-yloxy)-[1,1′-biphenyl]-3-yl)methoxy)-2-hydroxybenzaldehyde (**33**)*

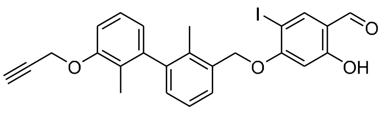

Compound **15** (100 mg, 376 µmol, 1.00 eq.), phenol **32** (119 mg, 451 µmol, 1.20 eq.), and triphenylphosphine (118 mg, 451 µmol, 1.20 eq.) were dissolved in anhydrous DMF (2 mL) and reacted with DMEAD (105 mg, 451 µmol, 1.20 eq.) in abs. DMF at 0 °C for 30 min. The reaction mixture was then warmed to room temperature and stirred for additional 16 h. The solvent was removed and column chromatographic purification on SiO_2 (PE:EtOAc, 9:1, R_f = 0.21) performed, resulting in compound **33** (108 mg, 211 µmol, 56%) as a colorless solid.

m.p. = 165–168°C °C. ^{1}H-NMR (400 MHz, $CDCl_3$) δ = 11.45 (s, 1H), 9.69 (s, 1H), 7.92 (s, 1H), 7.53 (d, ^{3}J = 7.6 Hz, 1H), 7.26–7.30 (m, 1H), 7.21 (t, ^{3}J = 7.9 Hz, 1H), 7.15 (d, ^{3}J = 7.6 Hz, 1H), 6.99 (d, ^{3}J = 8.2 Hz, 1H), 6.81 (d, ^{3}J = 7.6 Hz, 1H), 6.55 (s, 1H), 5.20 (s, 2H), 4.76 (s, 2H), 2.54 (s, 1H), 2.07 (s, 3H), 1.95 ppm (s, 3H). ^{13}C-NMR (101 MHz, $CDCl_3$) δ = 193.7, 164.7, 163.7, 156.0, 144.0, 143.0, 142.4, 134.6, 133.4, 130.0, 127.4, 126.1, 125.8, 122.9, 117.3, 110.7, 100.8, 79.1, 75.5, 74.6, 70.6, 56.3, 15.9, 13.1 ppm. IR (ATR): ν = 3270 (m), 1651 (m), 1612 (m), 1482 (w), 1448 (m), 1365 (w), 1270 (s), 1198 (s), 1178 (m), 1075 (m), 830 (w), 799 (w), 696 (w), 652^{-1} (w). MS (ESI+): Mass calculated for $[M+H]^+$: *m/z* = 513.0, measured: *m/z* = 513.0.

*Synthesis of 4-((2,2'-Dimethyl-3'-(prop-2-yn-1-yloxy)-[1,1'-biphenyl]-3-yl)methoxy)-5-iodo-2-((5-(methylsulfonyl)pyridine-3-yl)methoxy)benzaldehyde (**12**)*

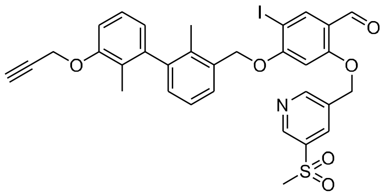

The phenol **33** (142 mg, 277 µmol, 1.00 eq) reacted with chloride **22** (80.0 mg, 319 µmol, 1.15 eq) and potassium carbonate (57.5 g, 416 µmol, 1.50 eq) in abs. DMF (3 mL) at room temperature for 16 h. The solvent was removed and column chromatography on SiO_2 (PE:EtOAc, R_f = 0.25) yielded the aldehyde **12** (178 mg, 261 µmol, 94%) as a colorless solid.

m.p. = 83–85°C ^{1}H-NMR (400 MHz, $CDCl_3$) δ = 10.20 (s, 1H), 9.18 (s, 1H), 8.99 (s, 1H), 8.40 (s, 1H), 8.29 (s, 1H), 7.51 (d, ^{3}J = 7.5 Hz, 1H), 7.22–7.29 (m, 1H), 7.16–7.25 (m, 2H), 6.99 (d, ^{3}J = 8.1 Hz, 1H), 6.80 (d, ^{3}J = 7.6 Hz, 1H), 6.60 (s, 1H), 5.25–5.28 (m, 4H), 4.76 (s, 2H), 3.17 (s, 3H), 2.54 (s, 1H), 2.11 (s, 3H), 1.94 ppm (s, 3H). ^{13}C-NMR (101 MHz, CDCl3) δ = 186.4, 163.1, 161.8, 156.0, 142.8, 142.6, 140.8, 134.8, 133.3, 130.2, 127.7, 126.2, 127.7, 126.2, 125.8, 125.6, 122.8, 120.9, 110.7, 97.6, 79.1, 78.0, 77.4, 75.5, 70.9, 67.7, 56.3, 45.0, 16.1, 13.1 ppm. IR (ATR): ν = 1668 (w), 1585 (s), 1455 (w), 1404 (w), 1308 (m), 1267 (m), 1144 (s), 1014 (m), 962 (w), 765 (w), 721^{-1} (w). MS (ESI+): Mass calculated for $[M+H]^+$: *m/z* = 682.1, measured: *m/z* = 682.0.

*Synthesis of (5-Iodo-4-((2,2'-dimethyl-3'-(prop-2-yn-1-yloxy)-[1,1'-biphenyl]-3-yl)methoxy)-2-((5-(methylsulfonyl)pyridine-3-yl)methoxy)benzyl)(sulfo)-D-alanine (**36**)*

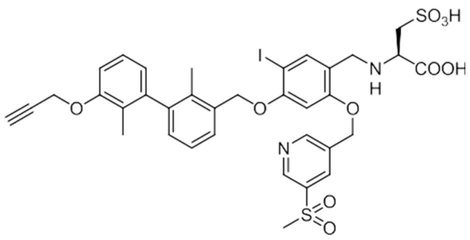

The aldehyde **12** (100 mg, 147 µmol, 1.00 eq.), L-cysteic acid (156 mg, 440 µmol, 3.00 eq.), and $NaBH_3CN$ (13.8 mg, 220 µmol, 1.50 eq.) reacted in a mixture of abs. MeOH/DMF (2 mL) according to procedure 1.1.3. After complete conversion (analytical RP-HPLC, system A), the solvent was removed under vacuum, and the residue purified by semi-preparative RP-HPLC (system D, R_t = 9 min). The resulting product was lyophilized to give the carboxylic acid **36** (53.8 mg, 64.7 µmol, 45%) as a colorless powder.

m.p. = 161–163 °C. R_t = 12,80 min (System A), purity (HPLC) = 95.1%. ^{1}H-NMR (400 MHz, DMSO-d$_6$) δ = 9.29 (bs, 1H), 9.10 (d, ^4J = 1.9 Hz, 1H), 9.07 (d, ^4J = 2.2 Hz, 1H), 8.51 (t, ^4J = 2.0 Hz, 1H), 7.81 (s, 1H), 7.53–7.55 (m, 1H), 7.24–7.29 (m, 2H), 7.05–7.12 (m, 3H), 6.74 (d, ^3J = 7.4 Hz, 1H), 5.47 (s, 2H), 5.28 (s, 2H), 4.86 (s, 2H), 4.19–4.30 (m, 3H), 3.59 (t, ^3J = 4.9 Hz, 1H), 3.36 (s, 3H), 3.03–3.07 (m, 1H), 2.87–2.93 (m, 1H), 2.05 (s, 3H), 1.84 ppm (s, 3H). ^{13}C-NMR (101 MHz, DMSO-d$_6$) δ = 168.4, 158.9, 158.0, 155.4, 153.5, 147.2, 142.3, 141.5, 141.0, 136.9, 134.7, 134.5, 134.5, 132.7, 129.2, 127.7, 126.2, 125.4, 124.1, 122.0, 114.5, 110.8, 99.1, 79.5, 78.1, 75.5, 69.8, 67.2, 56.3, 55.8, 48.6, 44.5, 43.6, 15.6, 12.8 ppm. IR (ATR): ν = 1733 (w), 1599 (w), 1574 (w), 1455 (m), 1307 (m), 1145 (s), 1037 (m), 784 (w), 767^{-1} (w). MS (HR-ESI+): Exact mass calculated for $[M+H]^+$: m/z = 835.0856, measured: m/z = 835.0846.

*Synthesis of (R)-3-(((R)-3-(Diethoxyphosphoryl)-1-((2-(diethoxyphosphoryl)ethyl)amino)-1-oxopropane-2-yl)amino)-2-((4-((2,2'-dimethyl-3'-(prop-2-yn-1-yloxy)-[1,1'-biphenyl]-3-yl)methoxy)-5-iodo-2-((5-(methylsulfonyl)pyridine-3-yl)methoxy)benzyl)amino)-3-oxopropane-1-sulfonic acid (**7**)*

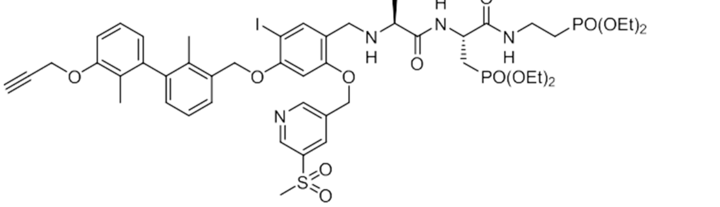

The carboxylic acid **36** (70.0 mg, 83.9 µmol, 1.00 eq.), the amine **13** (35.8 mg, 92.2 µmol, 1.10 eq.), HBTU (33.4 mg, 88.1 µmol, 1.05 eq.), HOBt (11.3 mg, 83.9 µmol, 1.00 eq.), and abs. DIPEA (22.1 µL, 168 µmol, 2.00 eq.) reacted in abs. DMF (2 mL) according to procedure 1.1.1. The product was purified using a semi-preparative RP-HPLC (system D, R_t = 12 min) and subsequently lyophilized to

yield the alkyne **7** (30.1 mg, 25.2 µmol, 30%) as a colorless powder.

m.p. = 173–175 °C (decomposition). R_t = 12.60 min (system A), purity (HPLC) = 95.2%. ^{1}H-NMR (400 MHz, DMSO-d$_6$) δ = 9.32–9.32 (m, 2H), 9.13 (s, 1H), 9.08 (s, 1H), 8.88 (bs, 1H), 8.53 (s, 1H), 8.35 (t, ^3J = 5.4 Hz, 1H), 7.93 (s, 1H), 7.54–7.56 (m, 1H), 7.22–7.28 (m, 2H), 7.05–7.11 (m, 3H), 6.74 (d, ^3J = 7.6 Hz, 1H), 5.41–5.51 (m, 2H), 5.24–5.32 (m, 2H), 4.86 (s, 2H), 4.57–4.66 (m, 1H), 4.26–4.29 (m, 1H), 3.97–4.06 (m, 8H), 3.59 (s, 1H), 3.26 (s, 3H), 3.14–3.27 (m, 3H), 2.90–2.95 (m, 1H), 2.42–2.46 (m, 1H), 2.02–2.13 (m, 4H), 1.88–1.96 (m, 2H), 1.84 (s, 3H), 1.22–1.25 ppm (m, 12H). ^{13}C-NMR (101 MHz, DMSO-d$_6$) δ = 169.2, 169.1, 166.2, 158.9, 158.8, 158.1, 155.4, 153.5, 147.2, 142.3, 141.5, 136.9, 134.6, 134.5, 134.5, 132.7, 129.2, 127.8, 126.2, 125.4, 124.1, 122.0, 114.5, 110.8, 98.9, 79.5, 78.1, 75.4, 75.4, 69.8, 6.72, 61.1–61.6, (m), 57.0, 55.8, 50.0, 47.9, 43.6, 33.5, 26.9 (d, ^2J = 142.9 Hz), 25.1 (d, ^2J = 135.8 Hz), 16.2–16.3 (m), 15.6, 12.8 ppm. ^{31}P-NMR (162 MHz, DMSO-d$_6$) δ = 28.2 (s, 1H), 27.7 ppm (s, 1H). IR (ATR): v = 1673 (m), 1598 (w), 1573 (m), 1497 (w), 1446 (m), 1403 (w), 1308 (m), 1202 (m), 1146 (s), 1093 (w), 1022 (s), 965 (m), 786 cm^{-1} (w). MS (ESI+): Mass calculated for [M+H]$^+$: m/z = 1205.23, measured: m/z = 1205.30.

*Synthesis of (R)-2-((4-((3'-((1-(3-(4-((R)-4-(4,7-Bis(2-(tert-butoxy)-2-oxoethyl)-1,4,7- triazonane-1-yl)-5-(tert-butoxy)-5-oxopentanoyl)piperazine-1-yl)propyl)-1H-1,2,3-triazol-4-yl)methoxy)-2,2'-dimethyl-[1,1'-biphenyl]-3-yl)methoxy)-5-iodo-2-((5-(methyl- sulfonyl)pyridin-3-yl)methoxy)-benzyl)amino)-3-(((R)-3-(diethoxyphosphoryl)-1-((2-(diethoxyphosphoryl)ethyl)amino)-1-oxopropane-2-yl)amino)-3-oxopropane-1-sulonic acid (**38**)*

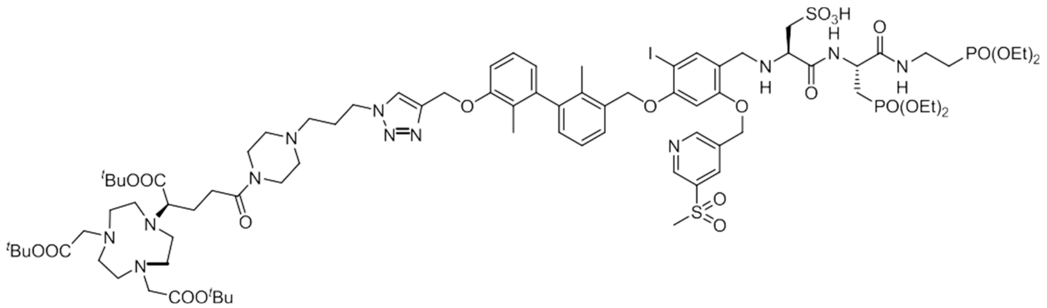

The alkyne **7** (18.0 mg, 15.7 µmol, 1.00 eq.) was reacted with the azide **5** (12.0 mg, 17.2 µmol, 1.10 eq.) in a mixture of $CuSO_4$ (0.3 mg, 1.6 µmol, 0.10 eq.), THPTA (1.4 mg, 3.1 µmol, 0.20 eq.), and sodium ascorbate (3.1 mg, 15.7 µmol, 1.00 eq.) in a degassed H_2O/^{t}BuOH mixture (1:1, 1 mL) at room temperature for 16 h. The purification by semi-preparative RP-HPLC [Agilent Zorbax SB C-18 5 µm 80 Å, 9.4 x 250 mm with a linear gradient of 42–90% MeCN (0.1% TFA) in H_2O (0.1% TFA) over 45 min, 6 mL/min, R_t = 12 min] followed by lyophilization provided the NODA-GA-tris(^{t}Bu)$_3$ conjugate **38** (21.9 mg, 11.4 µmol, 73%) as a colorless powder.

R_t = 12.00 min (system A), purity (HPLC) = 99,9%. IR (ATR): v = 1661 (m), 1604 (w), 1574 (w), 1445

(w), 1306 (m), 1232 (m), 1163 (m), 1145 (s), 1094 (w), 1021 (s), 964 (m), 786 (w), 767 cm^{-1} (w). MS (ESI+): Mass calculated for $[M/2+H]^{+}$: *m/z* = 950.4, measured: *m/z* = 950.4.

*Synthesis of 2,2'-(7-((R)-1-Carboxy-4-(4-(3-(4-(((3'-((2-iodo-5-((5-(methylsulfonyl)-pyridine-3-yl)methoxy)-4-((((R)-1-oxo-1-(((R)-1-oxo-3-phosphono-1-((2-phosphono-ethyl)amino)propane-2-yl)amino)-3-sulfopropane-2-yl)amino)methyl)phenoxy)methyl)-2,2'-dimethyl-[1,1'-biphenyl]-3-yl)oxy)methyl)-1H-1,2,3-triazol-1-yl)propyl)pi- perazin-1-yl)-4-oxobutyl)-1,4,7-triazonan-1,4-diyl)diacetic acid (**2**)*

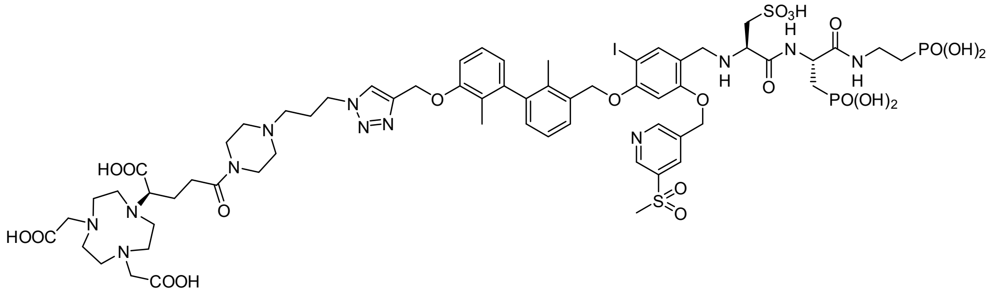

The dealkylation of the phosphonic acid ester and subsequent *tert*-butyl deprotection of the carboxylic acid esters of the NODA-GA-tris(^{t}Bu)$_3$ conjugate **38** (21.9 mg, 11.6 µmol, 1.00 eq.) was carried out using TMSBr (30.7 µL, 232 µmol, 20.0 eq.) and 500 µL of deprotection cocktail, according to procedure 1.1.4. Purification by semi-preparative RP-HPLC (system C, R_t = 9 min) was followed by lyophilization, yielding the NODA-GA conjugate **2** (11.2 mg, 6.9 µmol, 59% over two steps) as a colorless powder.

R_t = 16.08 min (system B), purity = 97,3%. IR (ATR): *v* = 1651 (m), 1574 (m), 1434 (m), 1306 (m), 1145 (s), 1040 (m), 799 (w), 720 cm^{-1} (w). MS (ESI+): Mass calculated for $[M/2+H]^{+}$: *m/z* = 810.39, measured: *m/z* = 810.19.

*Synthesis of (R)-3-(Bis(2-(diethoxyphosphoryl)ethyl)amino)-2-((5-chloro-4-((2,2'-di- methyl-3'-(prop-2-yn-1-yloxy)-[1,1'-biphenyl]-3-yl)methoxy)-2-((5-(methylsulfonyl)-pyridine-3-yl)methoxy)benzyl)-amino)-3-oxopropane-1-sulfonic acid (**8**)*

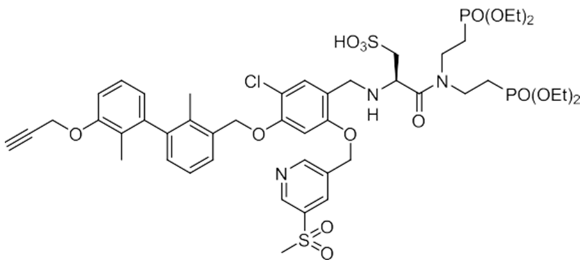

The carboxylic acid **34** (52.0 mg, 70.0 µmol, 1.00 eq.), the amine **14** (26.6 mg, 77.0 µmol, 1.10 eq.), HBTU (29.2 mg, 77.0 µmol, 1.10 eq.), HOBt (9.5 mg, 70.0 µmol, 1.00 eq.), and abs. DIPEA (24.4 µL, 139.9 µmol, 2.00 eq.) were reacted in abs. DMF (1 mL) according to procedure 1.1.1. Purification by semi-preparative RP-HPLC [Agilent Zorbax SB C-18 5 µm 80 Å, 9.4 x 250 mm with 45–90% MeCN (0.1% TFA) in H_2O (0.1% TFA) in a linear gradient over 45 min, 6 mL/min, R_t = 7 min] followed by lyophilization afforded alkyne **8** (50.4 mg, 47.1 µmol, 72%) as a colorless powder.

m.p. = 163–169 °C (decomposition). R_t = 12,50 min (system A), purity (HPLC) = 99.9%. ^{1}H-NMR (400 MHz, DMSO-d$_6$) δ = 9.39 (bs, 1H), 9.12 (d, ^4J = 1.9 Hz, 1H), 9.07 (d, ^4J = 2.2 Hz, 1H), 8.89 (bs, 1H), 8.53 (t, ^4J = 2.0 Hz, 1H), 7.54 (s, 1H), 7.47–7.48 (m, 1H), 7.22–7.27 (m, 3H), 7.04–7.10 (m, 2H), 6.74 (d, ^3J = 6.7 Hz, 1H), 5.48 (s, 2H), 5.30–5.32 (m, 2H), 4.85 (s, 2H), 4.54–4.59 (m, 1H), 4.18–4.22 (m, 1H), 3.97–4.02 (m, 9H), 3.67–3.70 (m, 1H), 3.58 (t, ^4J = 2.4 Hz, 1H), 3.47–3.51 (m, 2H), 3.37 (s, 3H), 3.24–3.28 (m, 1H), 3.06–3.09 (m, 1H), 2.72–2.79 (m, 1H), 2.10–2.22 (m, 3H), 1.97–2.03 (m, 4H), 1.83 (s, 3H), 1.19–1.26 ppm (m, 12H). ^{13}C-NMR (101 MHz, DMSO-d$_6$) δ = 165.5, 158.5, 158.1, 156.4, 155.5, 155.4, 153.5, 147.2, 142.3, 141.5, 136.9, 134.8, 134.6, 134.6, 134.4, 132.6, 132.2, 129.3, 127.8, 126.2, 125.5, 124.1, 122.0, 116.6, 113.8, 113.1, 113.0, 110.9, 100.2, 79.5, 78.1, 69.7, 67.4, 61.1–61.4, 55.8, 55.1, 49.3, 44.4, 43.6, 41.2, 23.7 (d, ^2J = 131.7 Hz), 22.5 (d, ^2J = 139.8 Hz), 16.1–16.3 (m), 15.3, 12.8 ppm. ^{31}P-NMR (162 MHz, DMSO-d$_6$) δ = 32.9 (s, 1H), 32.4 ppm (s, 1H). IR (ATR): v = 1161 (m), 1606 (w), 1576 (m), 1445 (m), 1307 (m), 1232 (m), 1201 (m), 1166 (s), 1146 (s), 1093 (w), 1021 (s), 964 (m), 786 (w), 720 cm^{-1} (w). MS (ESI+): Mass calculated for [M+H]$^+$: m/z = 1072.3, measured: m/z = 1072.3.

*Synthesis of (R)-2-((4-((3'-((1-(3-(4-((R)-4-(4,7-Bis(2-(tert-butoxy)-2-oxoethyl)-1,4,7- triazonane-1-yl)-5-(tert-butoxy)-5-oxopentanoyl)piperazin-1-yl)propyl)-1H-1,2,3-triazol-4-yl)methoxy)-2,2'-dimethyl-[1,1'-biphenyl]-3-yl)methoxy)-5-chlor-2-((5-(methylsulfonyl)pyridine-3-yl)methoxy)benzyl)amino)-3-(bis(2-(diethoxyphosphoryl)-ethyl)amino)-3-oxopropane-1-sulfonic acid (**39**)*

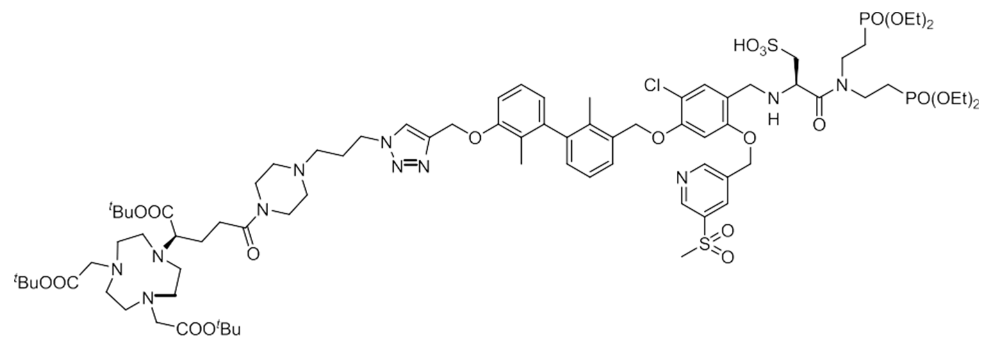

The alkyne **8** (20.0 mg, 18.7 µmol, 1.00 eq.) was reacted with the azide **5** (14.3 mg, 20.6 µmol, 1.10 eq.) in a mixture of $CuSO_4$ (0.3 mg, 1.9 µmol, 0.10 eq.), THPTA (1.6 mg, 3.7 µmol, 0.20 eq.) and sodium ascorbate (3.7 mg, 18.7 µmol, 1.00 eq.) in a 1:1 mixture of

H_2O/^{t}BuOH (1 mL) at room temperature for 16 hours. The purification was performed with semi-preparative RP-HPLC (system D, R_t = 8 min) with subsequent lyophilization. The NODA-GA-tris(^{t}Bu)$_3$ conjugate **39** (19.9 mg, 11.3 µmol, 61%) was obtained as a colorless powder.
R_t = 12.10 min (system A), purity (HPLC) = 99.9%. MS (ESI+): Mass calculated for $[M+H]^+$: *m/z* = 882.9, measured: *m/z* = 883.0.

*Synthesis of 2,2′-(7-((R)-4-(4-(3-(4-(((3′-((4-((((R)-1-(Bis(2-phosphonoethyl)amino)-1- oxo-3-sulfopropane-2-yl)amino)methyl)-2-chloro-5-((5-(methylsulfonyl)pyridin-3-yl)-ethoxy)phenoxy)-methyl)-2,2′-dimethyl-[1,1′-biphenyl]-3-yl)oxy)methyl)-1H-1,2,3-triazol-1-yl)propyl)piperazine-1-yl)-1-carboxy-4-oxobutyl)-1,4,7-triazonane-1,4-diyl)diacetic acid (**3**)*

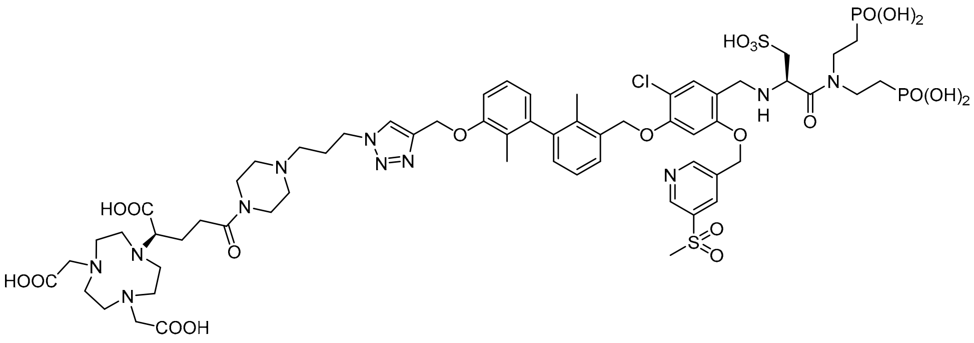

The dealkylation of the phosphonic acid ester and subsequent *tert*-butyl deprotection of the carboxylic acid ester of the NODA-GA-tris(^{t}Bu)$_3$ conjugate **39** (20.0 mg, 11.3 µmol, 1.00 eq) was carried out using TMSBr (29.9 µL, 227 µmol, 20.0 eq) and 500 µL of the deprotection cocktail according to procedure 1.1.4. Purification by semi-preparative RP-HPLC (system C, R_t = 8 min) followed by lyophilization provided the NODA-GA conjugate **3** (9.6 mg, 6.5 µmol, 57% over two steps) as a colorless powder.
R_t = 15.51 min (system B), purity = 99.9%. IR (ATR): ν = 1651 (m), 1578 (w), 1455 (m), 1307 (m), 1173 (s), 1146 (s), 1037 (m), 798 (w), 720 cm^{-1} (w). MS (ESI+): Mass calculated for $[M/2+H]^+$: *m/z* = 743.46, measured: *m/z* = 742.60.

*Synthesis of (R)-3-(Bis(2-(diethoxyphosphoryl)ethyl)amino)-2-((5-brom-4-((2,2′-dimethyl-3′-(prop-2-yn-1-yloxy)-[1,1′-biphenyl]-3-yl)methoxy)-2-((5-(methylsulfonyl)-pyridin-3-yl)methoxy)benzyl-)amino)-3-oxopropane-1-sulfonic acid (**9**)*

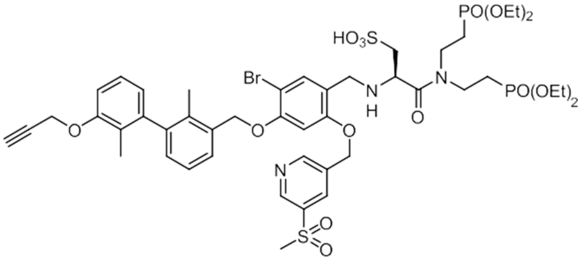

Carboxylic acid **35** (50.0 mg, 63.5 µmol, 1.00 eq), amine **14** (24.1 mg, 69.8 µmol, 1.10 eq), HBTU (26.5 mg, 69.8 µmol, 1.10 eq), HOBt (8.6 mg, 63.5 µmol, 1.00 eq), and abs. DIPEA (22.2 µL, 127.0 µmol, 2.00 eq) were reacted in abs. DMF (2 mL) according to protocol 1.1.1. The purification by semi-preparative RP-HPLC (system D, R_t = 12 min) with subsequent lyophilization provided the alkyne **9** (53.3 mg, 47.5 µmol, 75%) as a colorless powder.

m.p. = 183–185 °C (decomposition). R_t = 12.70 min (system A), purity (HPLC) = 99.9%. ^{1}H-NMR (400 MHz, DMSO-d$_6$) δ = 9.37 (bs, 1H), 9.12 (d, ^4J = 1.8 Hz, 1H), 9.07 (d, ^4J = 2.6 Hz, 1H), 8.89 (bs, 1H), 8.52 (s, 1H), 7.66 (s, 1H), 7.48–7.50 (m, 1H), 7.20–7.27 (m, 3H), 7.04–7.10 (m, 2H), 6.74 (d, ^3J = 7.6 Hz, 1H), 5.48 (s, 2H), 5.29–5.30 (m, 2H), 4.86 (s, 2H), 4.54–4.59 (m, 1H), 4.19–4.26 (m, 1H), 3.98–4.02 (m, 9H), 3.64–3.69 (m, 1H), 3.58 (t, ^4J = 2.3 Hz, 1H), 3.47–3.51 (m, 2H), 3.37 (s, 3H), 3.24–3.26 (m, 1H), 3.06–3.09 (m, 1H), 2.72–2.79 (m, 1H), 2.10–2.22 (m, 3H), 1.99–2.04 (m, 4H), 1.83 (s, 3H), 1.19–1.26 ppm (m, 11H). ^{13}C-NMR (101 MHz, DMSO-d$_6$) δ = 165.6, 158.4, 158.0, 157.0, 156.4, 155.4, 153.6, 147.2, 142.3, 141.5, 136.9, 135.1, 134.8, 134.5, 134.5, 134.4, 132.6, 129.3, 127.7, 126.2, 1255, 124.1, 122.0, 113.6, 110.9, 101.7, 100.1, 76.5, 78.1, 69.7, 67.3, 61.2–61.4 (m), 55.8, 55.1, 49.3, 44.4, 43.6, 41.2, 23.6 (d, ^2J = 141.4 Hz), 22.5 (d, ^2J = 131.3 Hz), 16.1–16.3 (m), 15.4, 12.8 ppm. ^{31}P-NMR (162 MHz, DMSO-d$_6$) δ = 28.1 (s, 1H), 27.7 ppm (s, 1H). IR (ATR): v = 1660 (m), 1574 (m), 1445 (m), 1406 (w), 1307 (m), 1232 (m), 1164 (s), 1145 (s), 1094 (w), 1021 (s), 964 (m), 786 (w), 767 cm^{-1} (w). MS (ESI+): Mass calculated for [M+H]$^+$: m/z = 1116.0, measured: m/z = 1116.3.

*Synthesis of (R)-2-((4-((3′-((1-(3-(4-((R)-4-(4,7-Bis(2-(tert-butoxy)-2-oxoethyl)-1,4,7-triazonane-1-yl)-5-(tert-butoxy)-5-oxopentanoyl)piperazine-1-yl)propyl)-1H-1,2,3-triazol-4-yl)methoxy)-2,2′-dimethyl-[1,1′-biphenyl]-3-yl)methoxy)-5-bromo-2-((5-(methylsulfonyl)pyridin-3-yl)methoxy)benzyl)-amino)-3-(bis(2-(diethoxyphosphoryl)ethyl)amino)-3-oxopropane-1-sulfonic acid (**40**)*

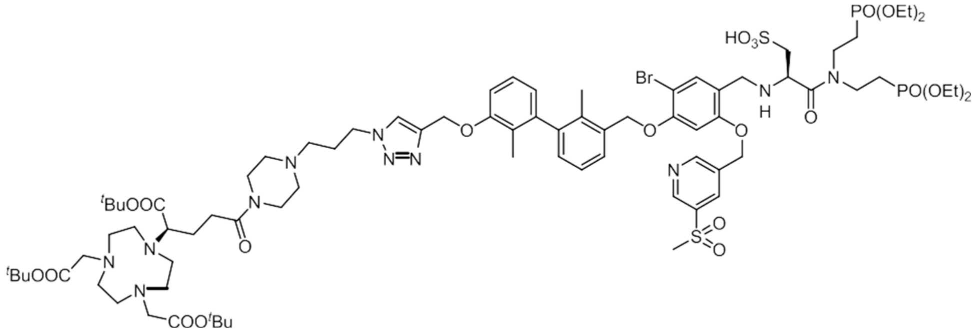

The alkyne **9** (25.0 mg, 22.4 µmol, 1.00 eq.) reacted with the azide **5** (17.1 mg, 24.7 µmol, 1.10 eq.) in a mixture of $CuSO_4$ (0.4 mg, 2.2 µmol, 0.10 eq.), THPTA (2.0 mg, 4.5 µmol, 0.20 eq.) and sodium ascorbate (4.4 mg, 22.4 µmol, 1.00 eq.) in a 1:1 mixture of H_2O/^{t}BuOH (1 mL) at room temperature for 16 h. Purification was performed by semi-preparative RP-HPLC (system D, R_t = 8 min) followed by lyophilization, yielding NODA-GA-tris(^{t}Bu)$_3$ conjugate **40** (16.2 mg, 8.8 µmol, 39%) as a colorless powder.
R_t = 12.10 min (system A), purity (HPLC) = 99.9%. MS (ESI+): Mass calculated for $[M/2+H]^+$: *m/z* = 904.9, measured: *m/z* = 905.0.

*Synthesis of 2,2'-(7-((R)-4-(4-(3-(4-(((3'-((4-((((R)-1-(Bis(2-phosphonoethyl)amino)-1-oxo-3-sulfopropane-2-yl)amino)methyl)-2-bromo-5-((5-(methylsulfonyl)pyridin-3-yl)-methoxy)phenoxy)-methyl)-2,2'-dimethyl-[1,1'-biphenyl]-3-yl)oxy)methyl)-1H-1,2,3-triazol-1-yl)propyl)piperazine-1-yl)-1-carboxy-4-oxobutyl)-1,4,7-triazonane-1,4-diyl)diacetic acid (**4**)*

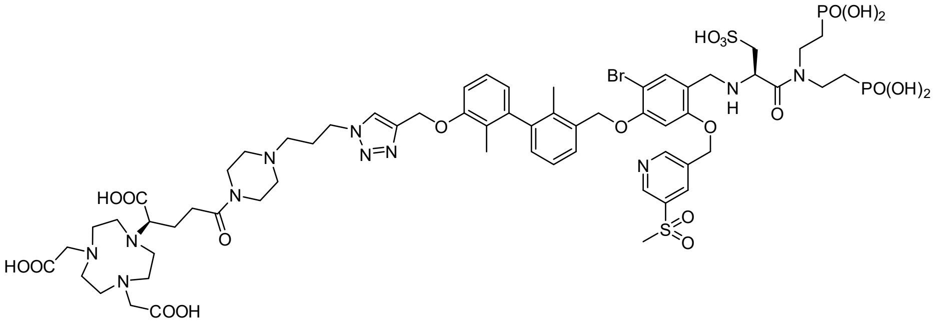

The dealkylation of the phosphonic acid ester and subsequent *tert*-butyl deprotection of the carboxylic acid ester of the NODA-GA-tris(^{t}Bu)$_3$ conjugate **40** (16.0 mg, 9.1 µmol, 1.00 eq.) was carried out with TMSBr (23.9 µL, 181 µmol, 20.0 eq.) and 500 µL of deprotection cocktail according to protocol 1.1.4. Purification by semi-preparative RP-HPLC (system C, R_t = 8 min) followed by lyophilization provided the NODA-GA conjugate **4** (7.3 mg, 4.8 µmol, 53% yield over two steps) as a colorless powder.

R_t = 15,58 min (system B), purity = 99,9%. IR (ATR): ν = 1651 (m), 1575 (w), 1445 (m), 1305 (m), 1170 (s), 1145 (s), 1038 (m), 791 (w), 720 cm^{-1} (w). MS (ESI+): Mass calculated for $[M/2+H]^+$: m/z = 765.68, measured: m/z = 765.53.

2. ^{1}H and ^{13}C NMR spectra of unknown compounds

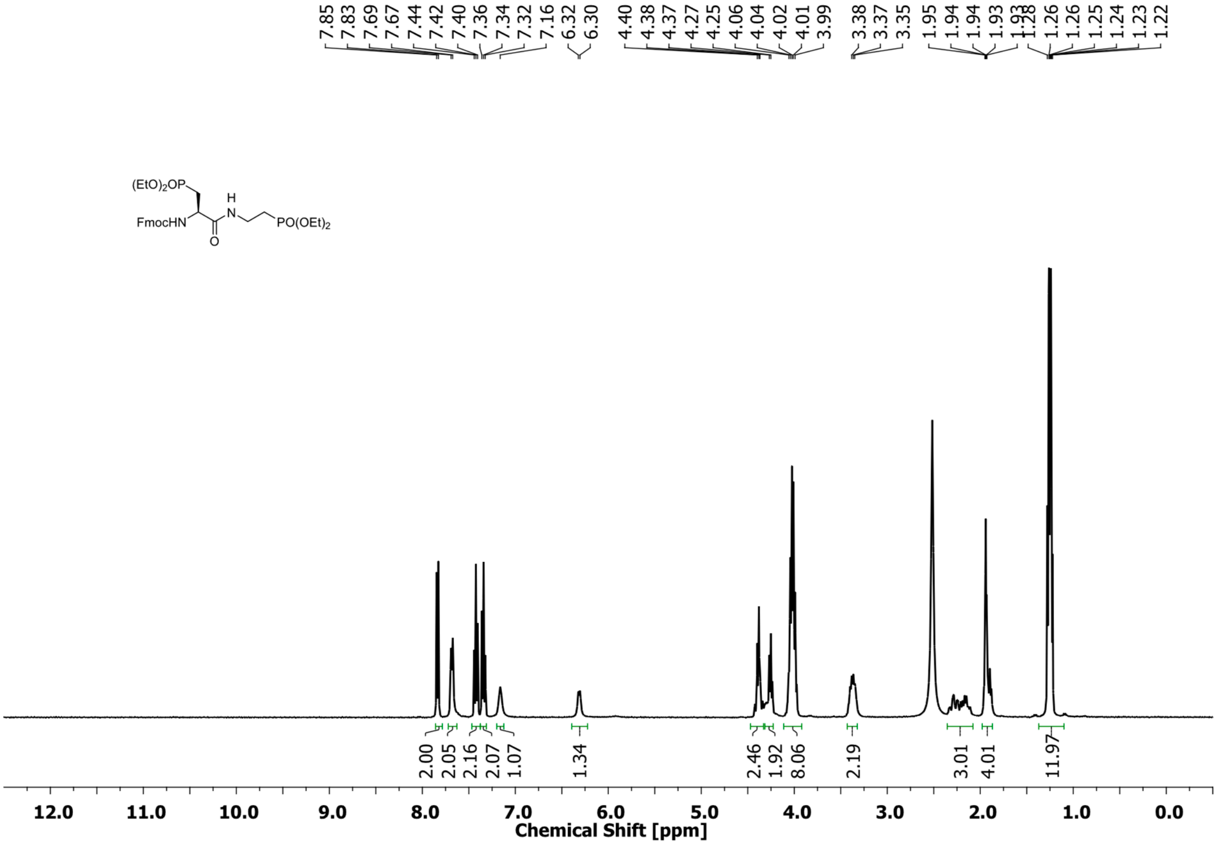

Figure S1: ^{1}H NMR spectrum (MeCN-d_3, 400 MHz, 298 K) of compound **27**.

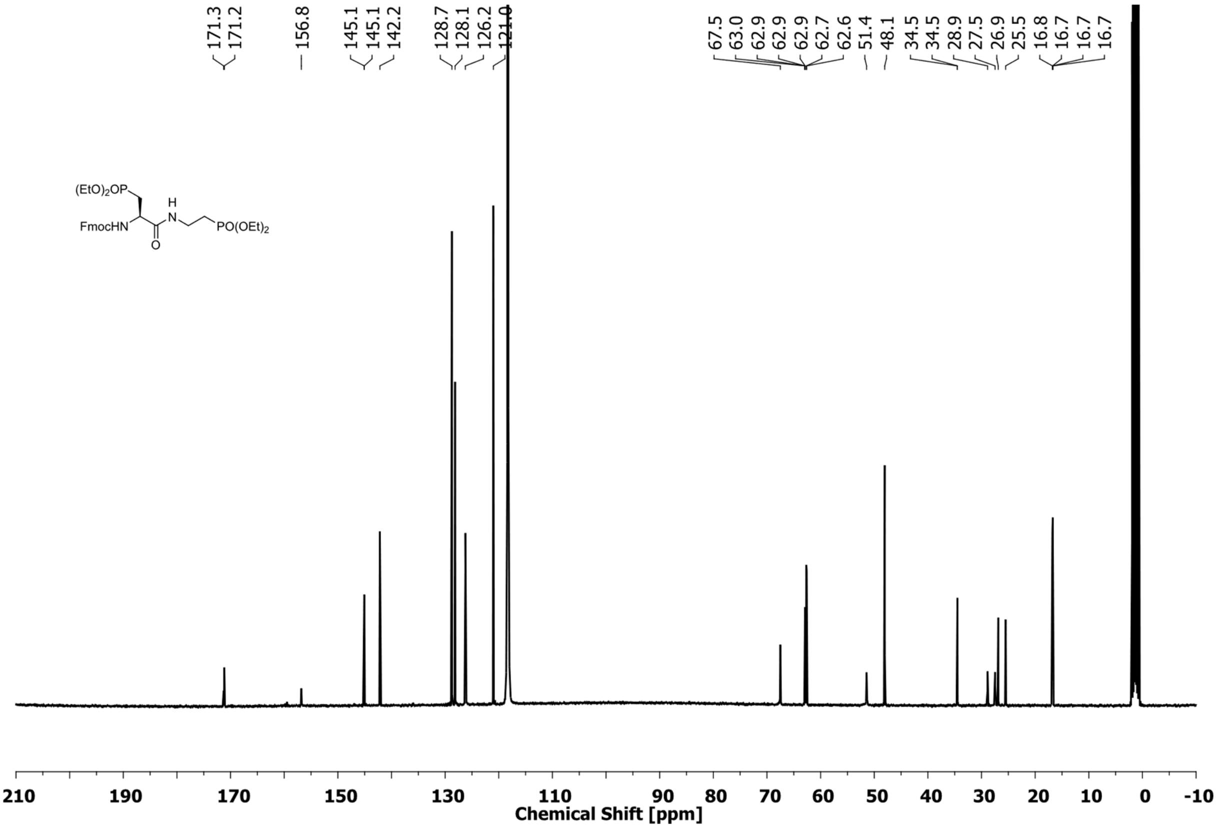

Figure S2: ^{13}C NMR spectrum (MeCN-d_3, 101 MHz, 298 K) of compound **27**.

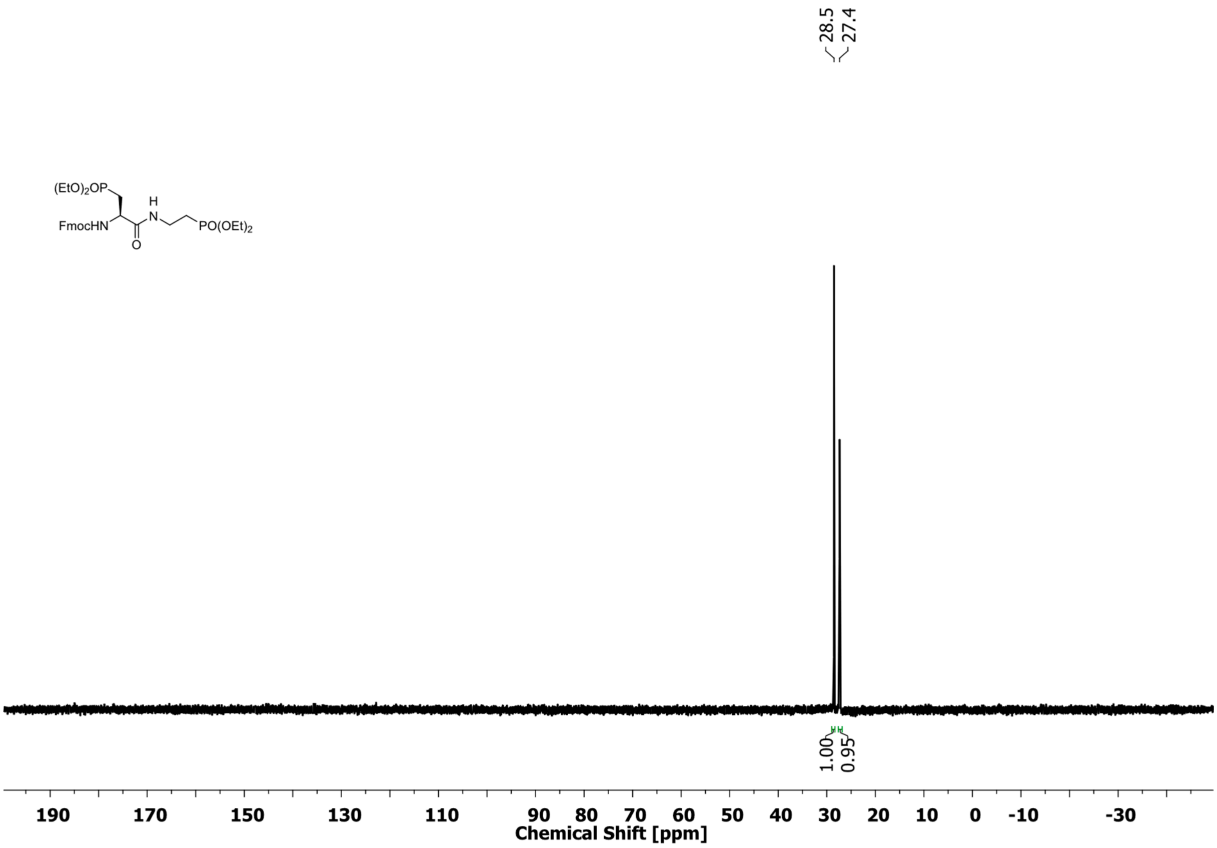

Figure S3: ^{31}P NMR spectrum (MeCN-d_3, 162 MHz, 298 K) of compound **27**.

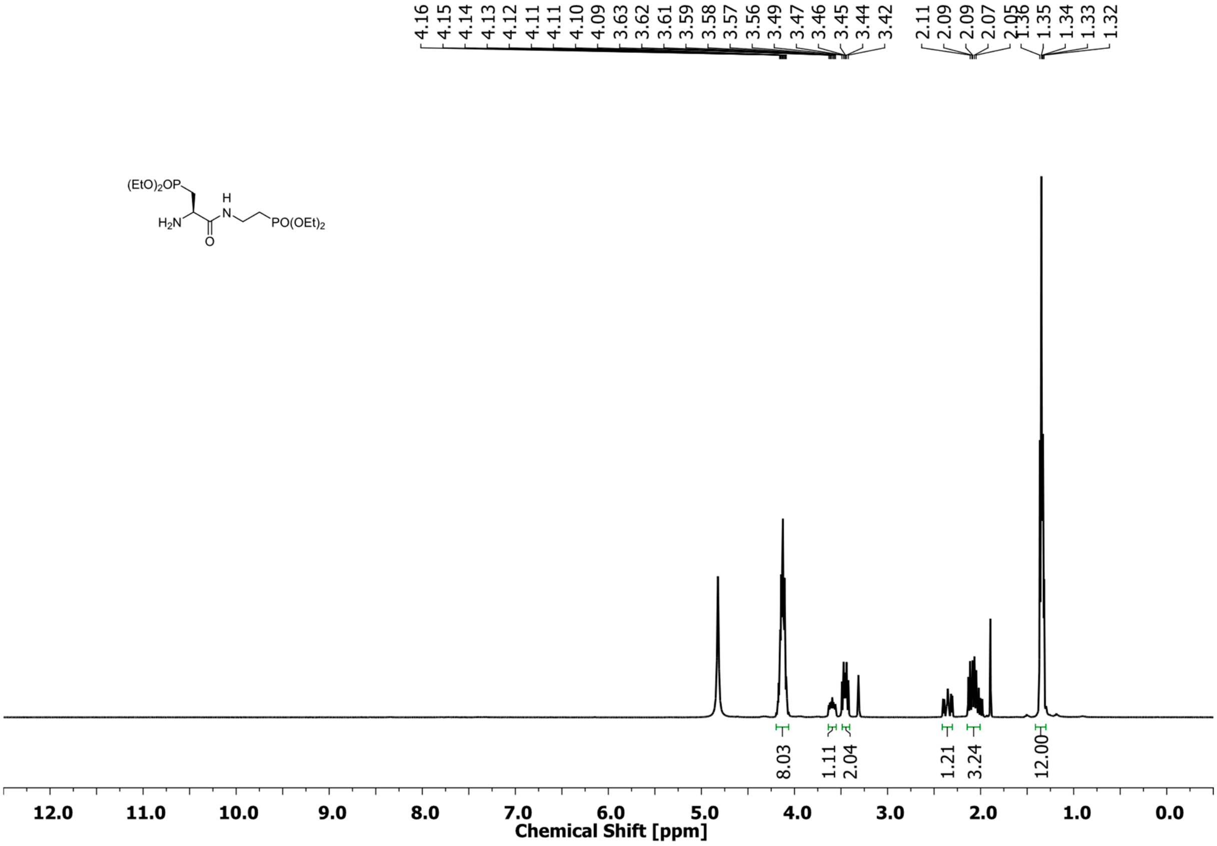

Figure S4: ^{1}H NMR spectrum (CD$_3$OD, 400 MHz, 298 K) of compound **13**.

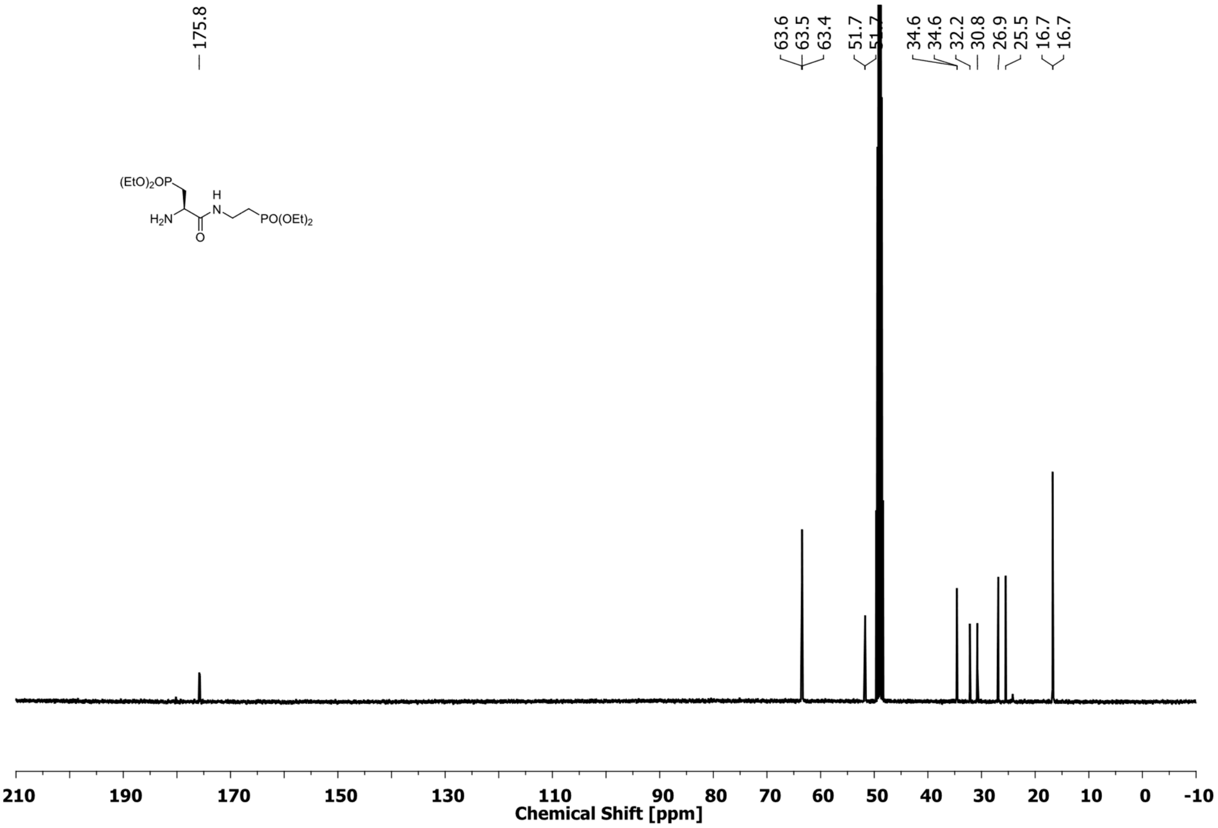

Figure S5: ^{13}C NMR spectrum (CD_3OD, 101 MHz, 298 K) of compound **13**.

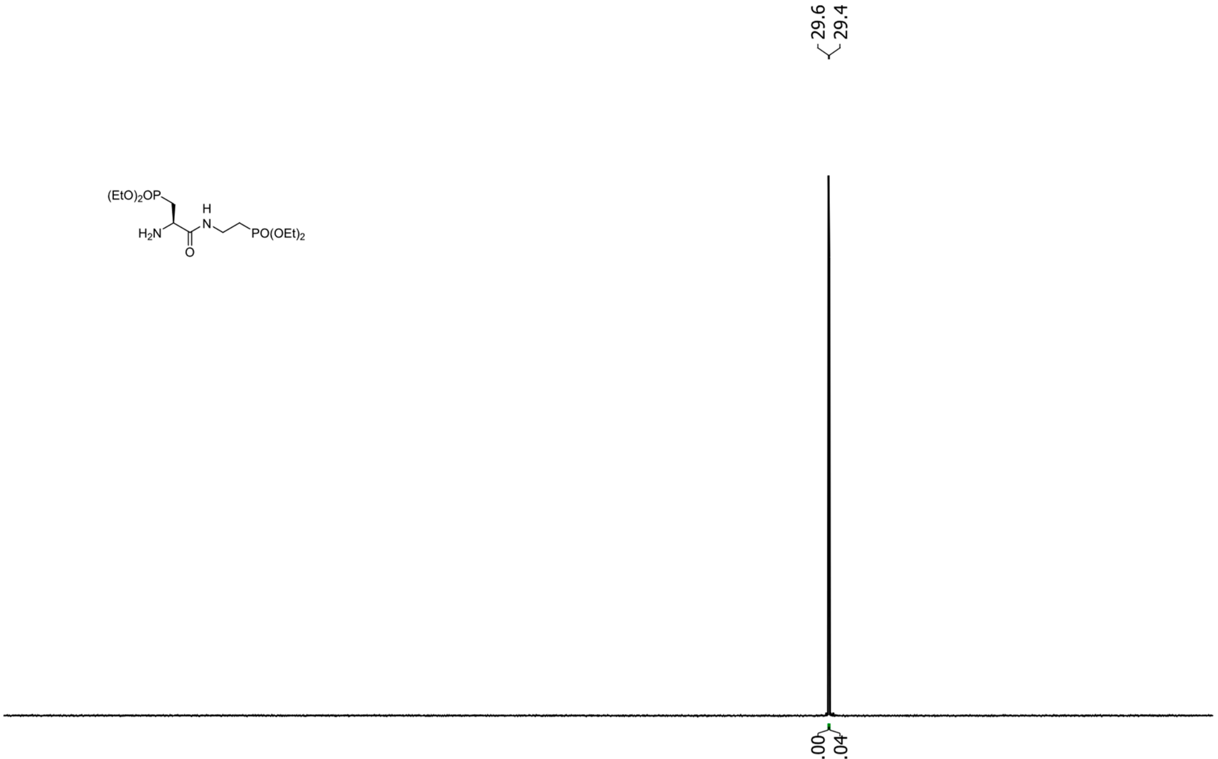

Figure S6: ^{31}P NMR spectrum (CD_3OD, 162 MHz, 298 K) of compound **13**.

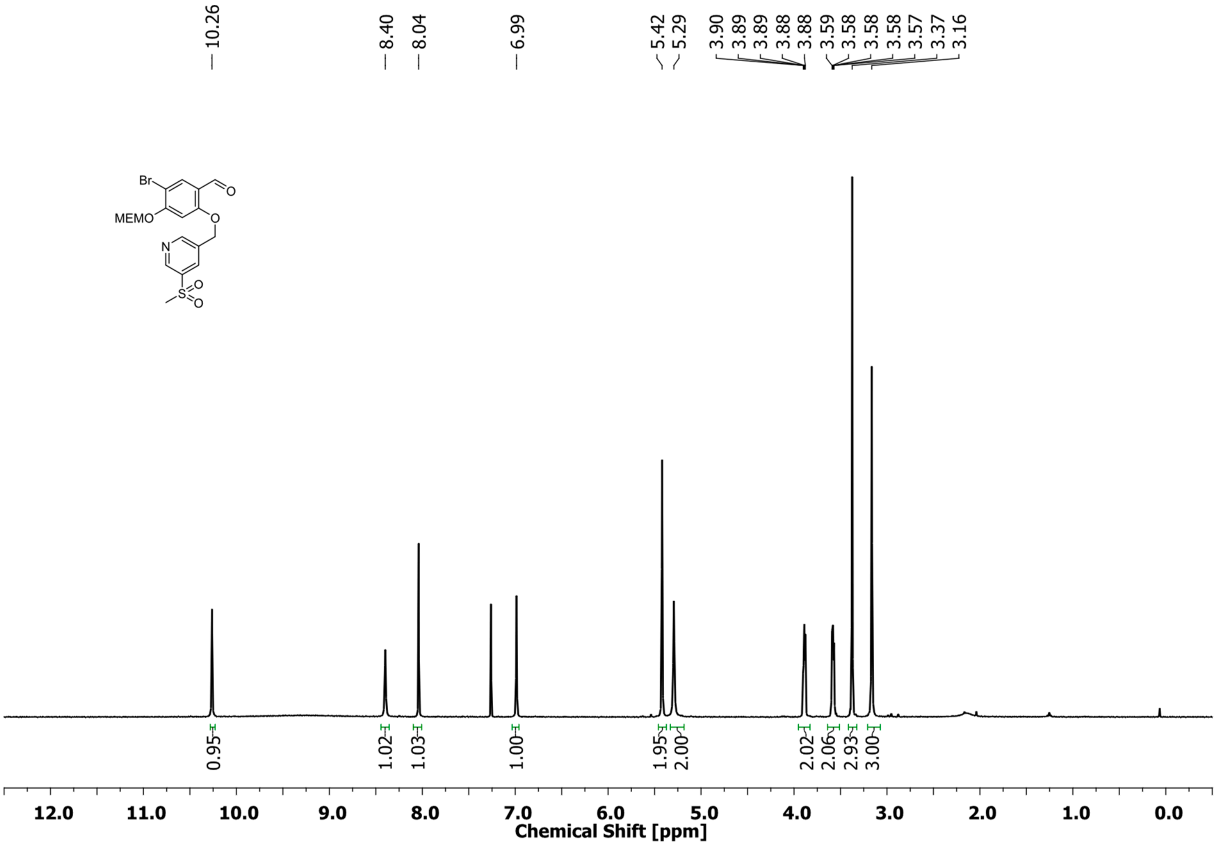

Figure S7: ^{1}H NMR spectrum ($CDCl_3$, 400 MHz, 298 K) of compound **24**.

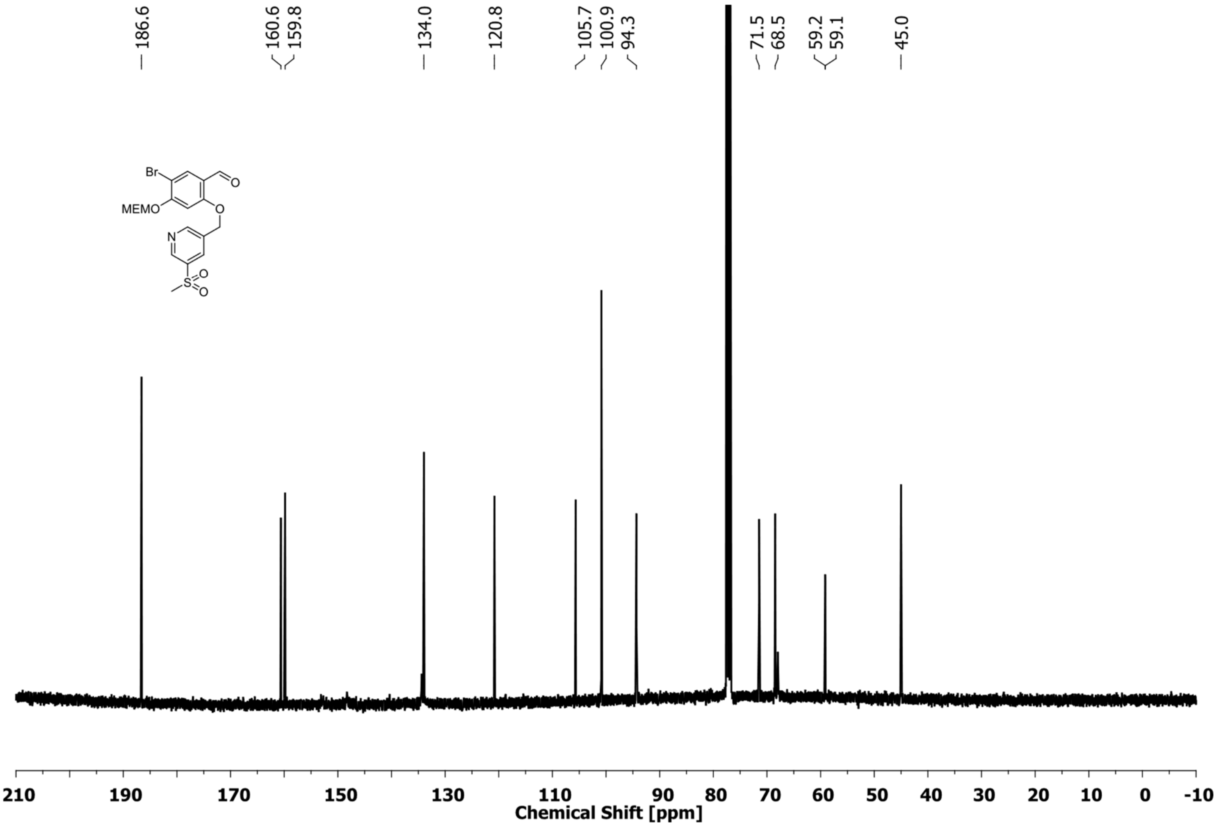

Figure S8: ^{13}C NMR spectrum ($CDCl_3$, 101 MHz, 298 K) of compound **24**.

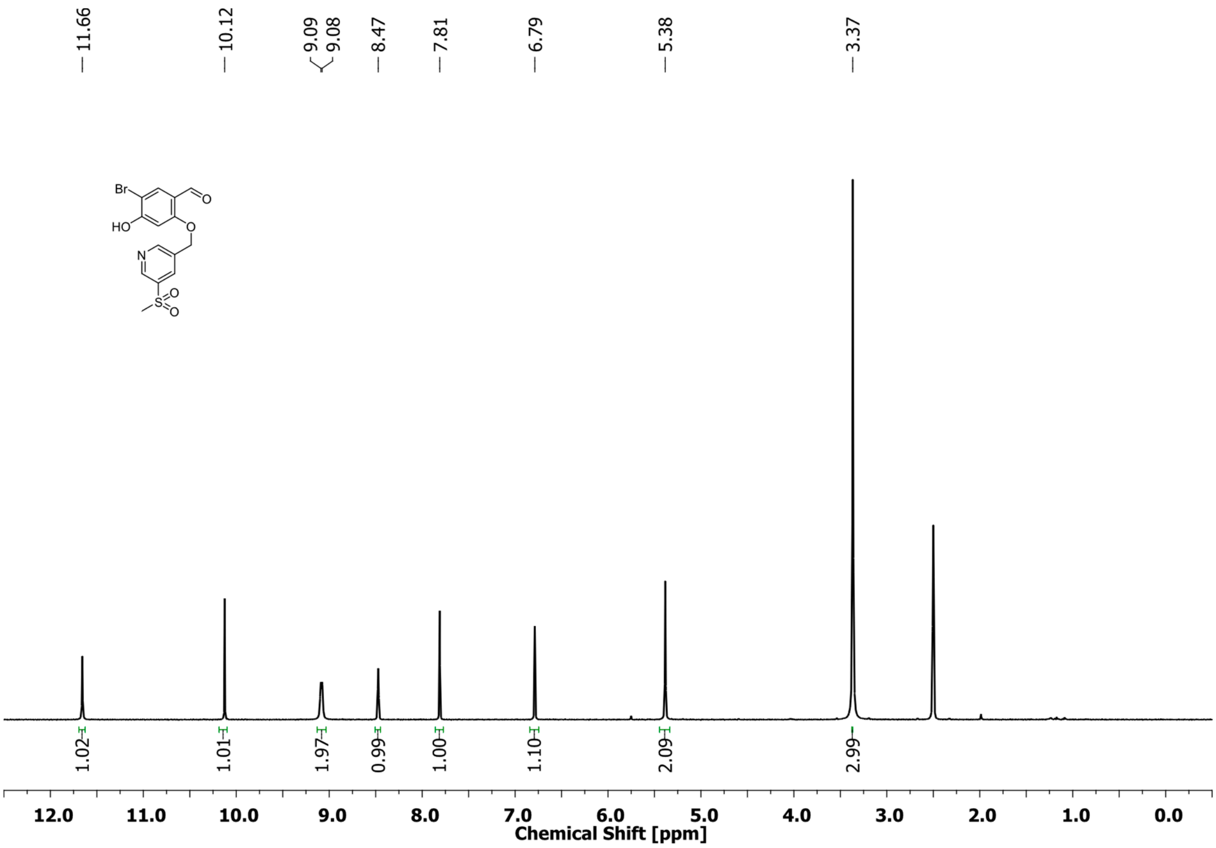

Figure S9: ^{1}H NMR spectrum (DMSO-d_6, 400 MHz, 298 K) of compound **17**.

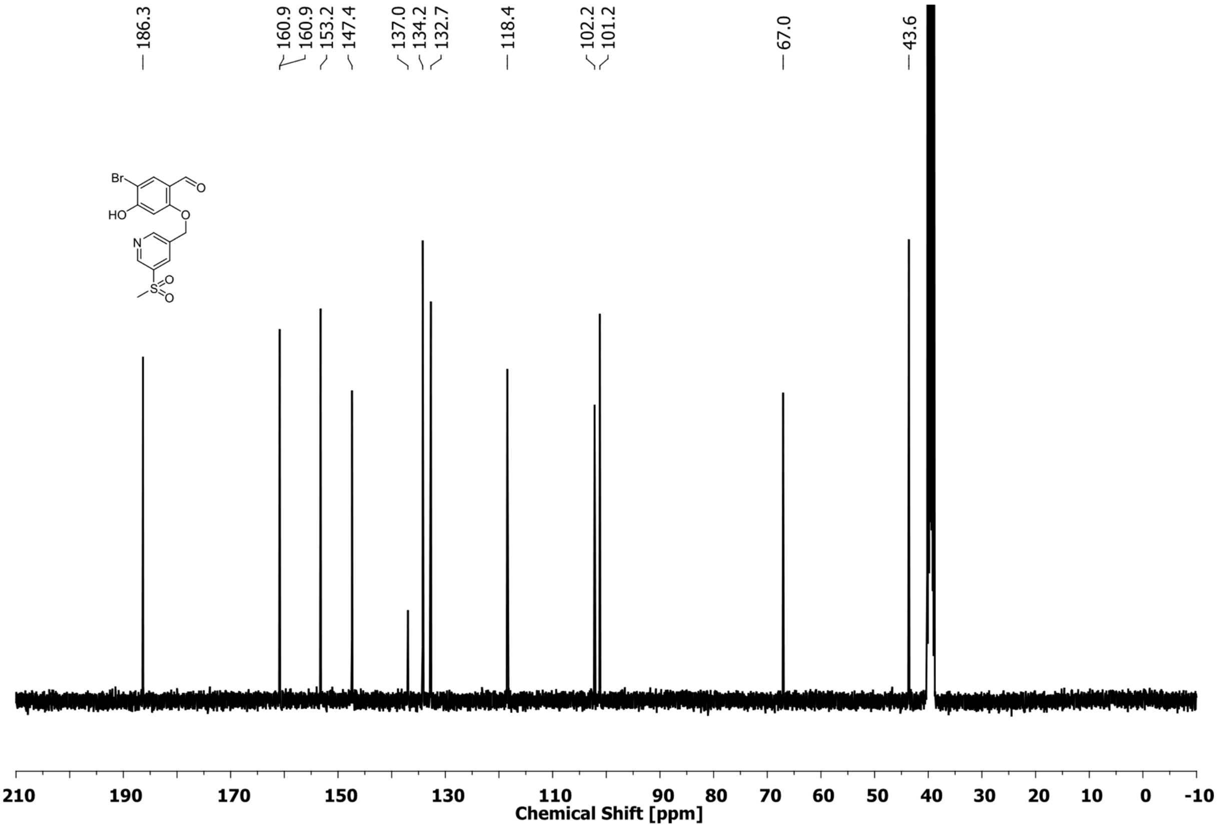

Figure S10: ^{13}C NMR spectrum (DMSO-d_6, 101 MHz, 298 K) of compound **17**.

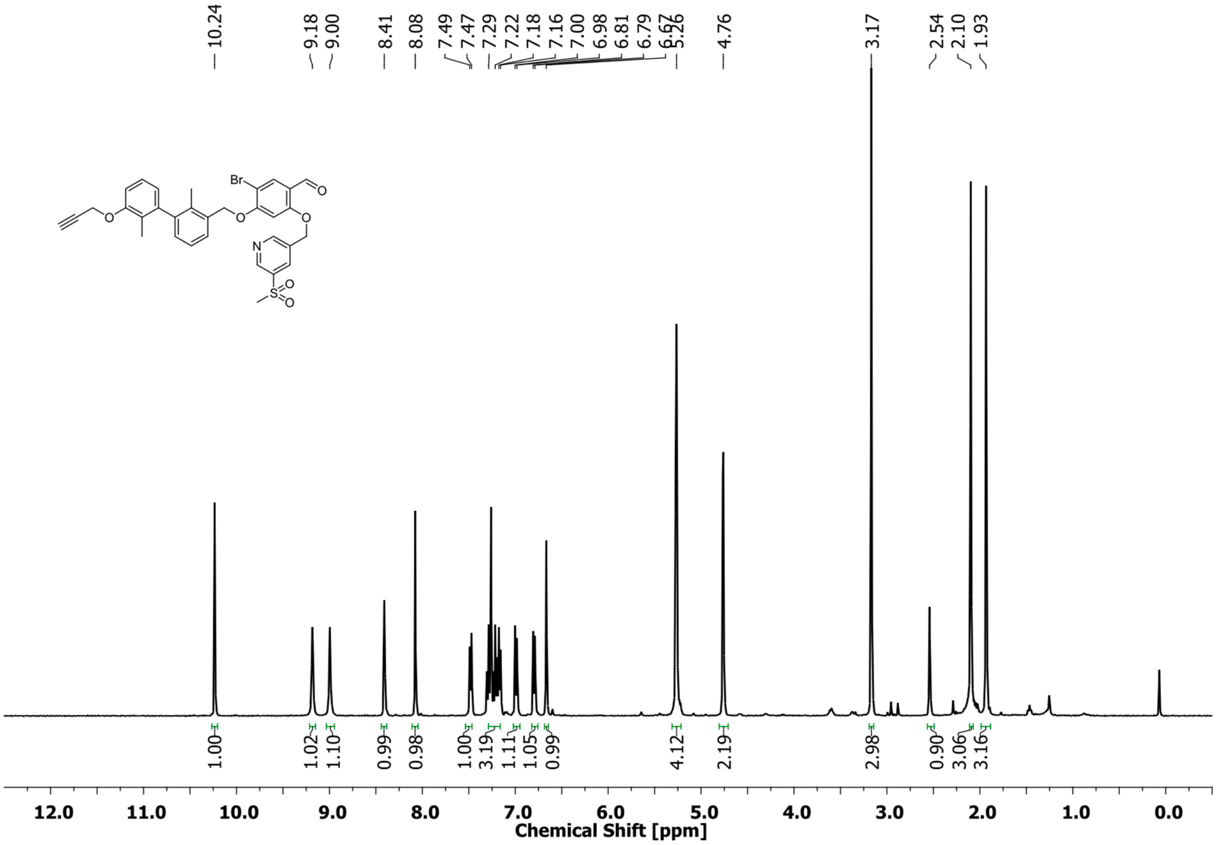

Figure S11: ^{1}H NMR spectrum ($CDCl_3$, 400 MHz, 298 K) of compound **11**.

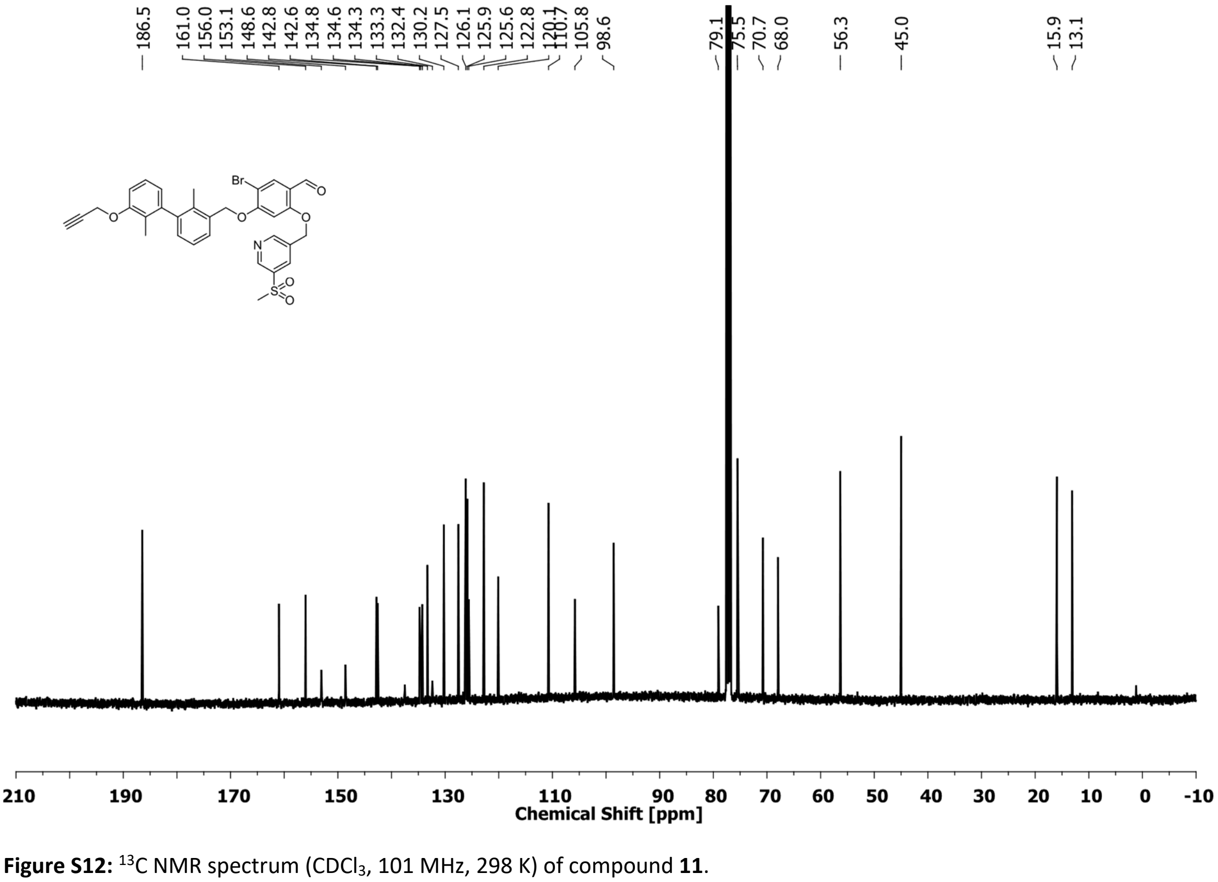

Figure S12: ^{13}C NMR spectrum ($CDCl_3$, 101 MHz, 298 K) of compound **11**.

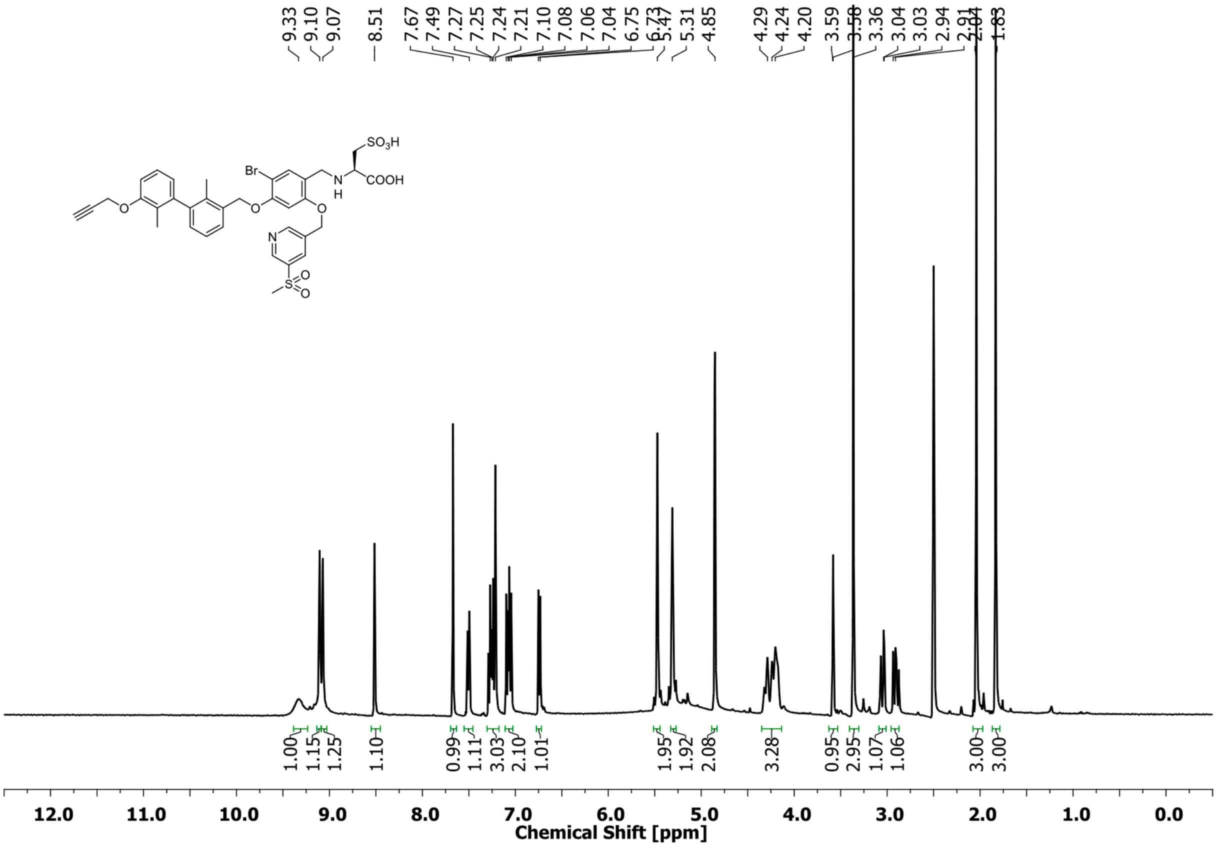

Figure S13: ^{1}H NMR spectrum (DMSO-d_6, 400 MHz, 298 K) of compound **35**.

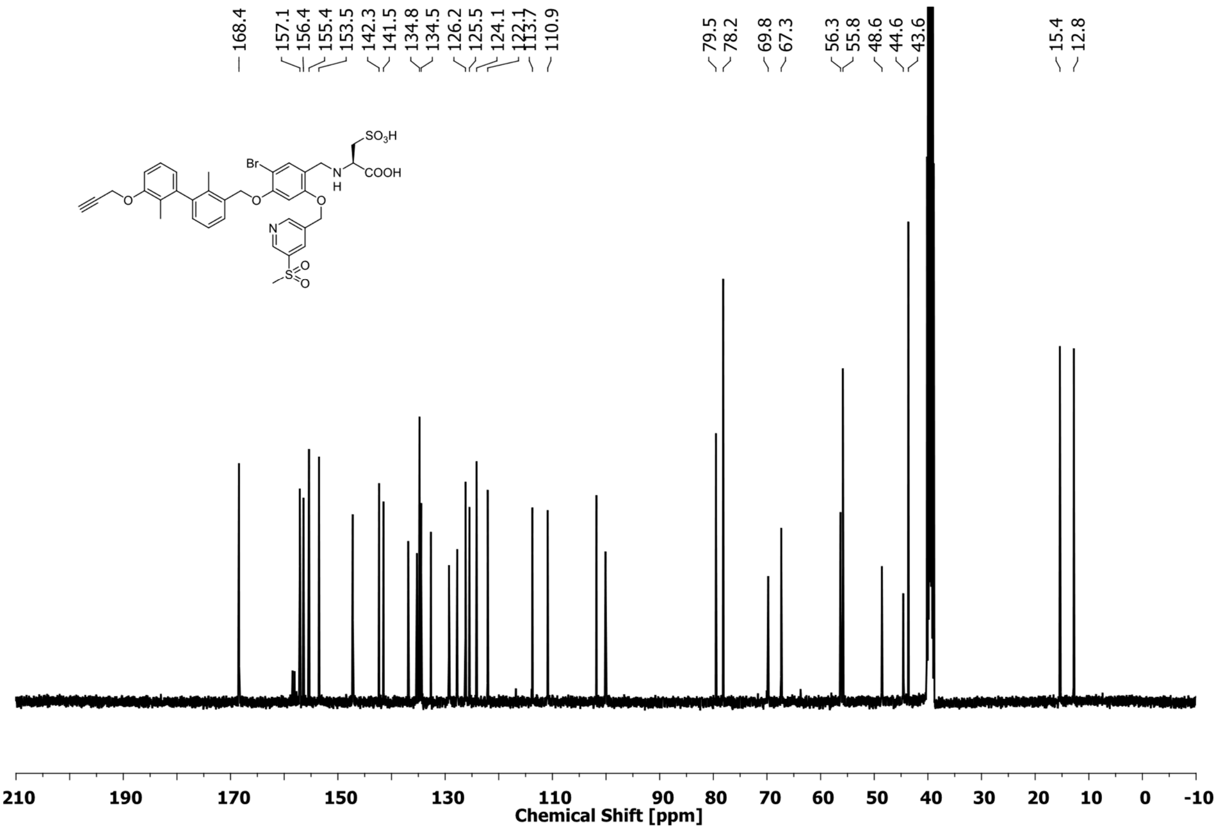

Figure S14: ^{1}H NMR spectrum (DMSO-d_6, 101 MHz, 298 K) of compound **35**.

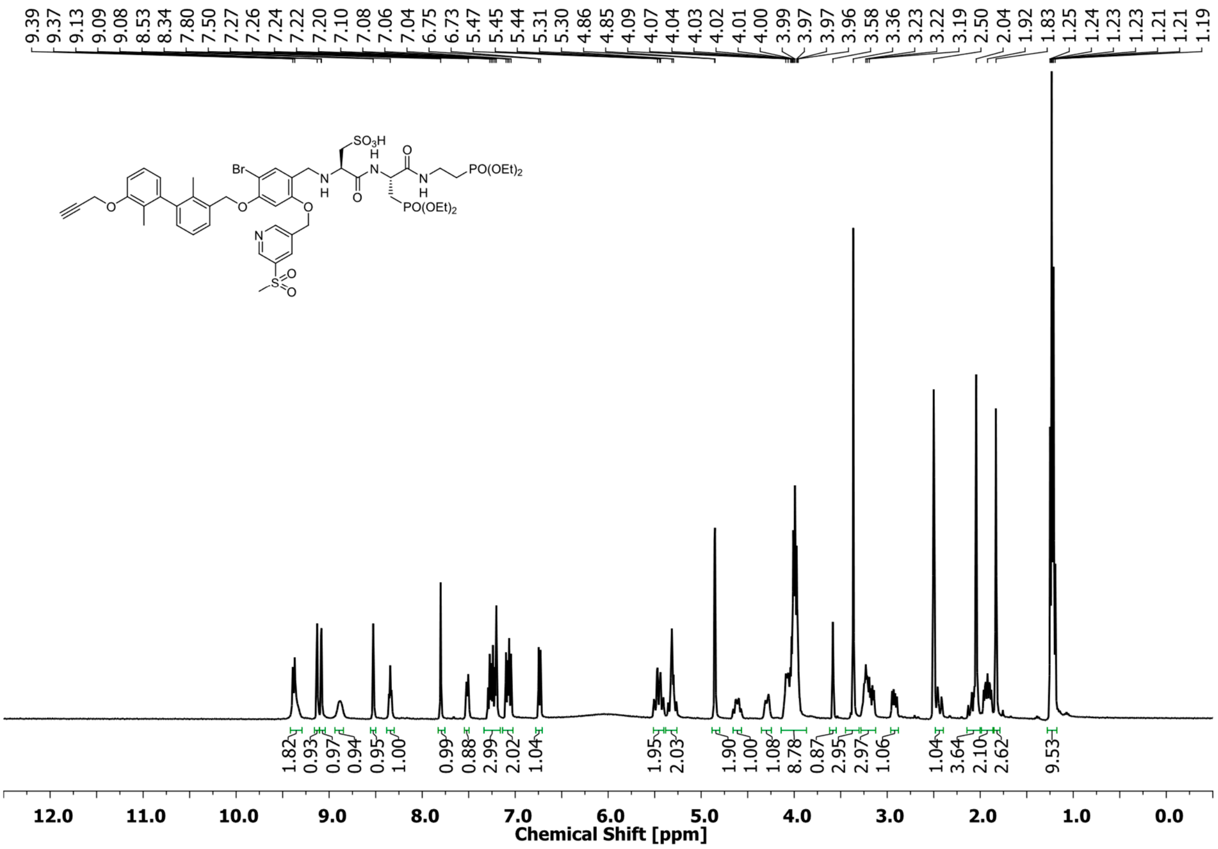

Figure S15: ^{1}H NMR spectrum (DMSO-d_6, 400 MHz, 298 K) of compound **6**.

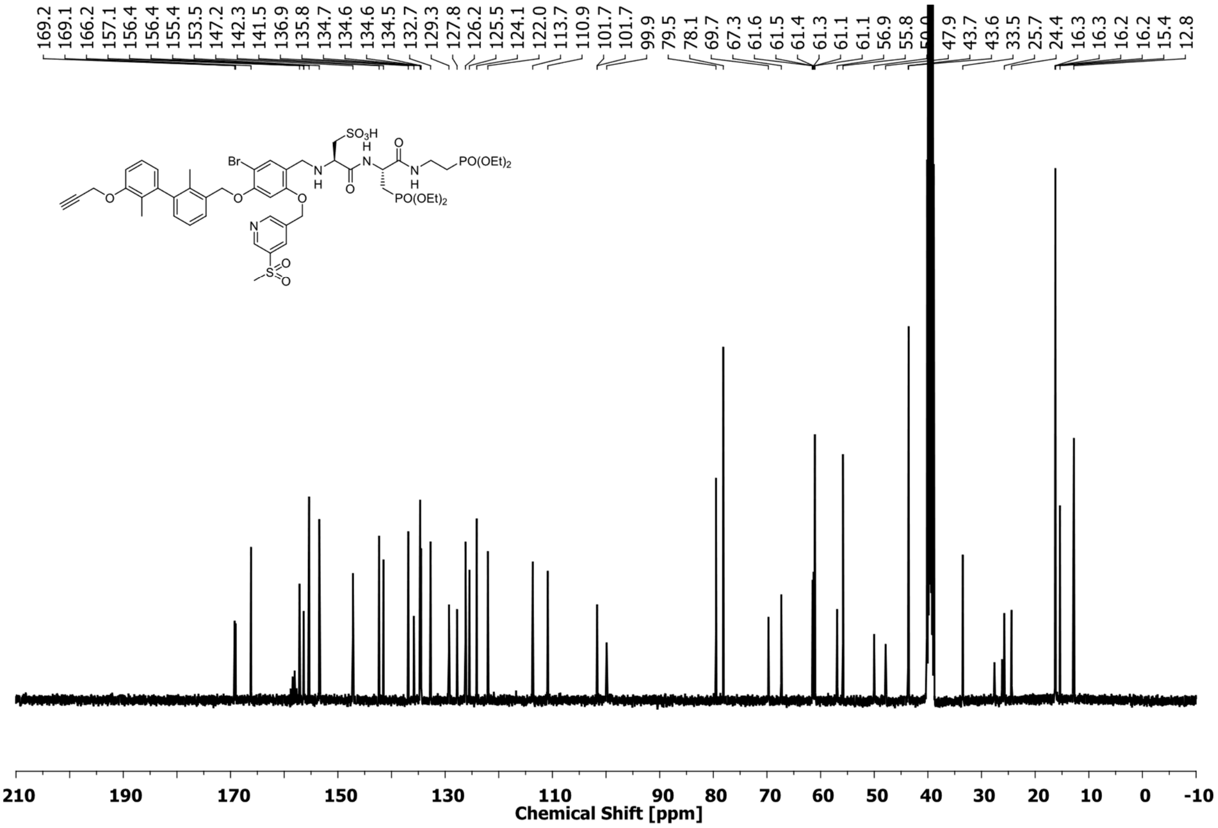

Figure S16: ^{13}C NMR spectrum (DMSO-d_6, 101 MHz, 298 K) of compound **6**.

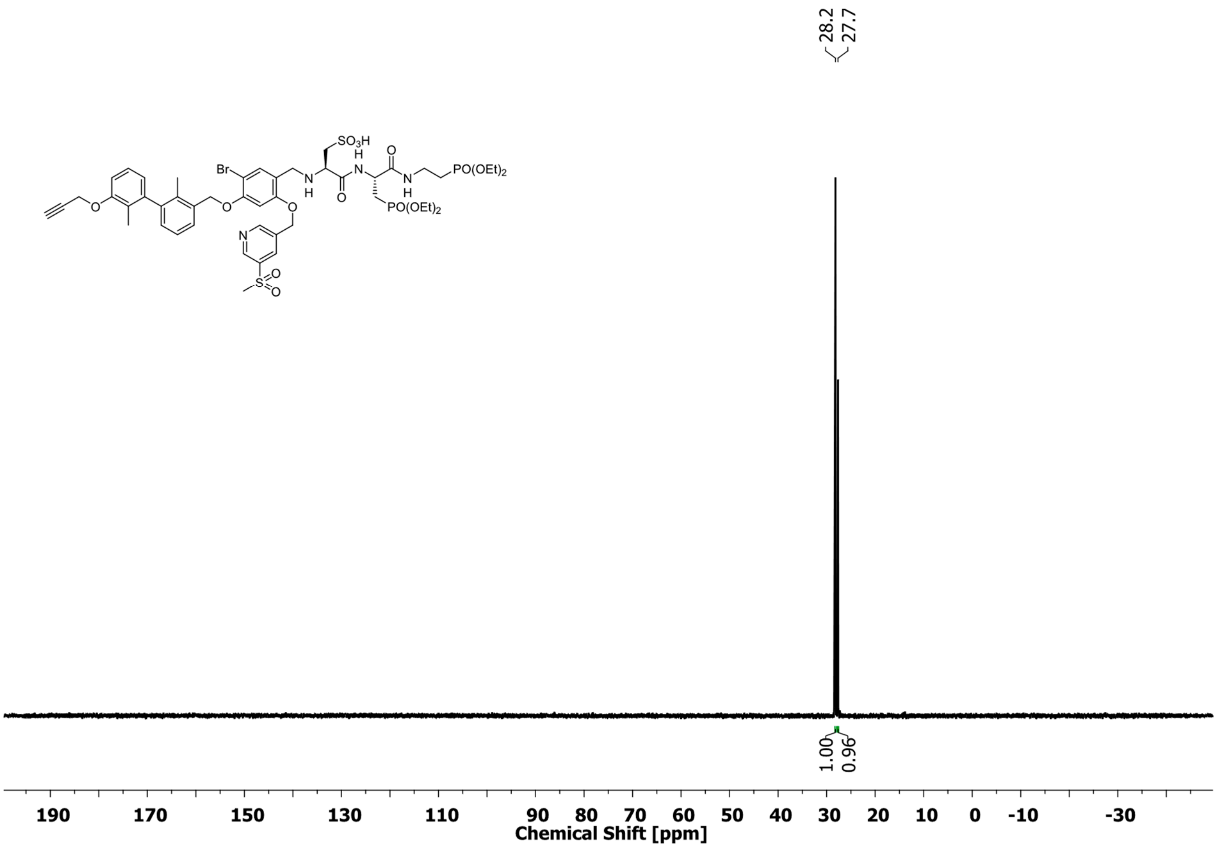

Figure S17: ^{31}P NMR spectrum (DMSO-d_6, 162 MHz, 298 K) of compound **6**.

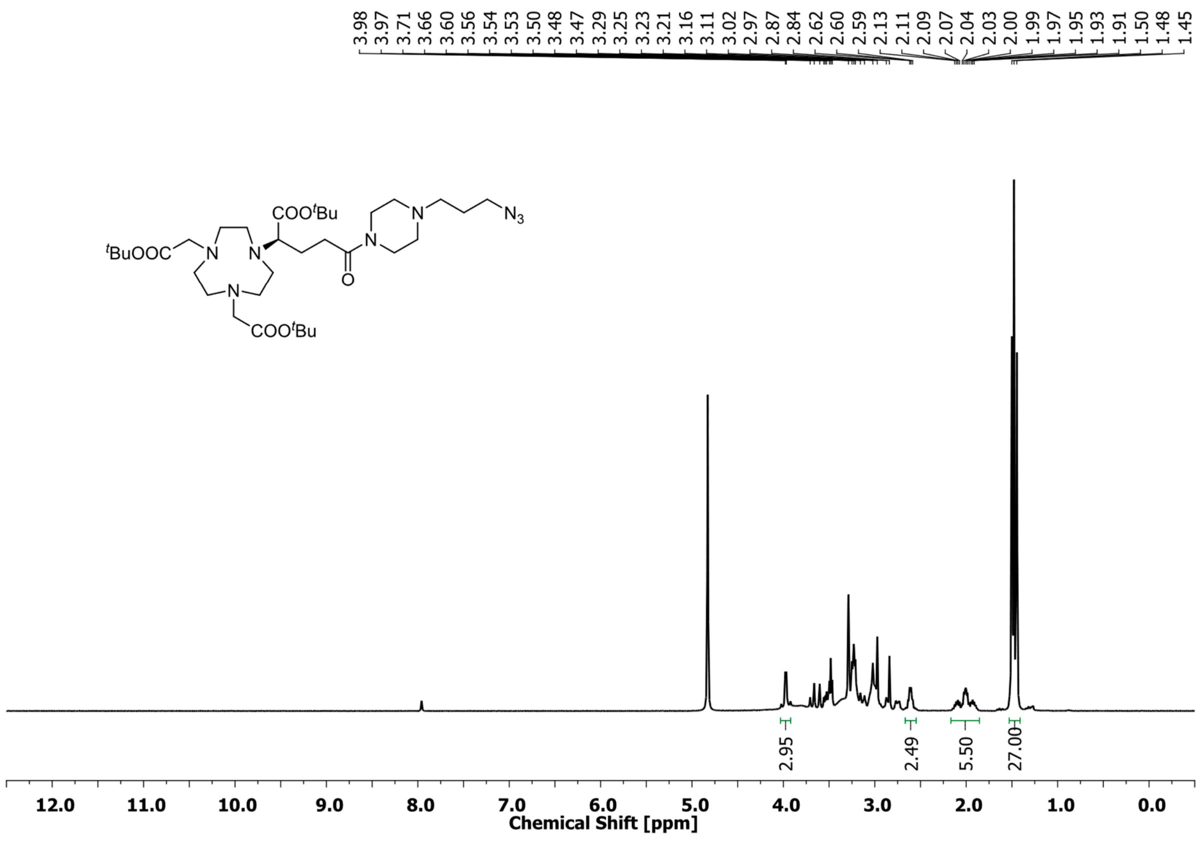

Figure S18: ^{1}H NMR spectrum (DMSO-d_6, 600 MHz, 298 K) of compound **5**.

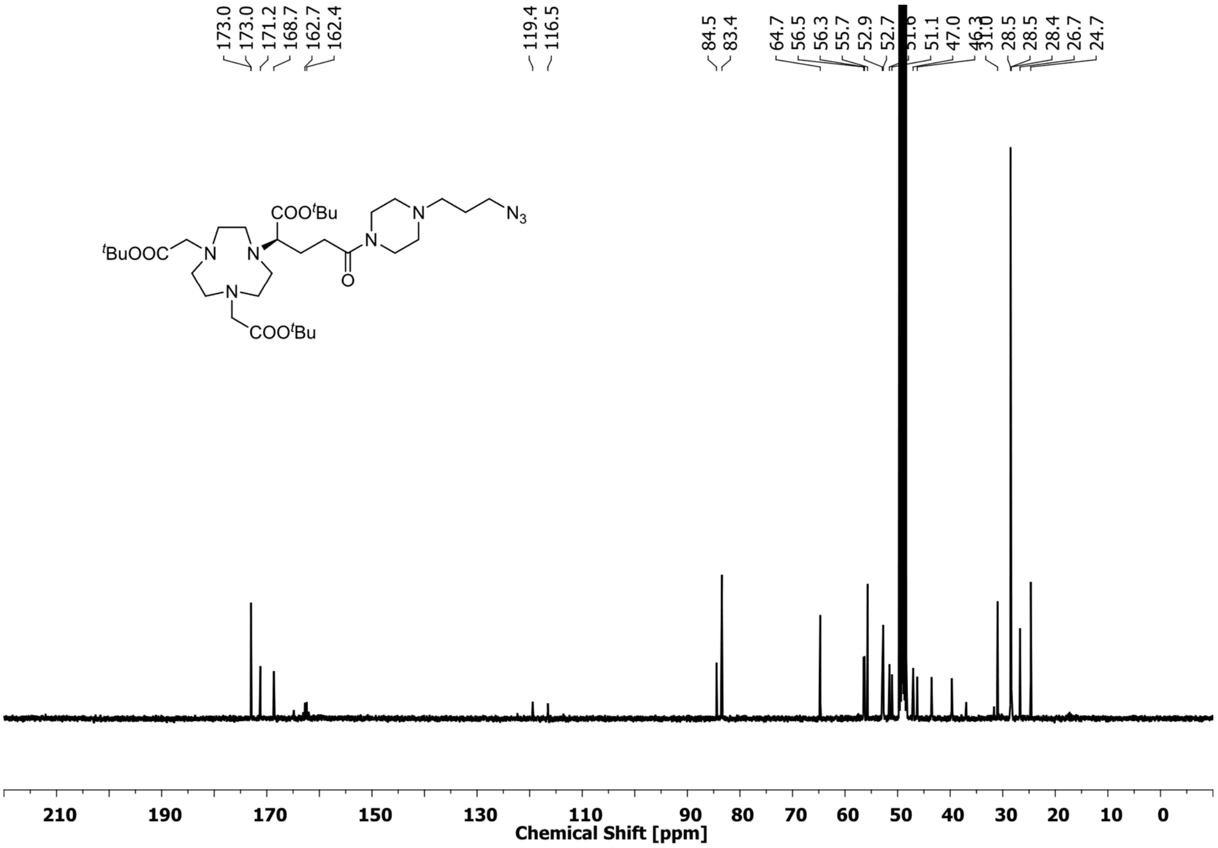

Figure S19: ^{13}C NMR spectrum (DMSO-d_6, 151 MHz, 298 K) of compound **5.**

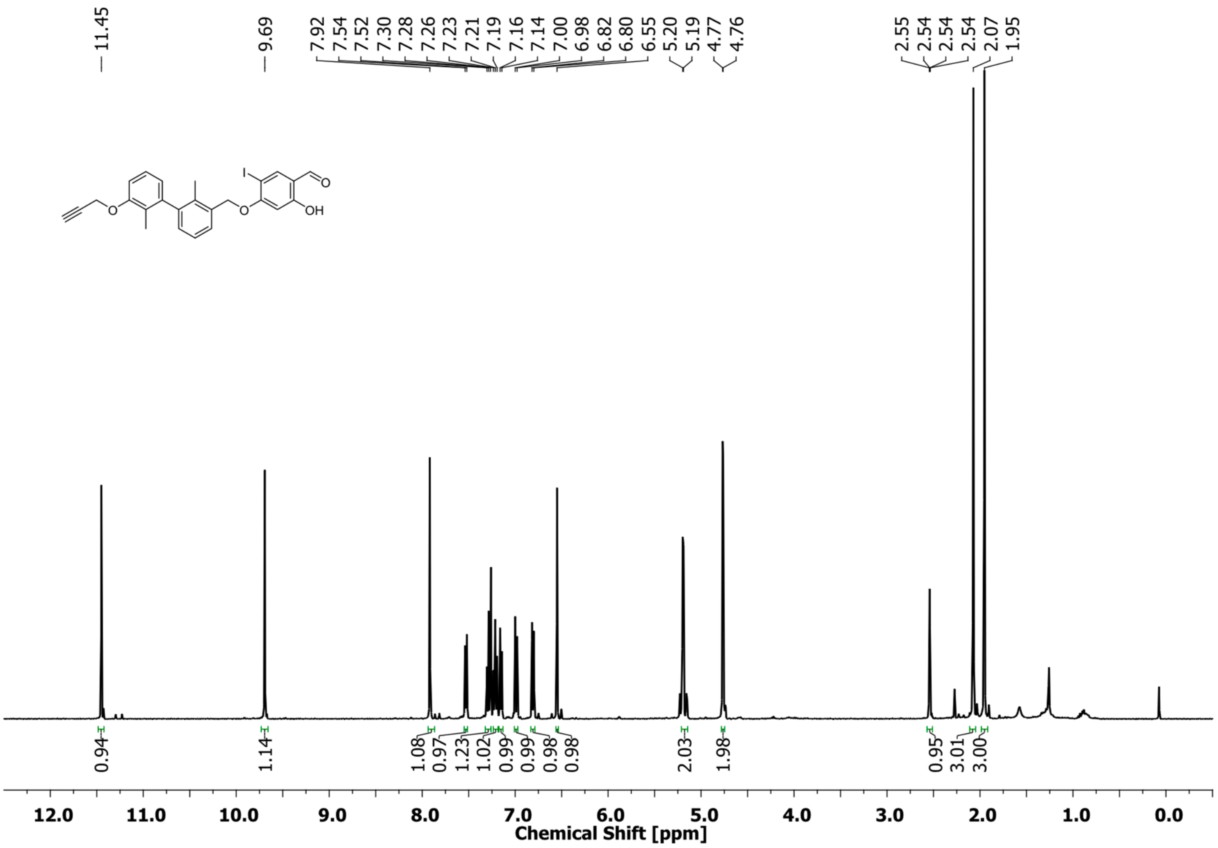

Figure S20: ^{1}H NMR spectrum ($CDCl_3$, 400 MHz, 298 K) of compound **33**.

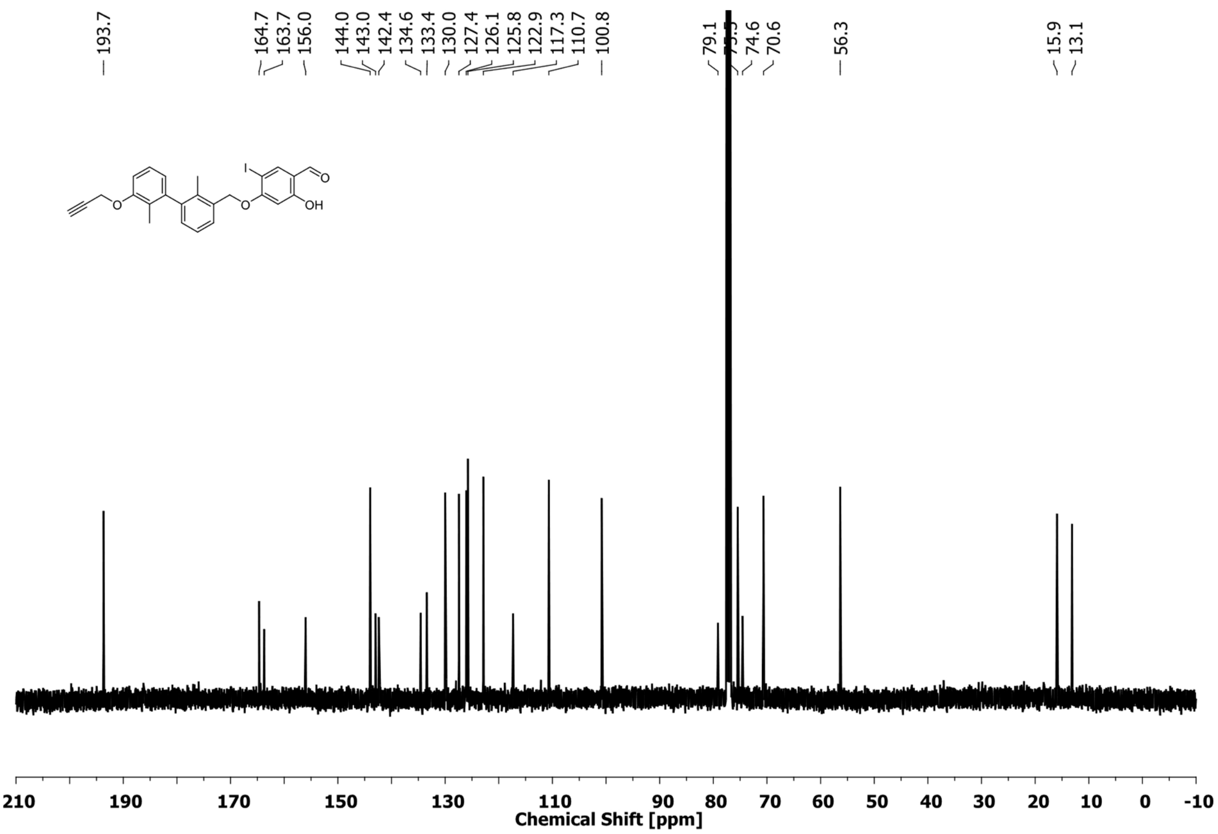

Figure S21: ^{13}C NMR spectrum ($CDCl_3$, 101 MHz, 298 K) of compound **33**.

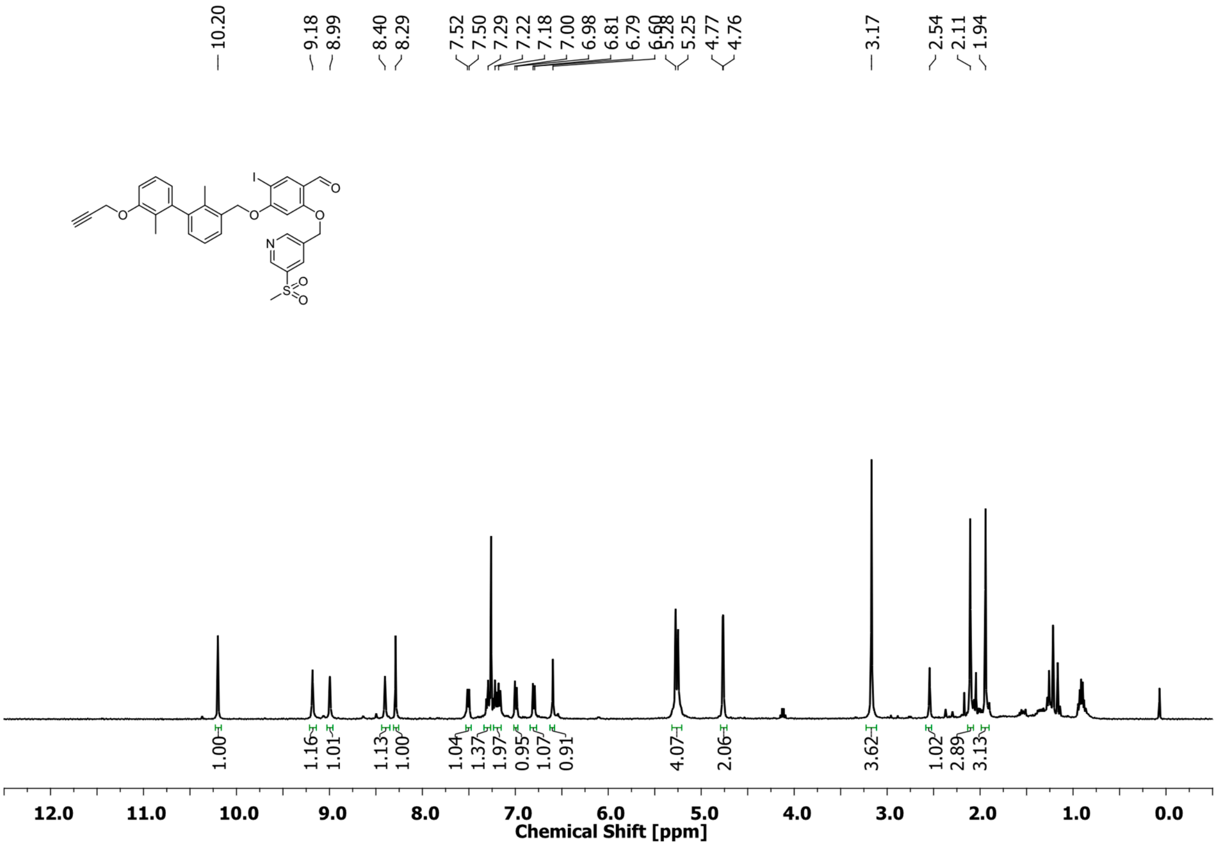

Figure S22: ^{1}H NMR spectrum ($CDCl_3$, 400 MHz, 298 K) of compound **12**.

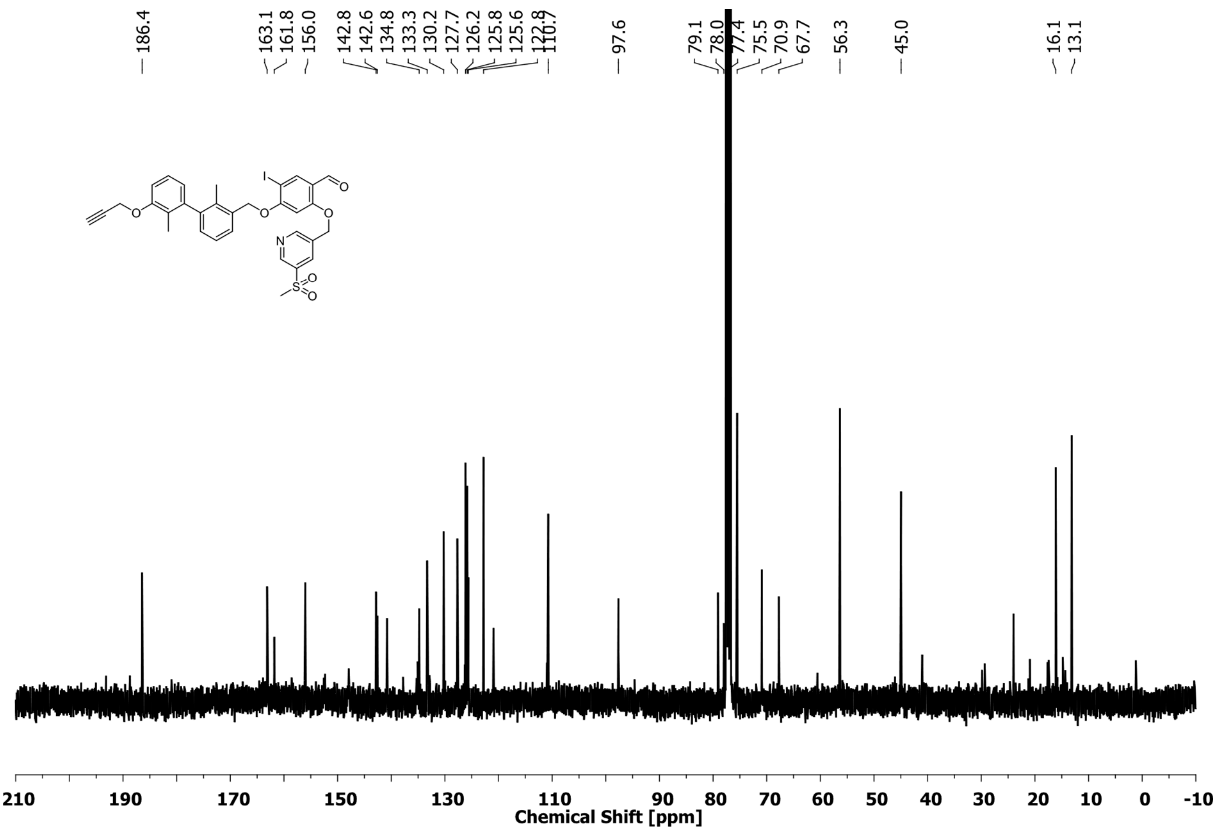

Figure S23: ^{13}C NMR spectrum ($CDCl_3$, 101 MHz, 298 K) of compound **12**.

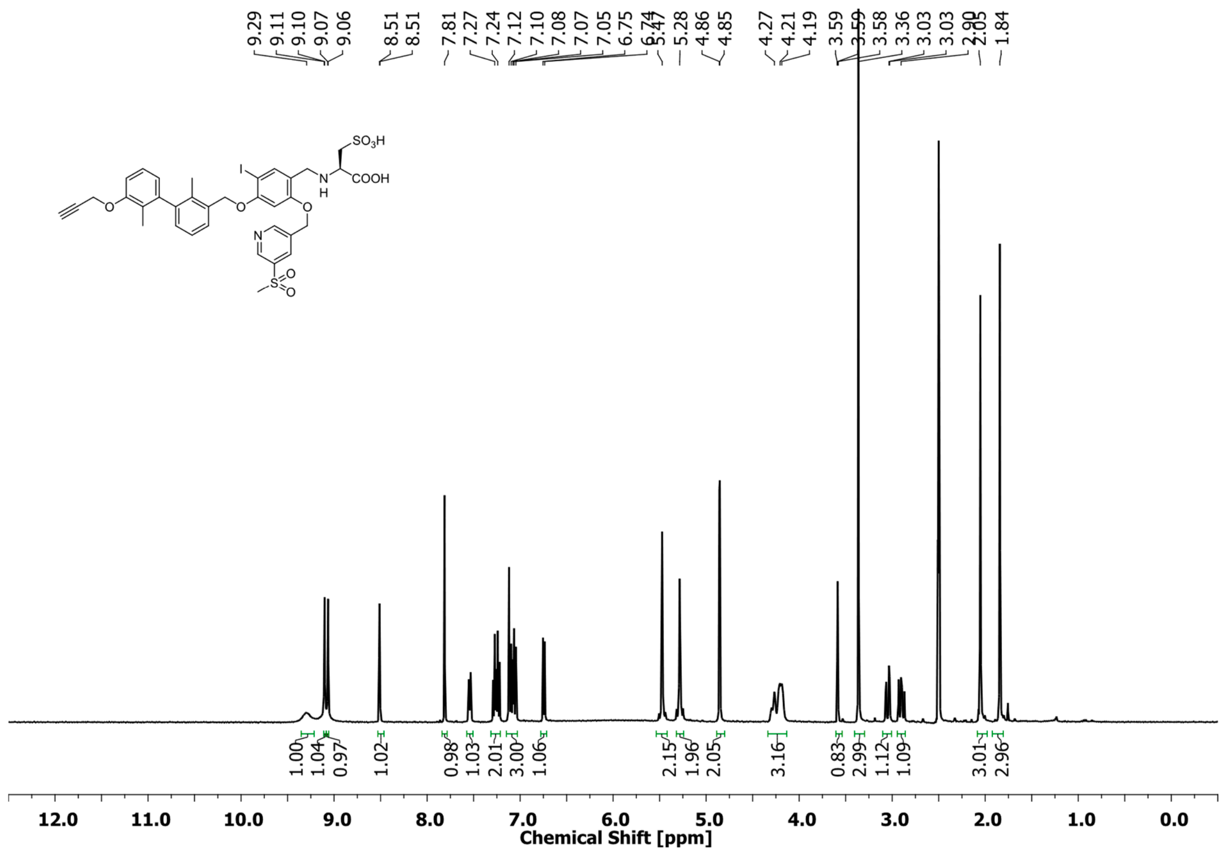

Figure S24: ^{1}H NMR spectrum (DMSO-d_6, 400 MHz, 298 K) of compound **36**.

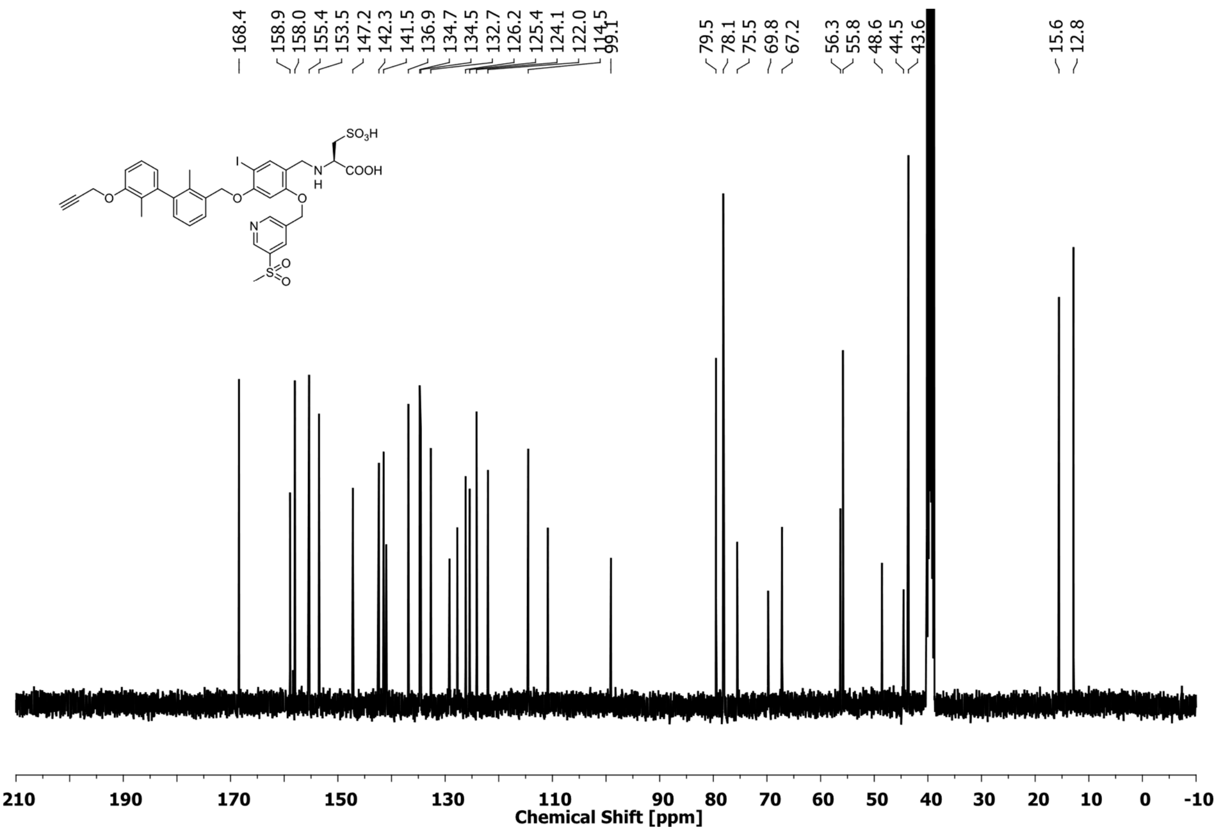

Figure S25: ^{13}C NMR spectrum (DMSO-d_6, 101 MHz, 298 K) of compound **36**.

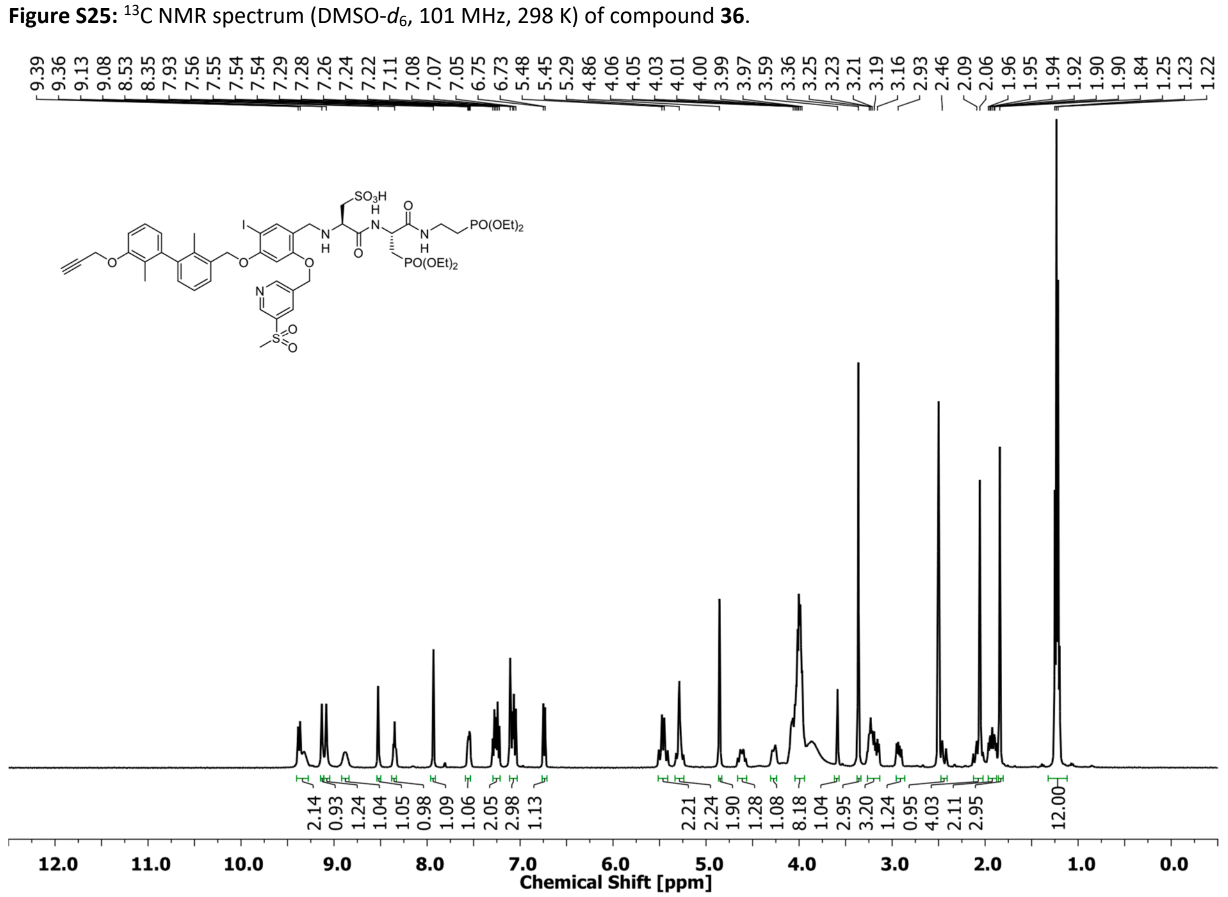

Figure S26: ^{1}H NMR spectrum (DMSO-d_6, 400 MHz, 298 K) of compound **7**.

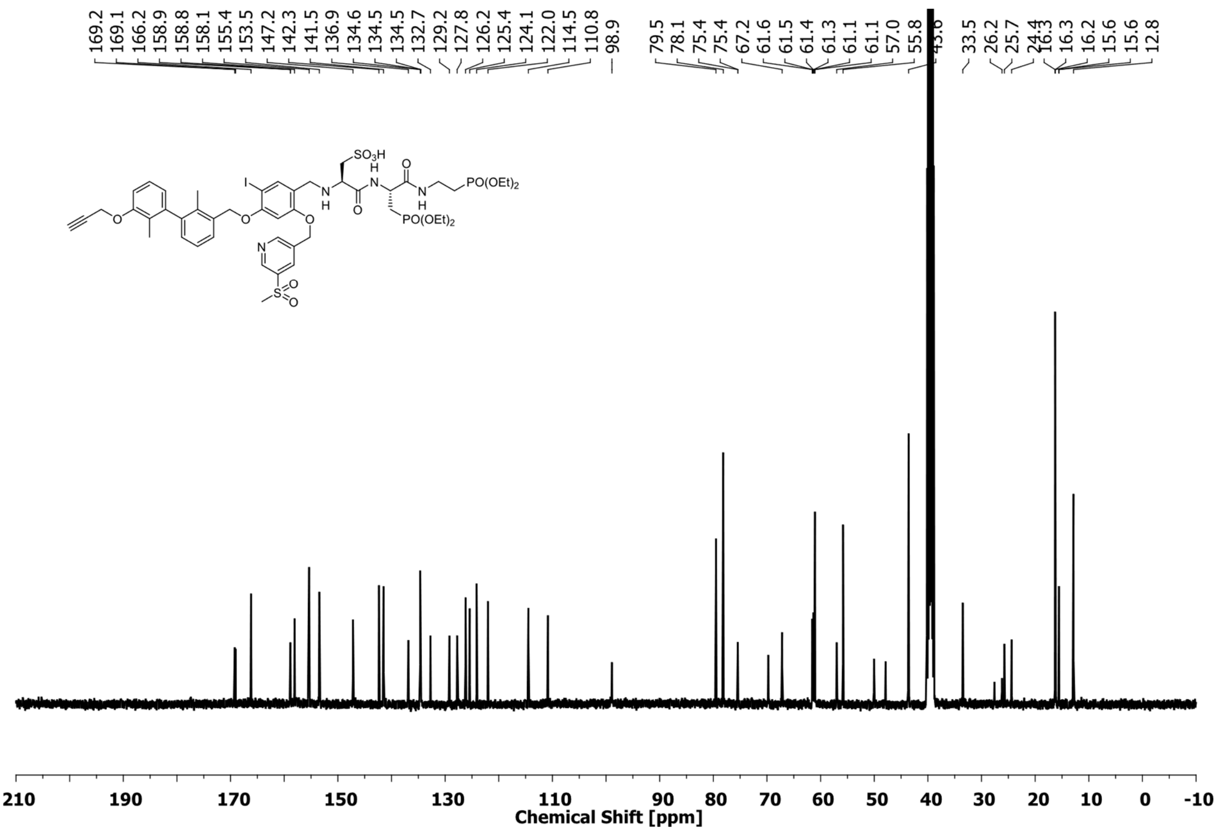

Figure S27: ^{13}C NMR spectrum (DMSO-d_6, 101 MHz, 298 K) of compound **7**.

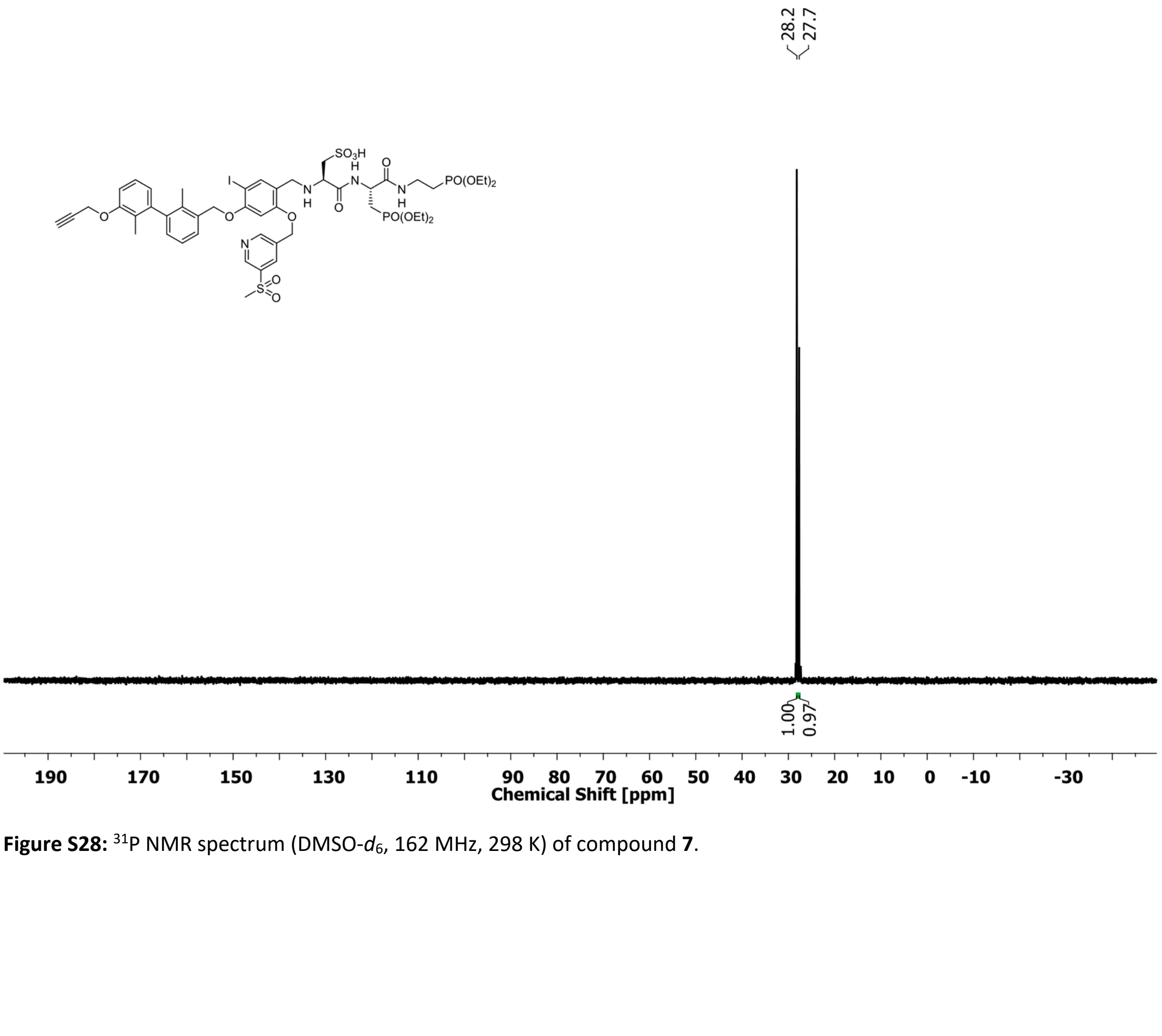

Figure S28: ^{31}P NMR spectrum (DMSO-d_6, 162 MHz, 298 K) of compound **7**.

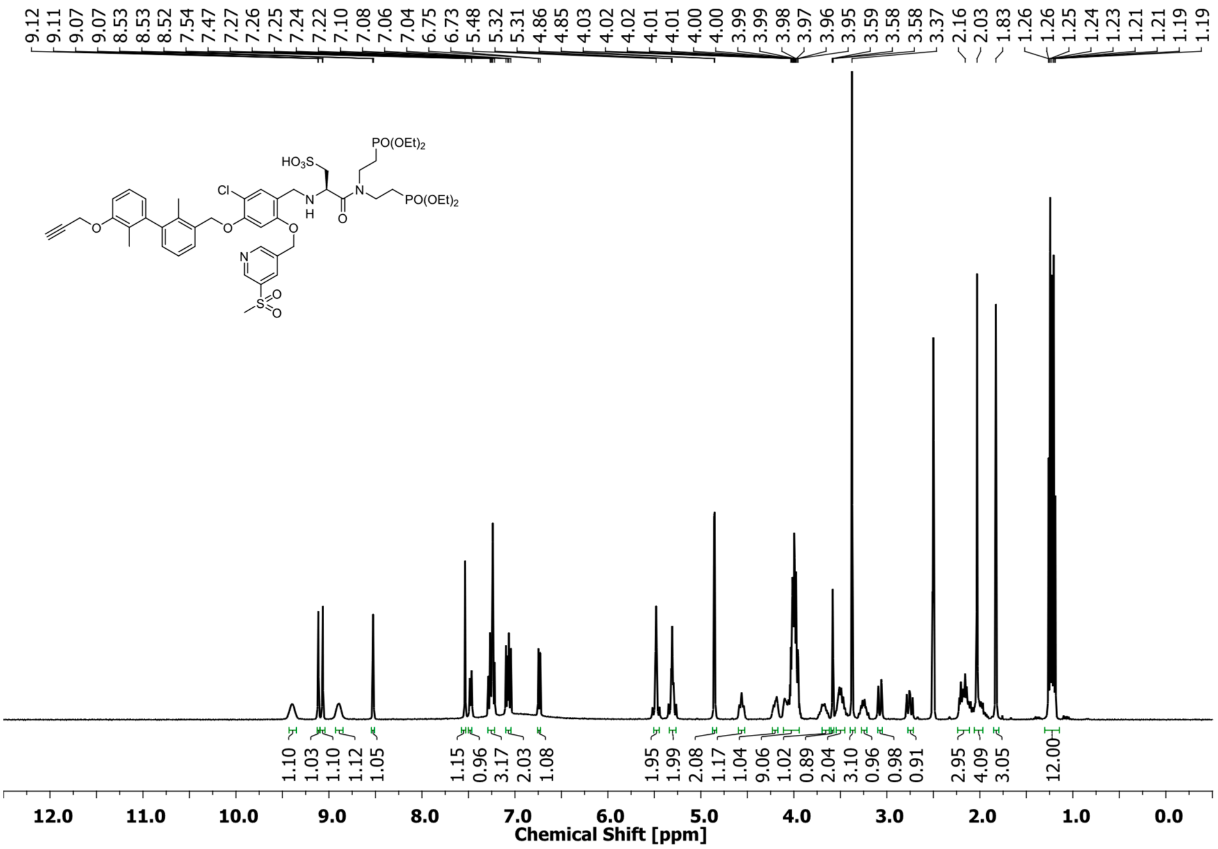

Figure S29: ^{1}H NMR spectrum (DMSO-d_6, 400 MHz, 298 K) of compound **8**.

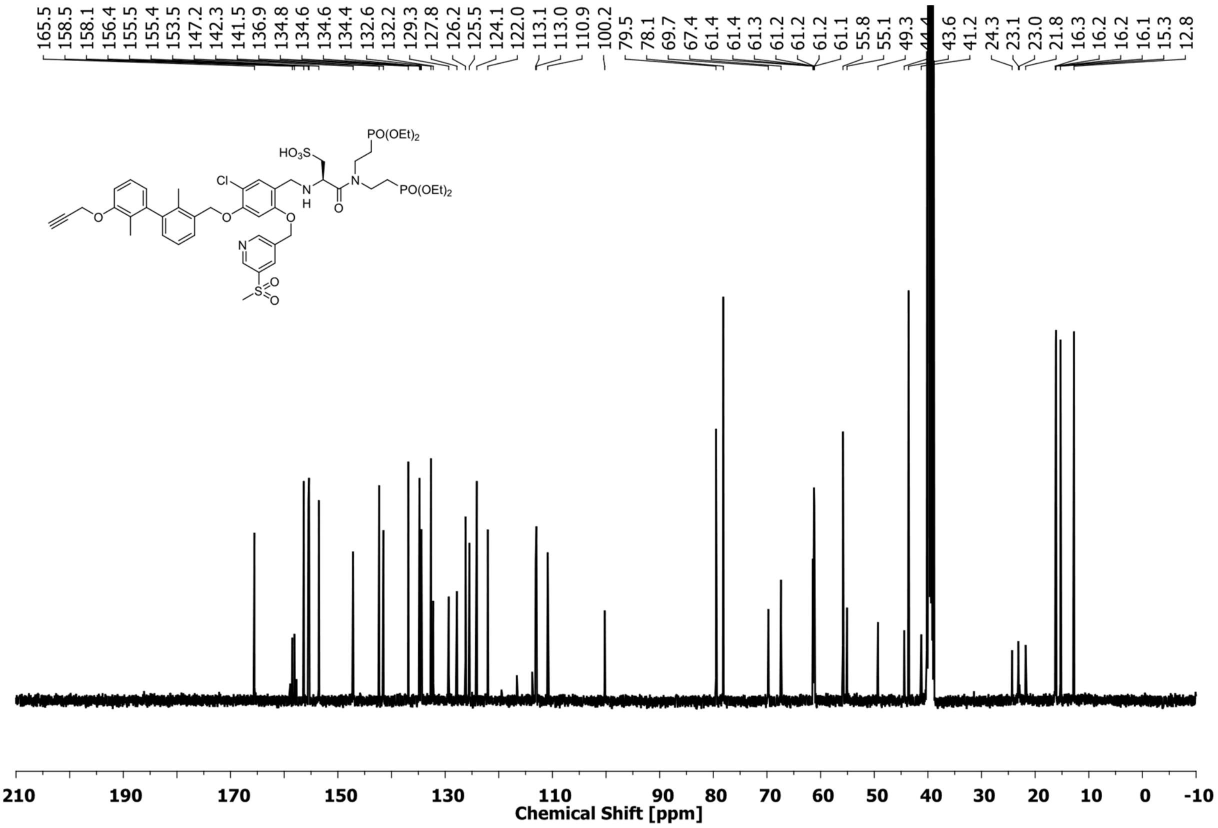

Figure S30: ^{13}C NMR spectrum (DMSO-d_6, 101 MHz, 298 K) of compound **8**.

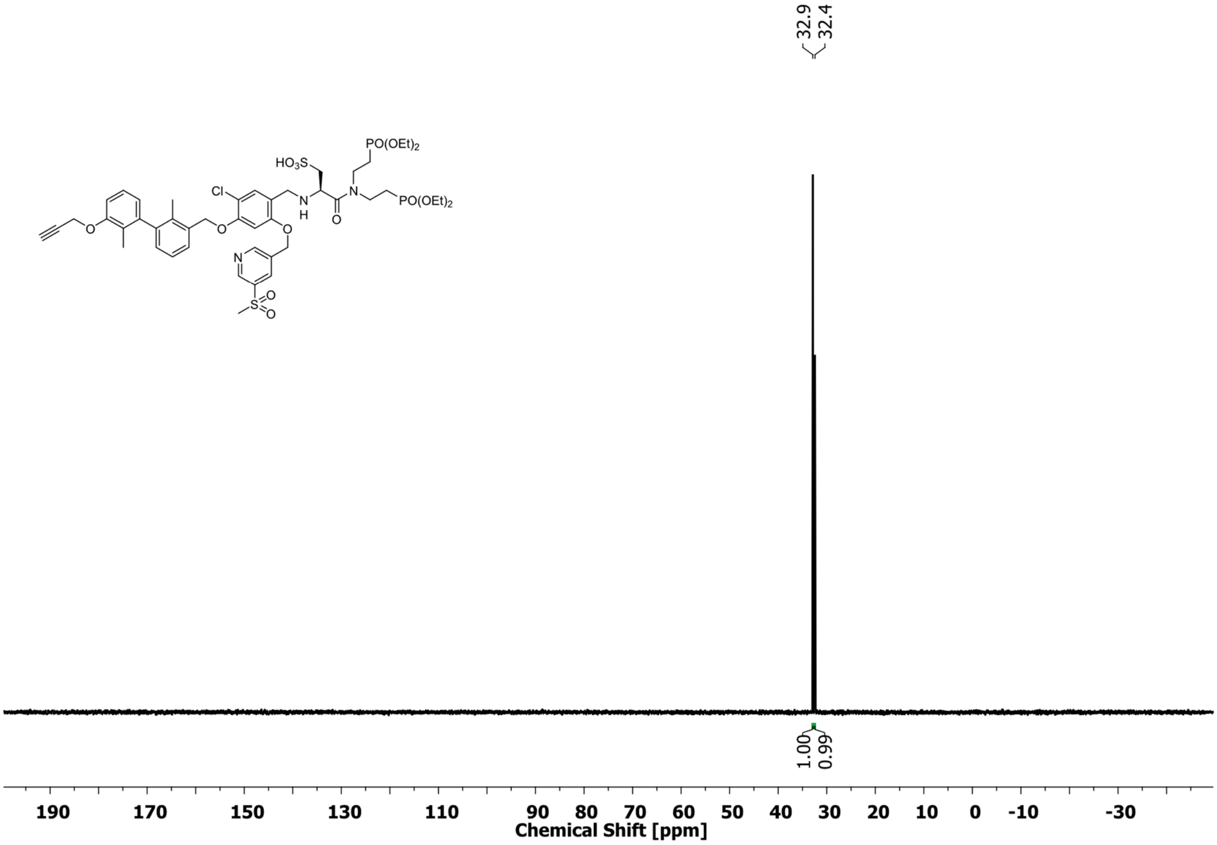

Figure S31: ^{31}P NMR spectrum (DMSO-d_6, 162 MHz, 298 K) of compound **8**.

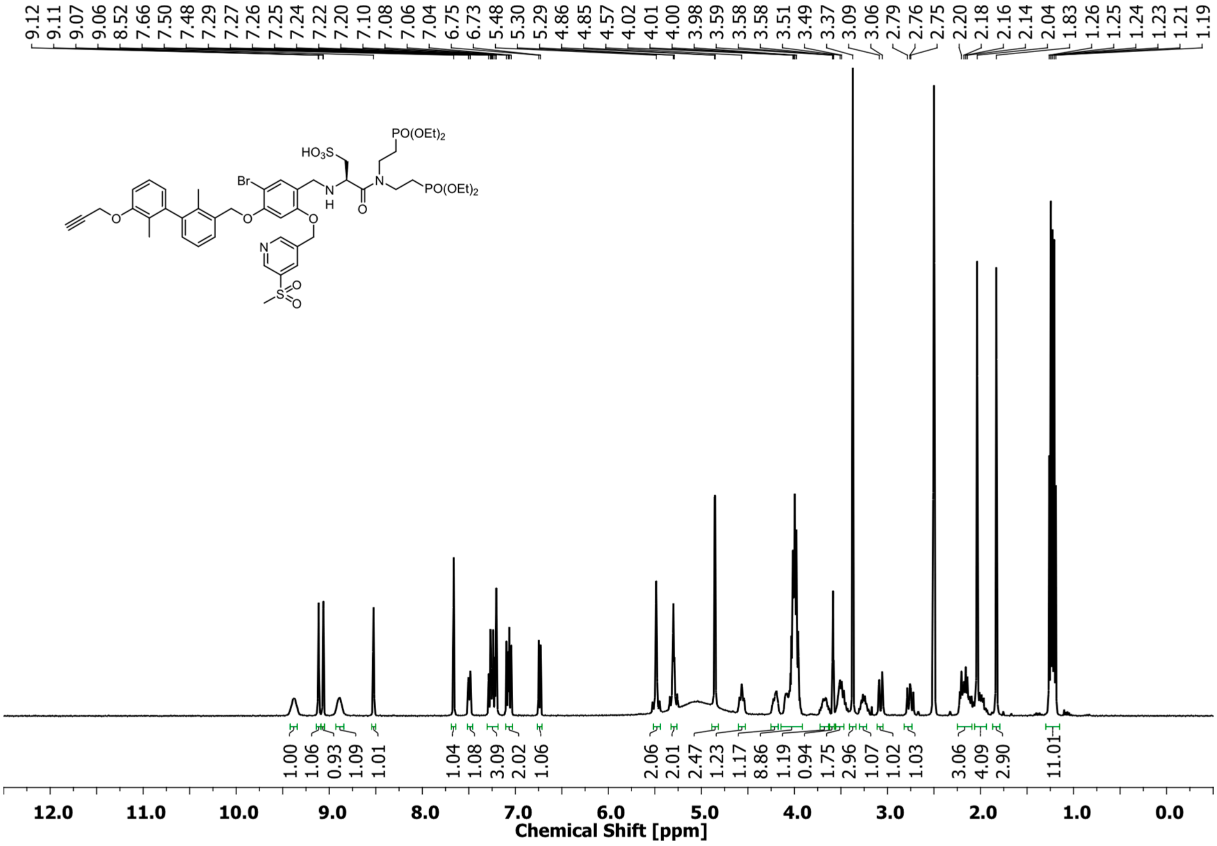

Figure S32: ^{1}H NMR spectrum (DMSO-d_6, 400 MHz, 298 K) of compound **9**.

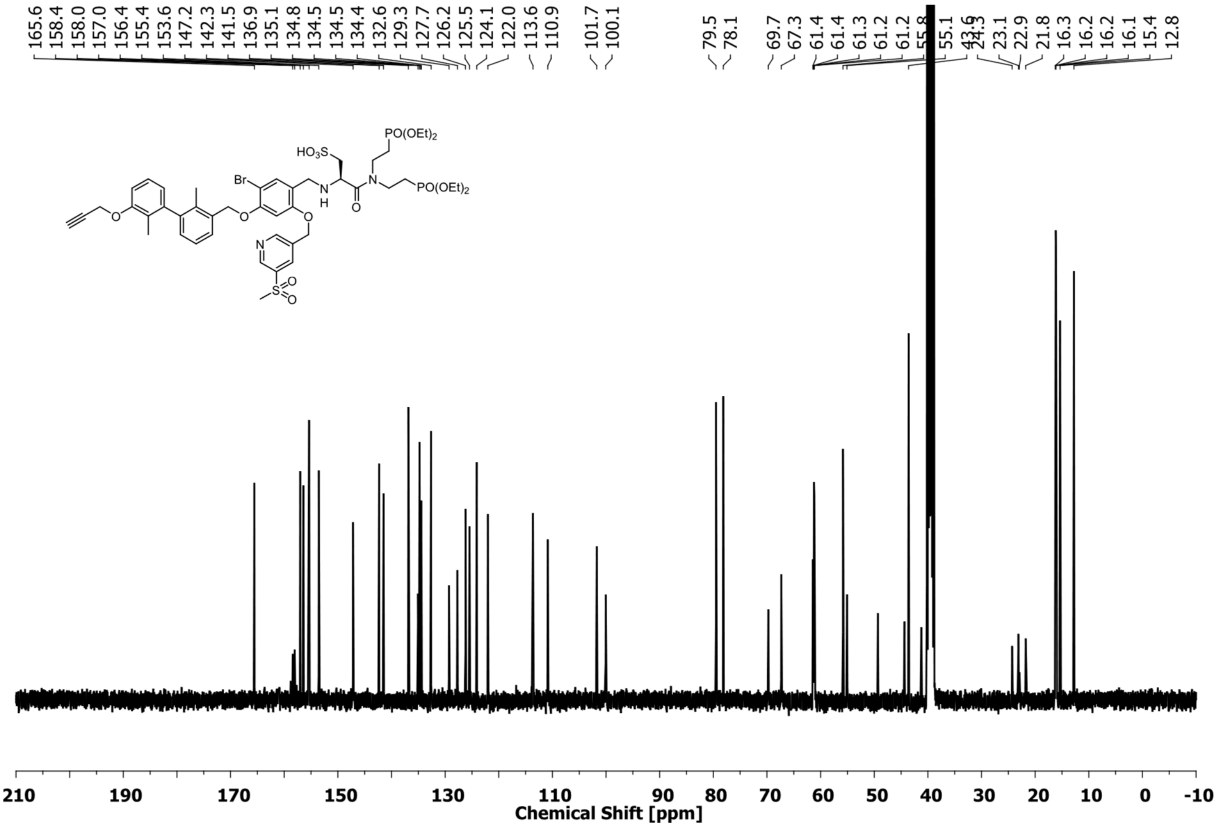

Figure S33: ^{13}C NMR spectrum (DMSO-d_6, 101 MHz, 298 K) of compound **9**.

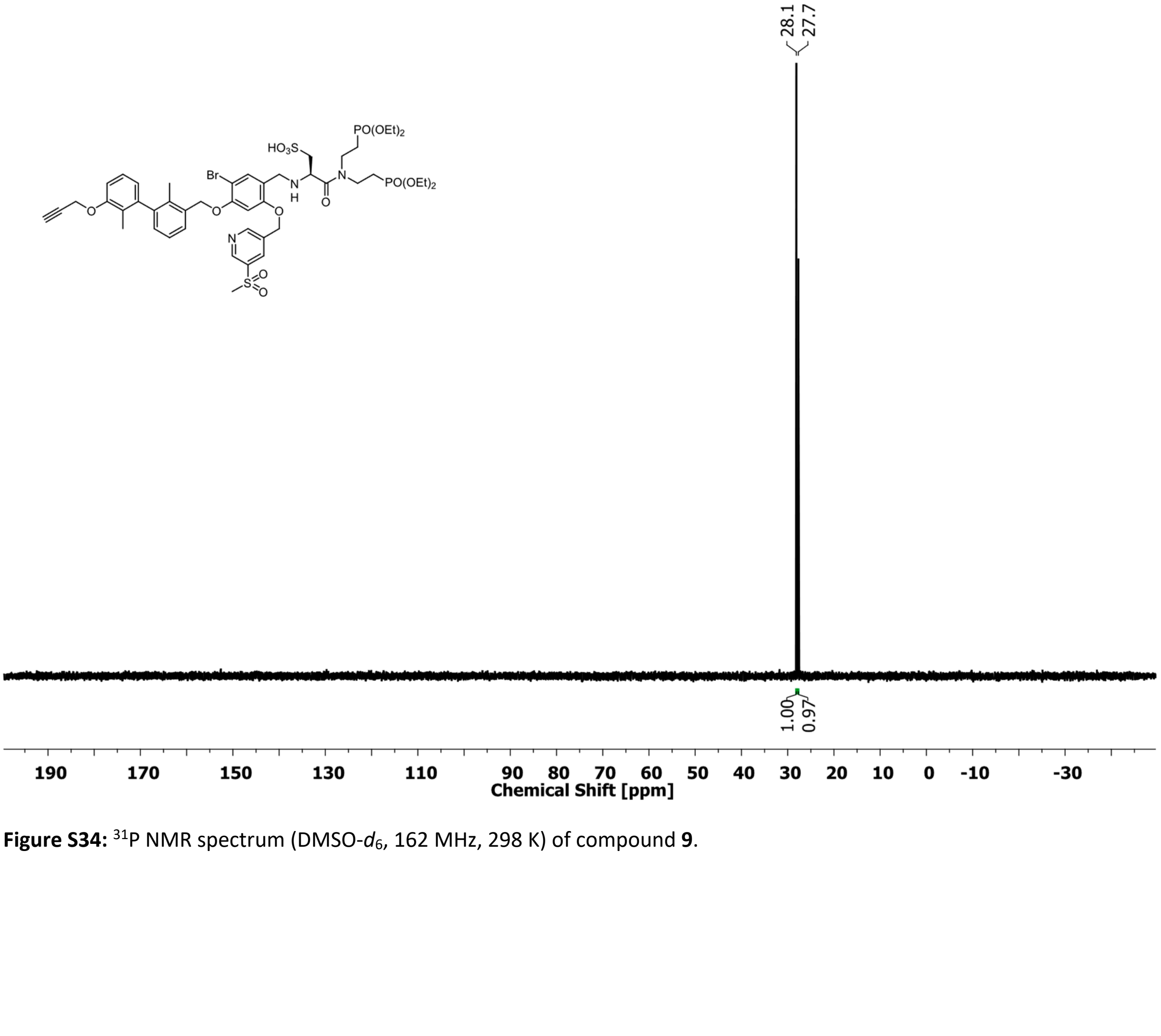

Figure S34: ^{31}P NMR spectrum (DMSO-d_6, 162 MHz, 298 K) of compound **9**.

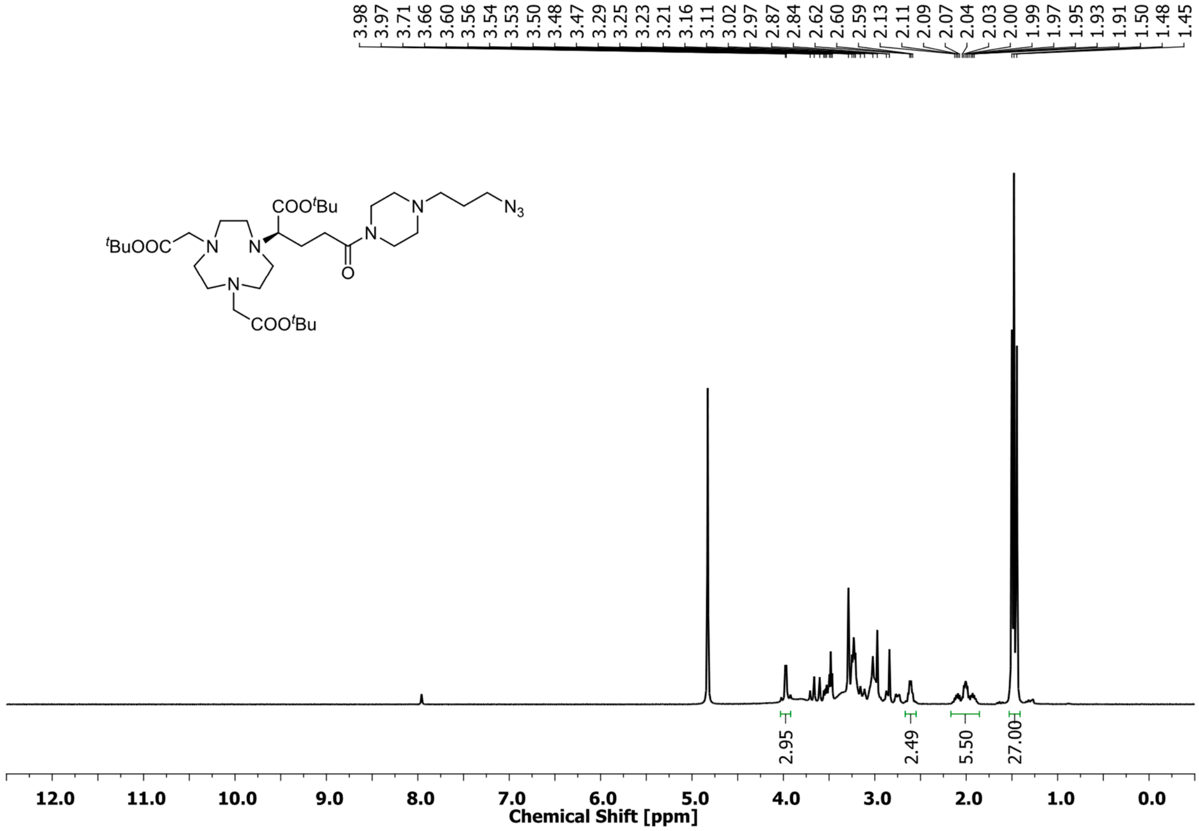

Figure S35: ^{1}H NMR spectrum (DMSO-d_6, 600 MHz, 298 K) of compound **5**.

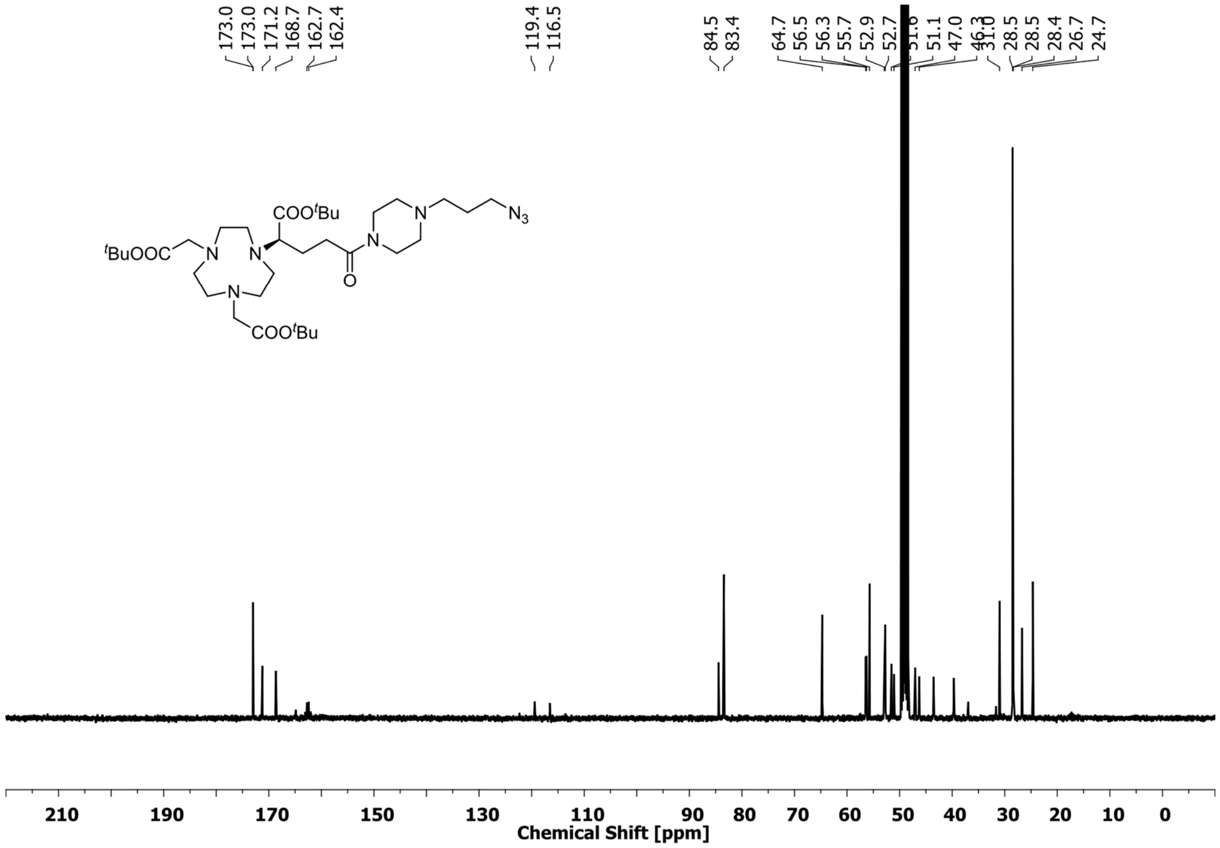

Figure S36: ^{13}C NMR spectrum (DMSO-d_6, 151 MHz, 298 K) of compound **5**.

3. IR spectra of literature unknown compounds

Attenuated Total Reflectance Fourier Transform Infrared (ATR-FTIR) spectra were recorded with a Thermo Scientific™ Nicolet™ iS™ 5 FT-IR-device.

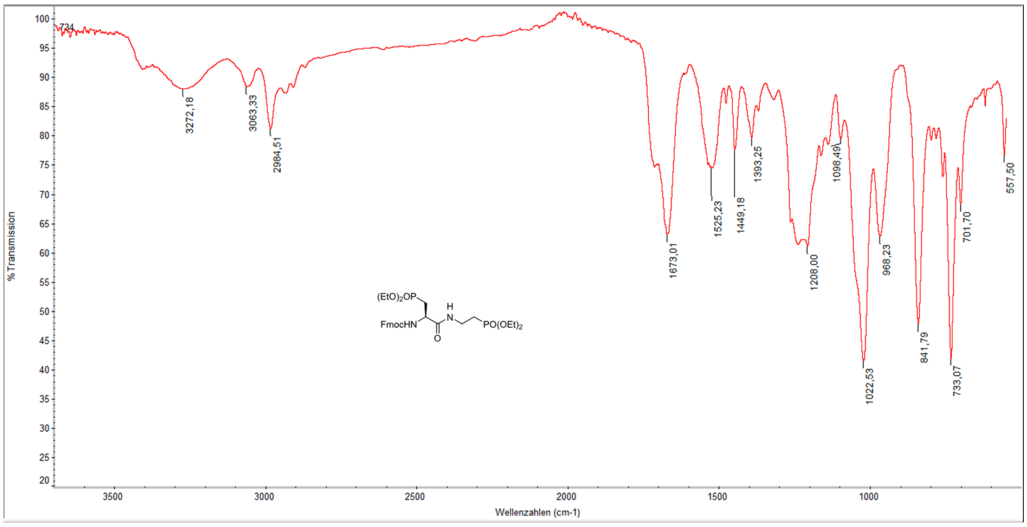

Figure S37: ATR-IR spectrum of compound **27**.

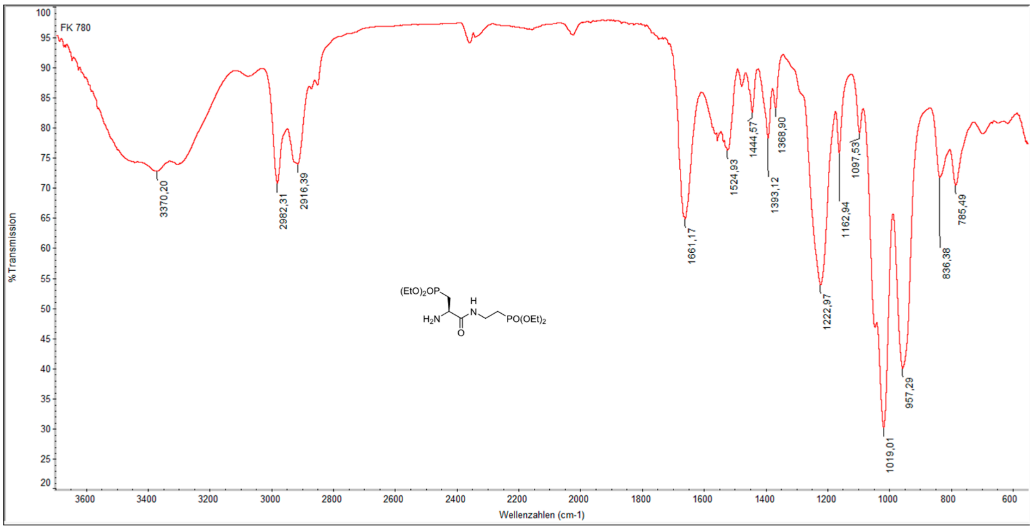

Figure S38: ATR-IR spectrum of compound **13**.

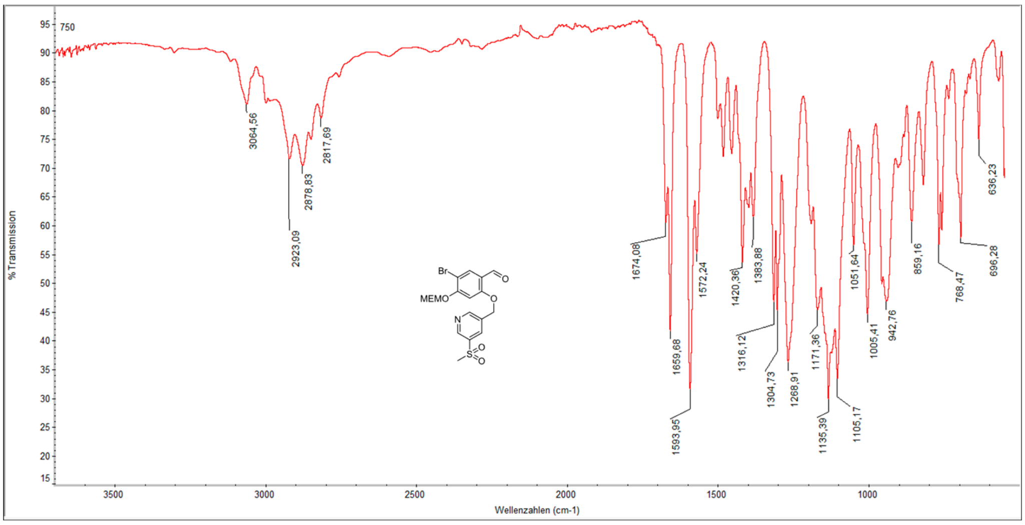

Figure S39: ATR-IR spectrum of compound **24**.

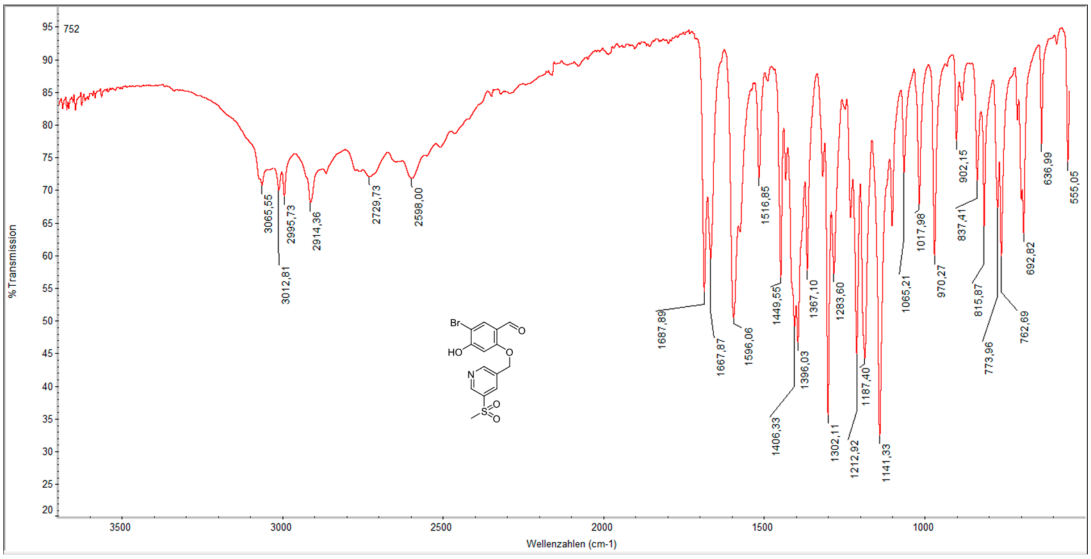

Figure S40: ATR-IR spectrum of compound **17**.

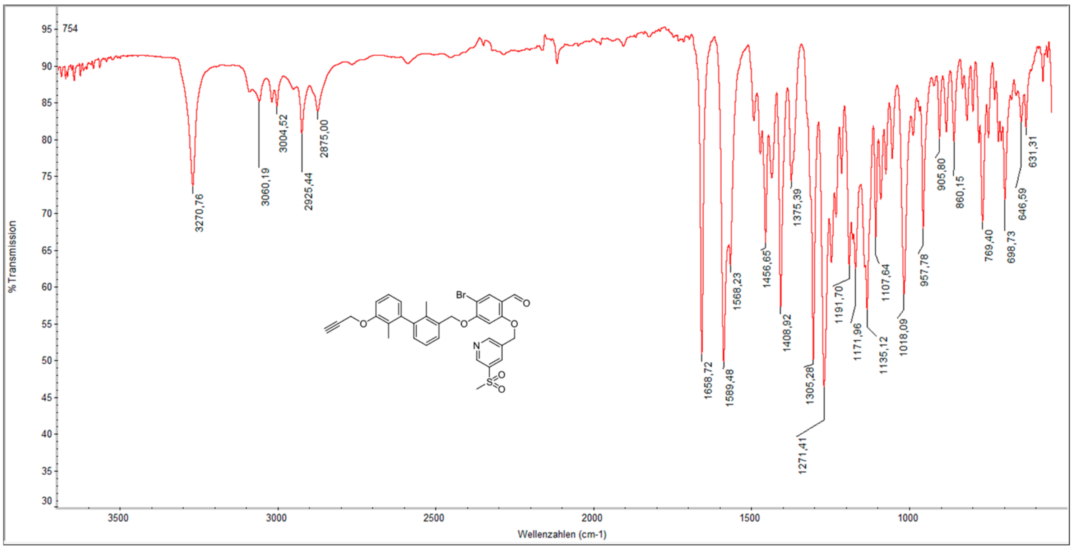

Figure S41: ATR-IR spectrum of compound **11**.

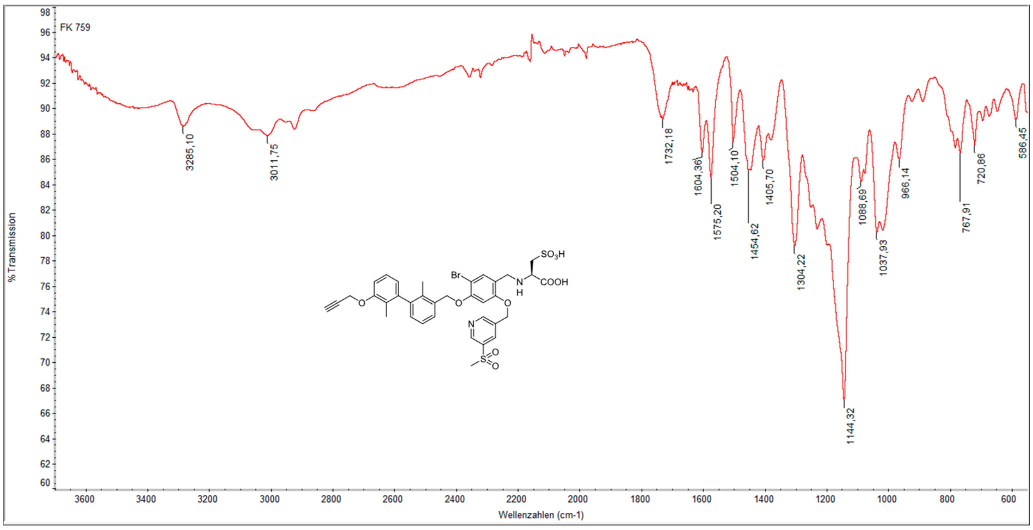

Figure S42: ATR-IR spectrum of compound **35**.

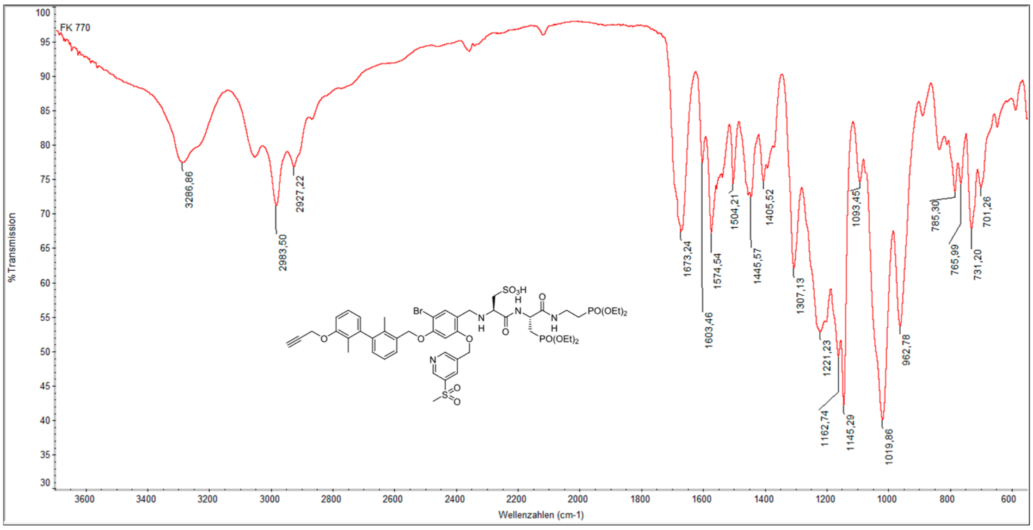

Figure S43: ATR-IR spectrum of compound **6**.

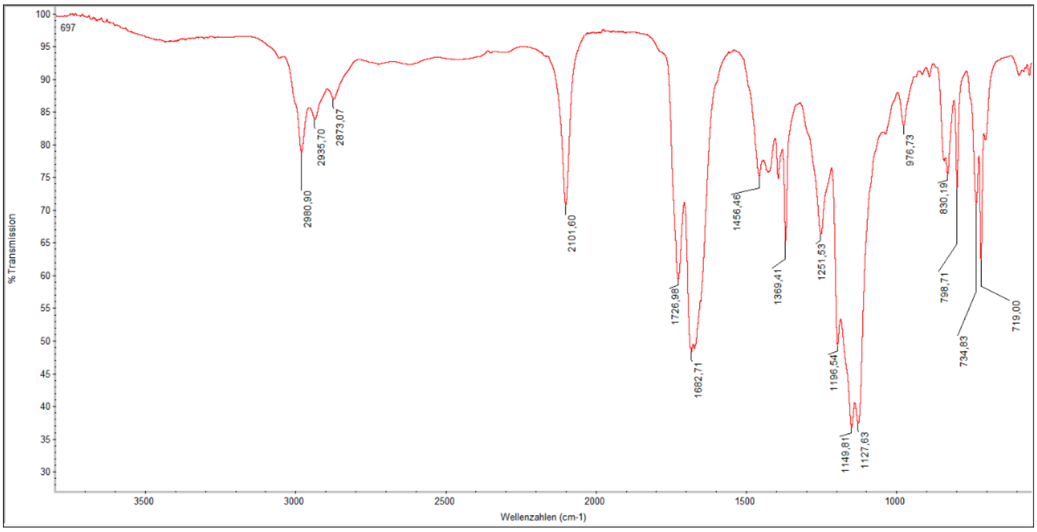

Figure S44: ATR-IR spectrum of compound **5.**

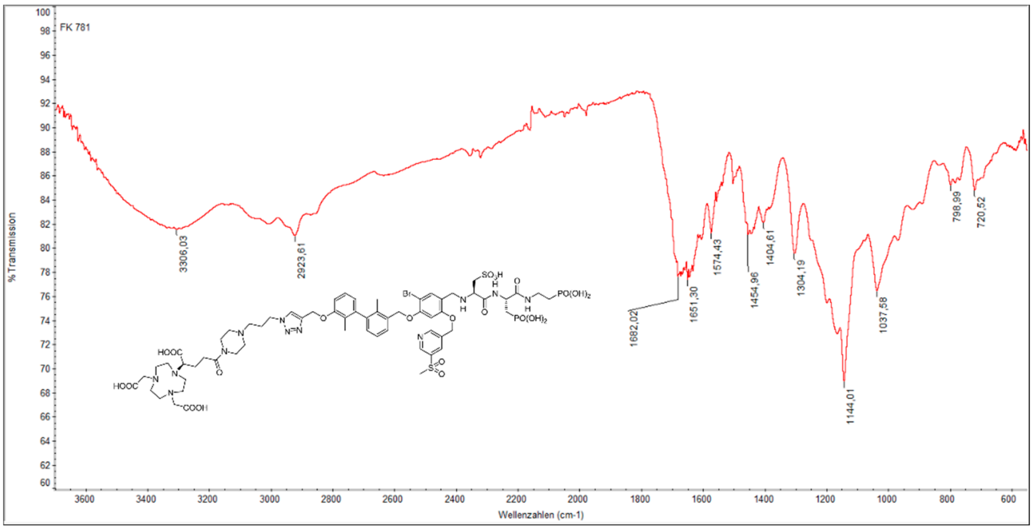

Figure S45: ATR-IR spectrum of compound **1**.

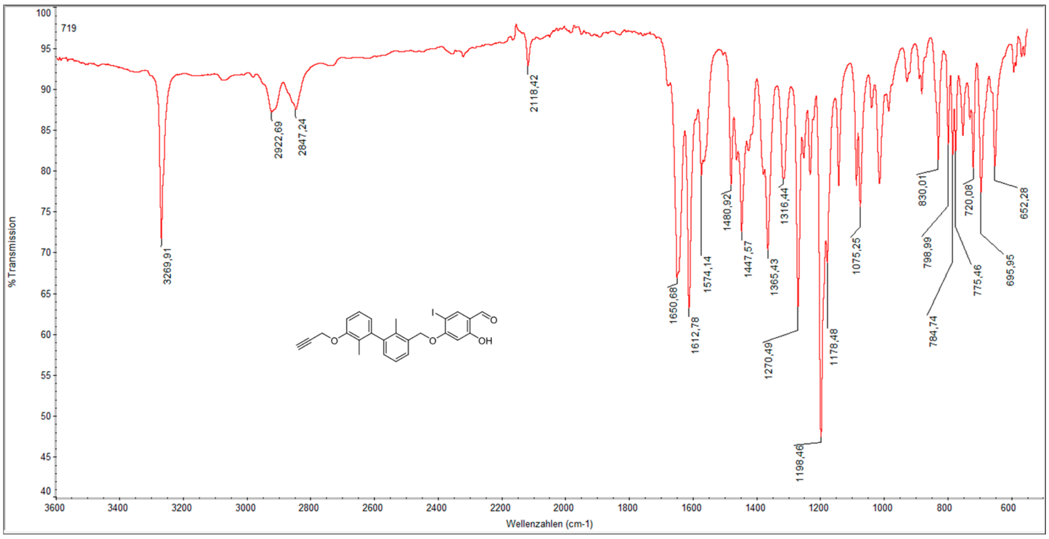

Figure S46: ATR-IR spectrum of compound **33**.

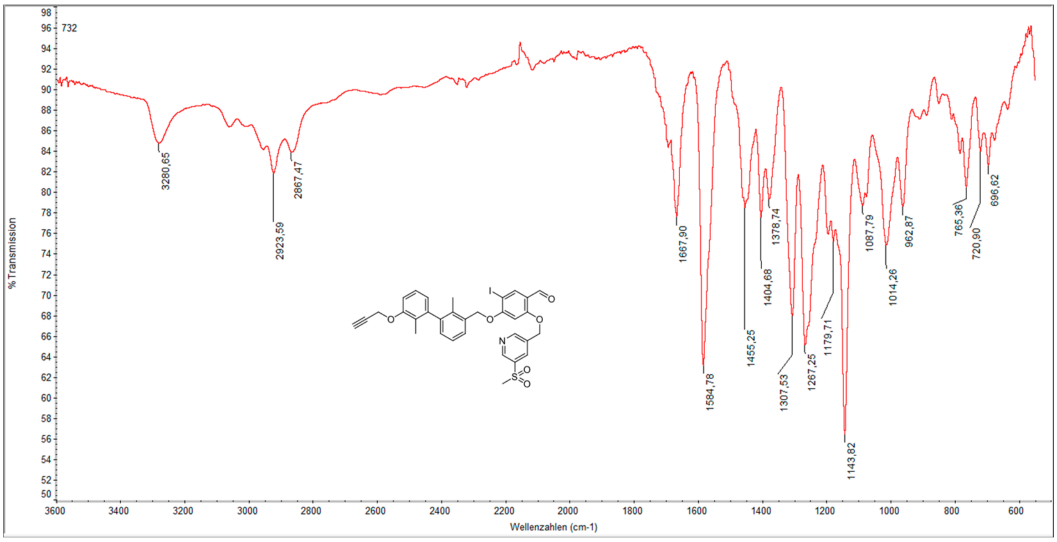

Figure S47: ATR-IR spectrum of compound **12**.

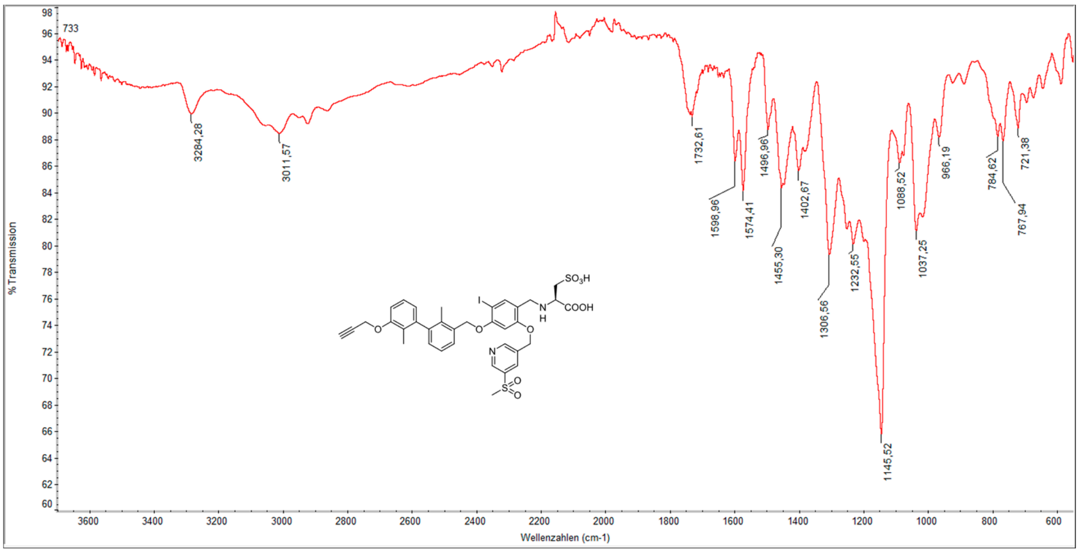

Figure S48: ATR-IR spectrum of compound **36**.

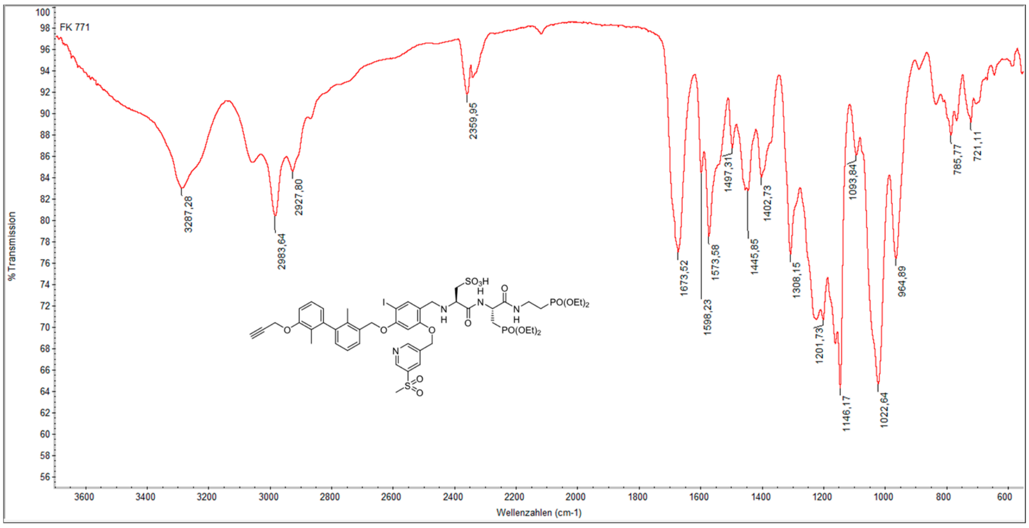

Figure S49: ATR-IR spectrum of compound **7**.

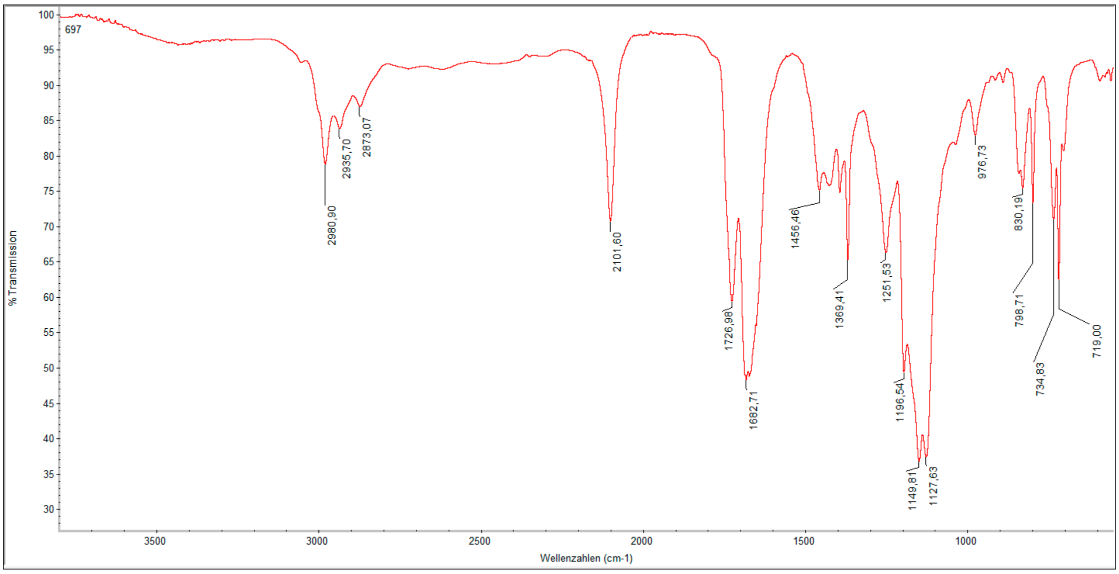

Figure S50: ATR-IR spectrum of compound **23**.

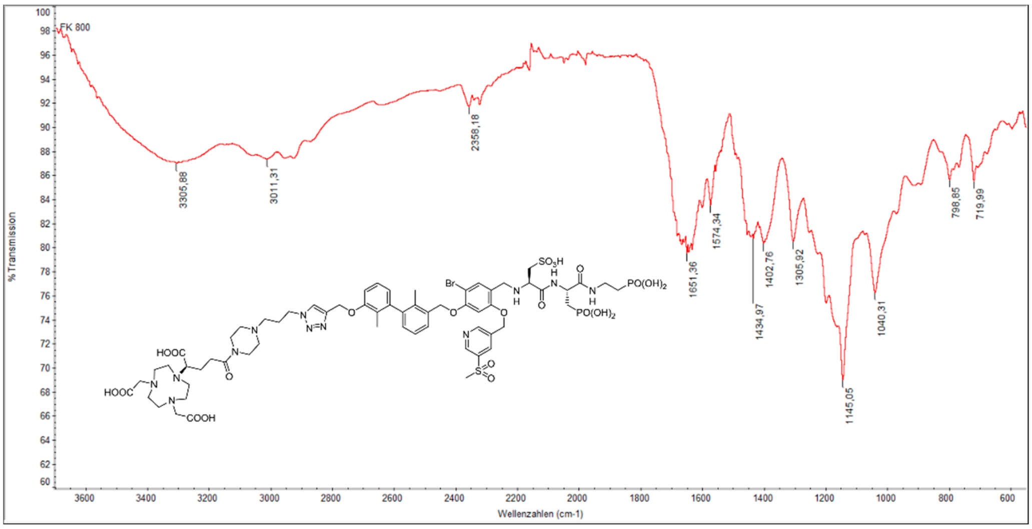

Figure S51: ATR-IR spectrum of compound **2**.

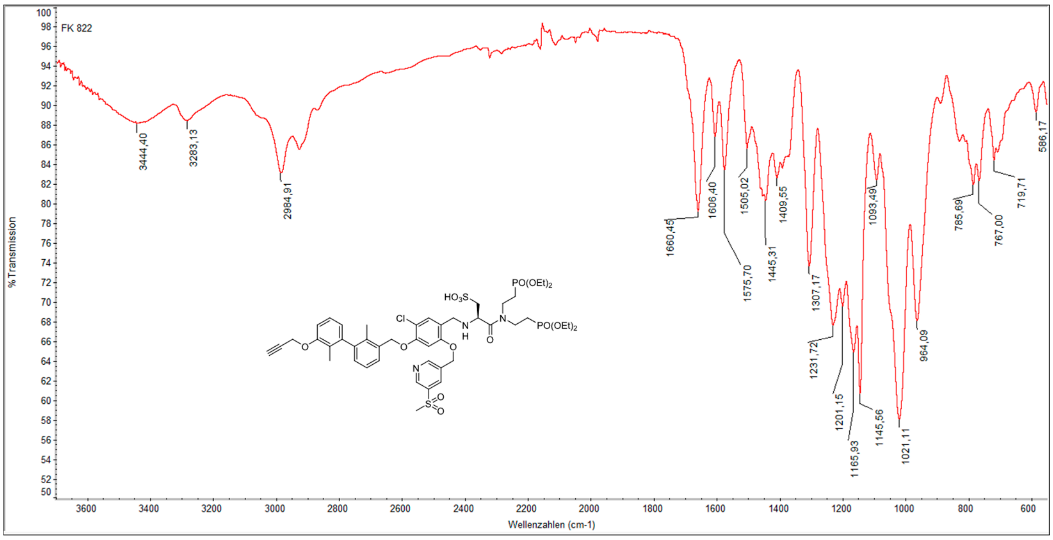

Figure S52: ATR-IR spectrum of compound **8**.

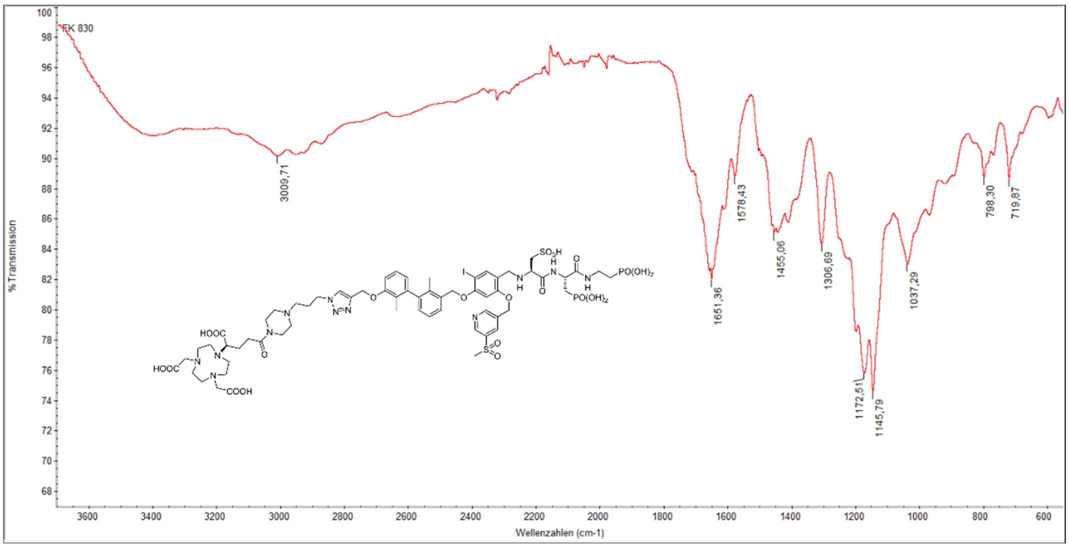

Figure S53: ATR-IR spectrum of compound **3**.

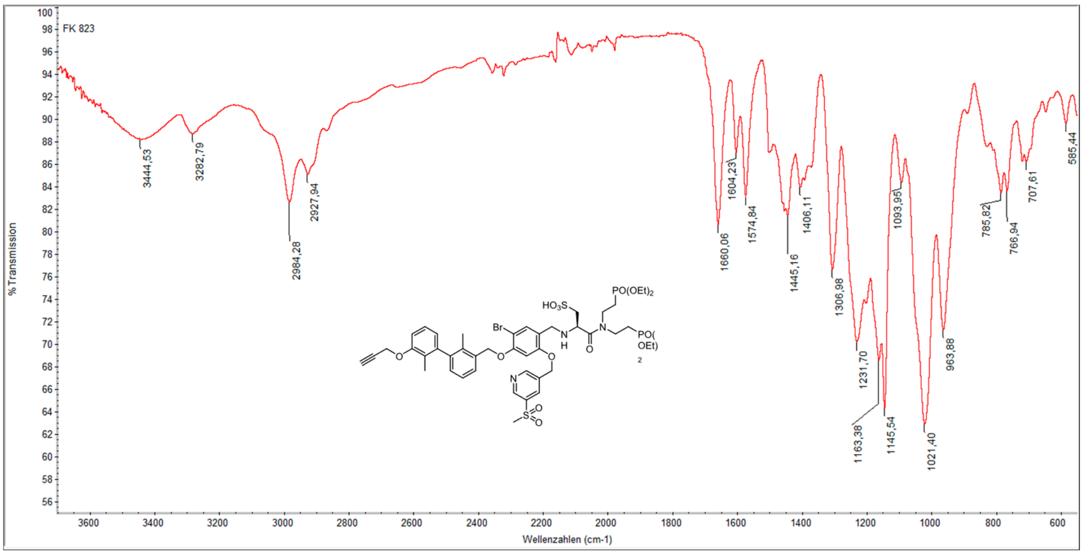

Figure S54: ATR-IR spectrum of compound **9**.

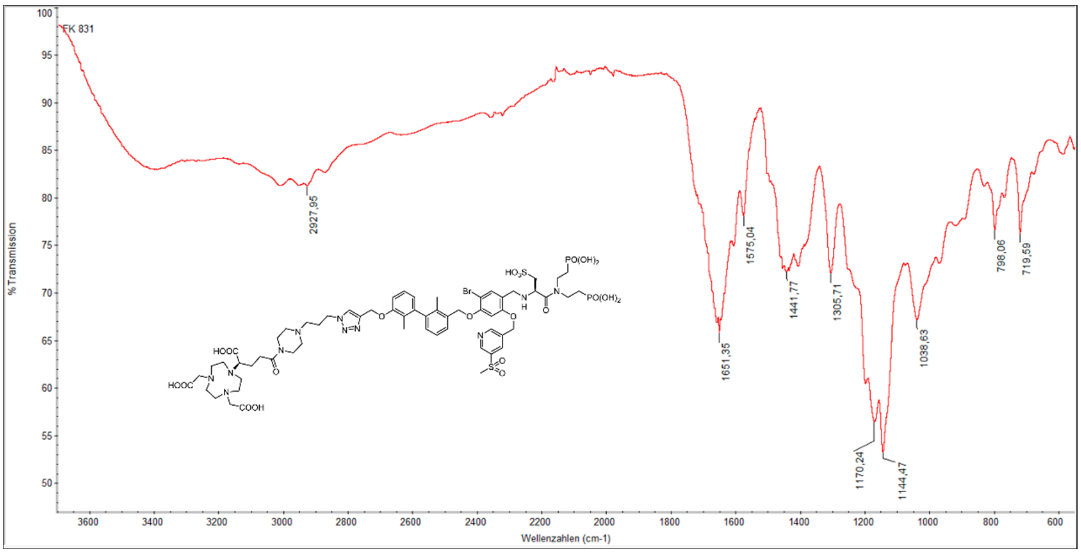

Figure S55: ATR-IR spectrum of compound **4**.

4. HPLC-chromatograms of HPLC purified compounds

Analytical reversed HPLC was conducted on an Agilent C18 column (Agilent Zorbax 300SB-C18, 100 mm × 4.6 mm) with acetonitrile/water (0.1% TFA each) as mobile phase. Preparative and semi-preparative reversed HPLC separations were performed on the Knauer Azura on Zorbax SB C-18 5 µm 80 Å, 9.4 x 250 mm as stationary phase with acetonitrile/water (0.1% TFA each) as mobile phase.

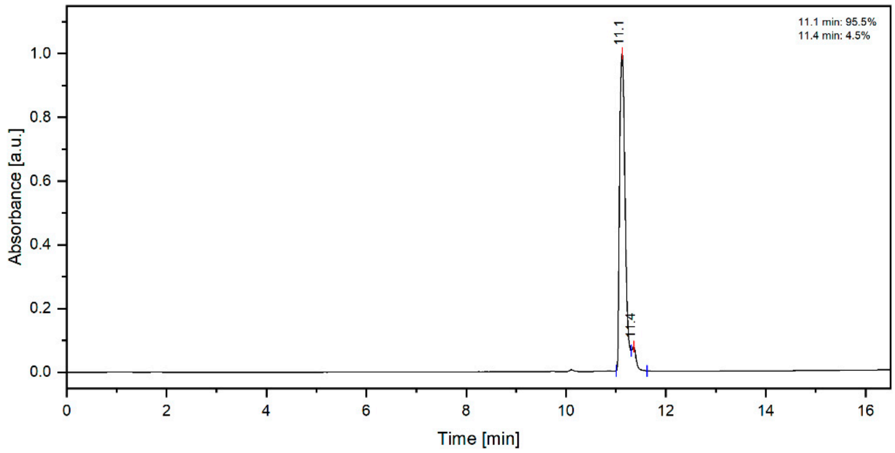

Figure S56: Analytical RP-HPLC chromatogram (System A) of compound **27**.

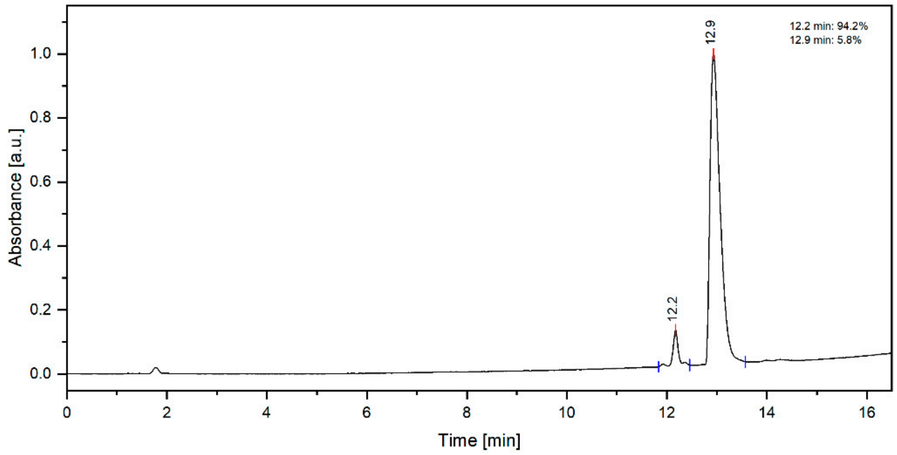

Figure S57: Analytical RP-HPLC chromatogram (System A) of compound **35**.

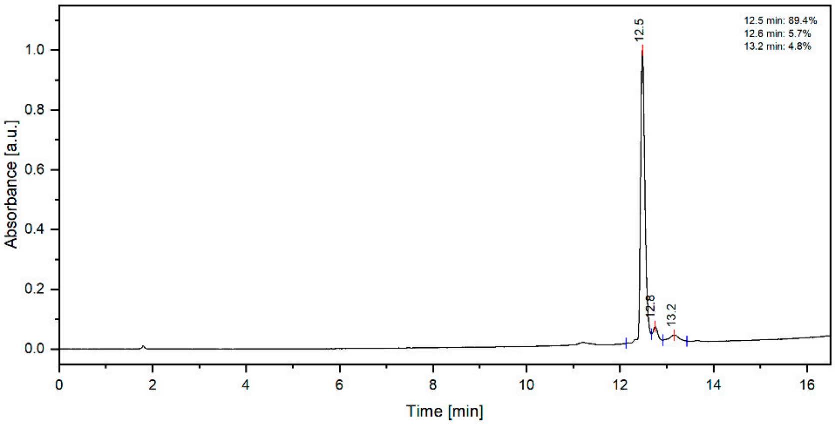

Figure S58: Analytical RP-HPLC chromatogram (System A) of compound **6**.

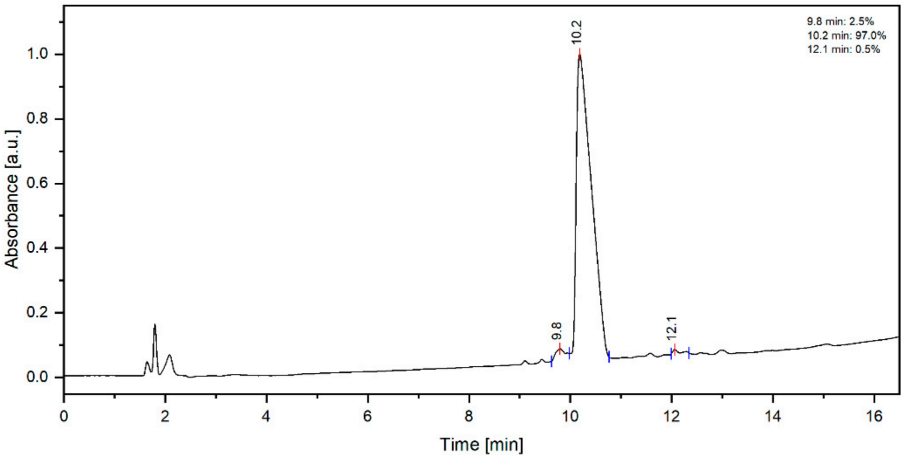

Figure S59: Analytical RP-HPLC chromatogram (System A) of compound **5.**

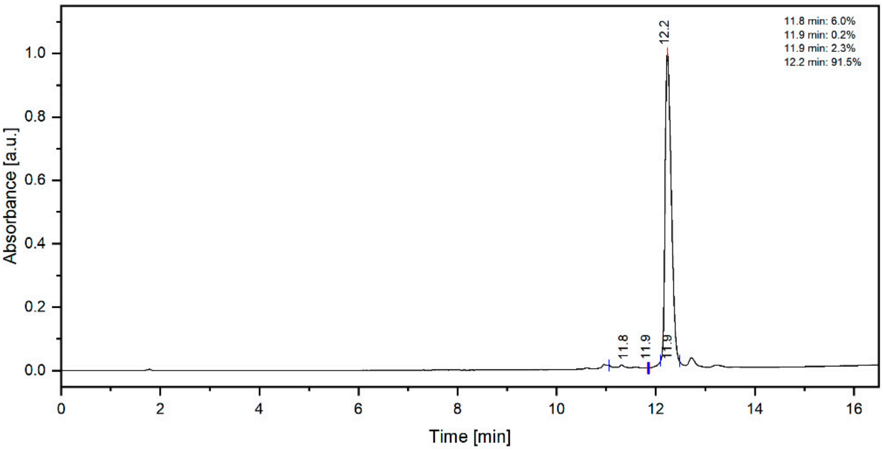

Figure S60: Analytical RP-HPLC chromatogram (System A) of compound **37**.

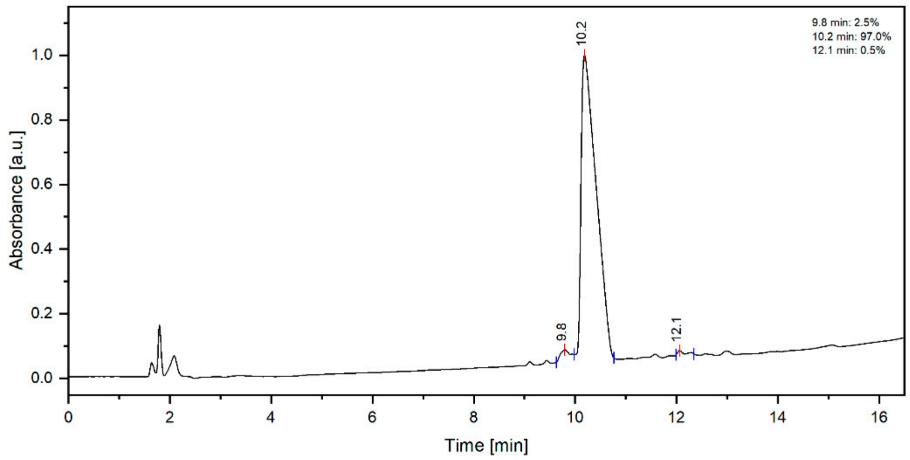

Figure S61: Analytical RP-HPLC chromatogram (System A) of compound **23**.

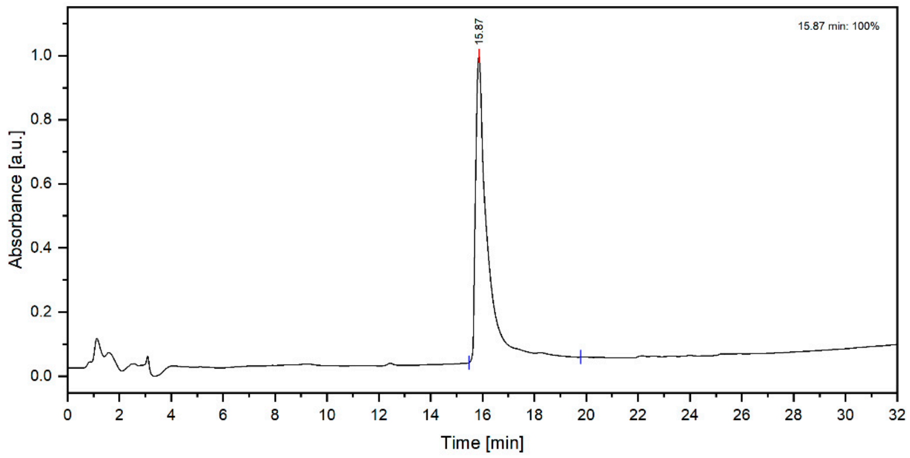

Figure S62: Analytical RP-HPLC chromatogram (System B) of compound **1**.

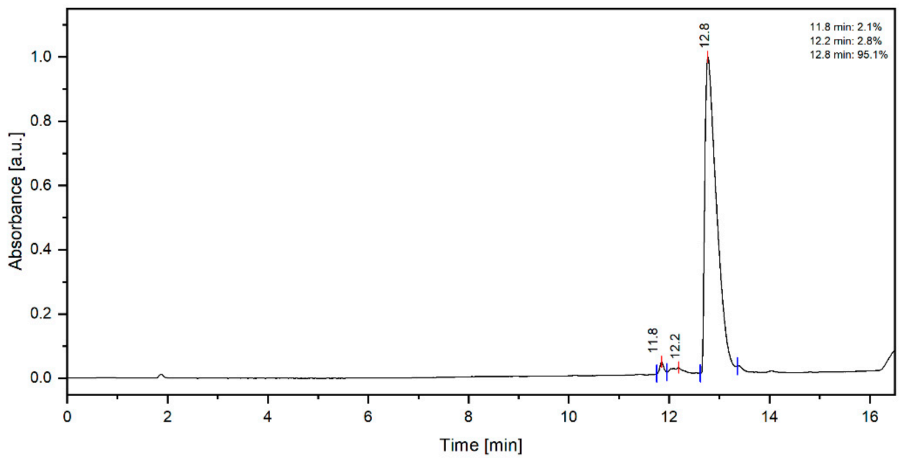

Figure S63: Analytical RP-HPLC chromatogram (System A) of compound **36**.

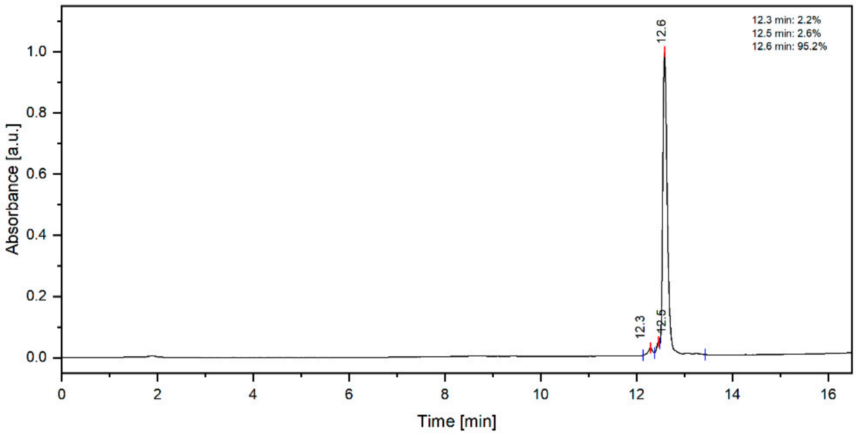

Figure S64: Analytical RP-HPLC chromatogram (System A) of compound **7**.

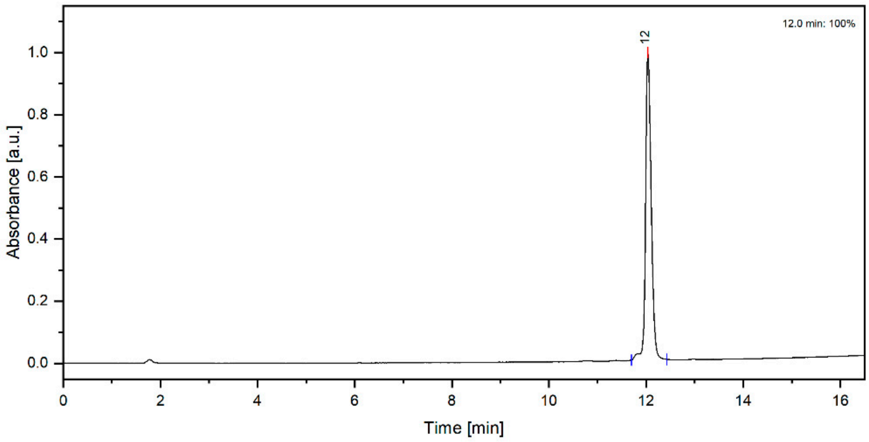

Figure S65: Analytical RP-HPLC chromatogram (System A) of compound **38**.

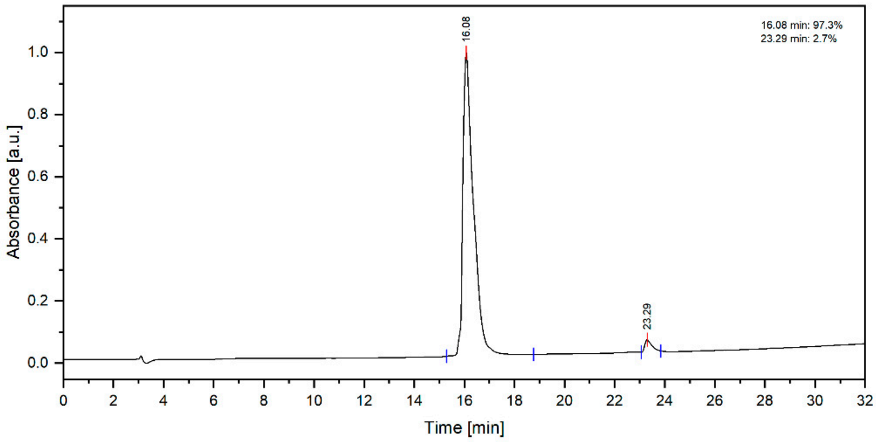

Figure S66: Analytical RP-HPLC chromatogram (System B) of compound **2**.

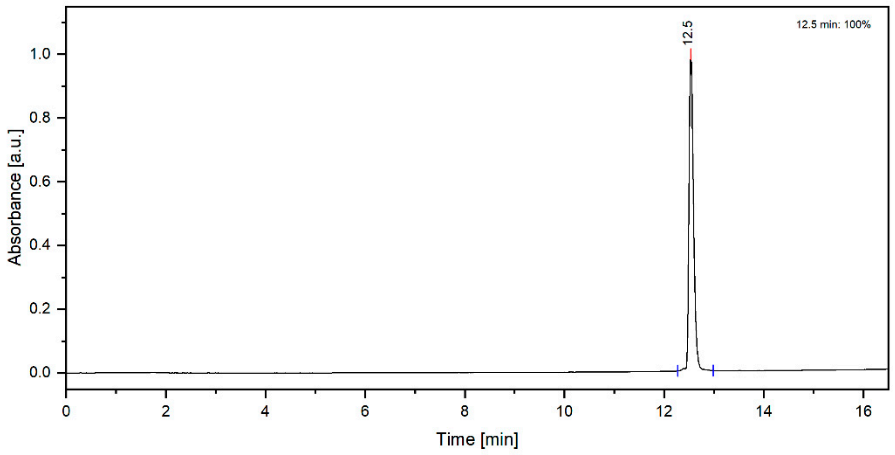

Figure S67: Analytical RP-HPLC chromatogram (System A) of compound **8**.

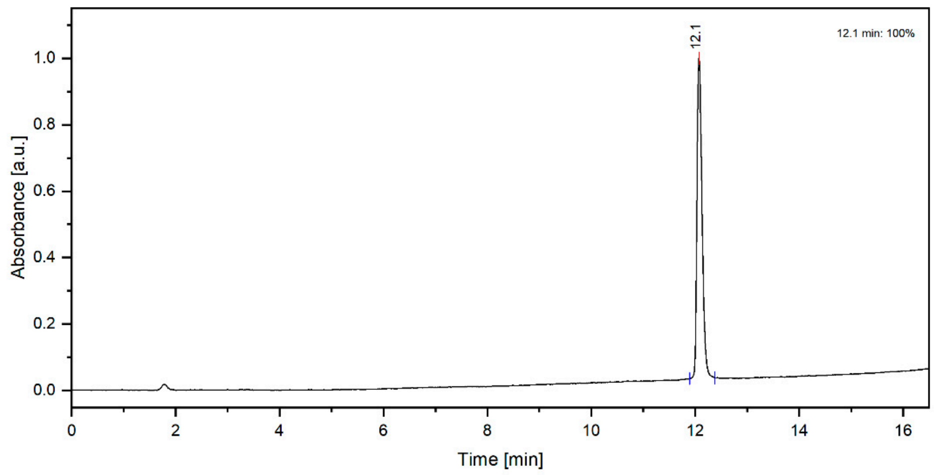

Figure S68: Analytical RP-HPLC chromatogram (System A) of compound **39**.

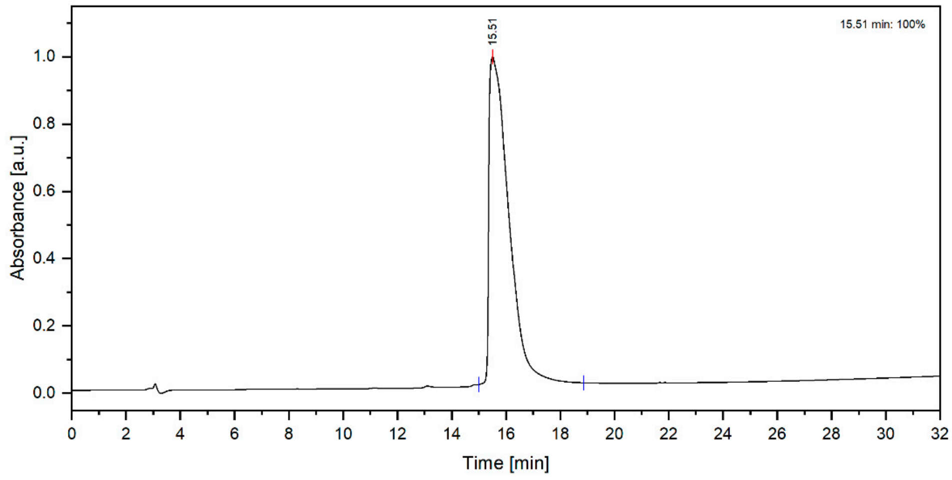

Figure S69: Analytical RP-HPLC chromatogram (System A) of compound **3**.

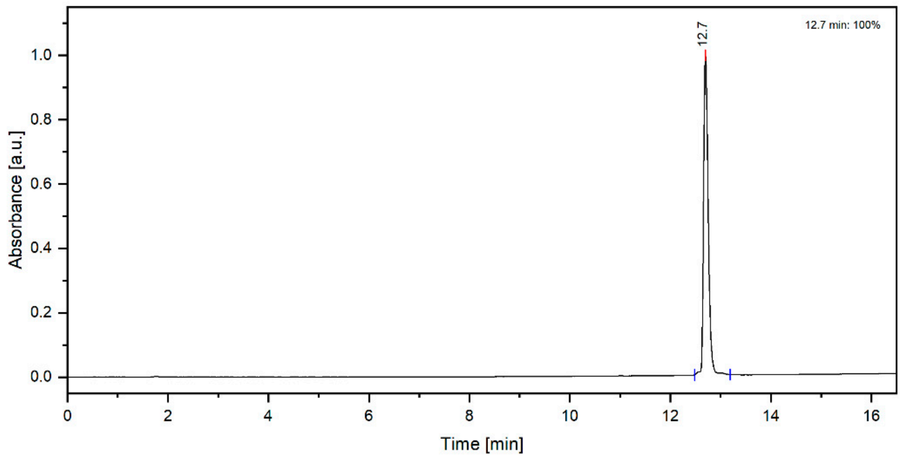

Figure S70: Analytical RP-HPLC chromatogram (System A) of compound **9**.

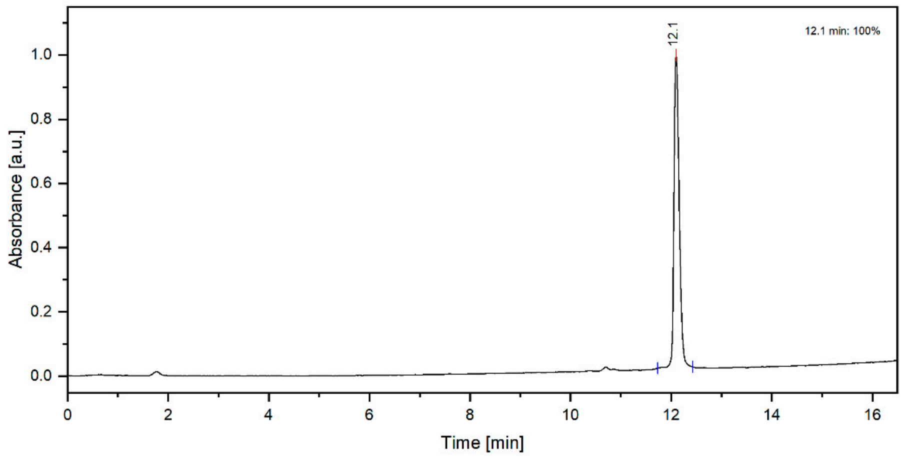

Figure S71: Analytical RP-HPLC chromatogram (System A) of compound **40**.

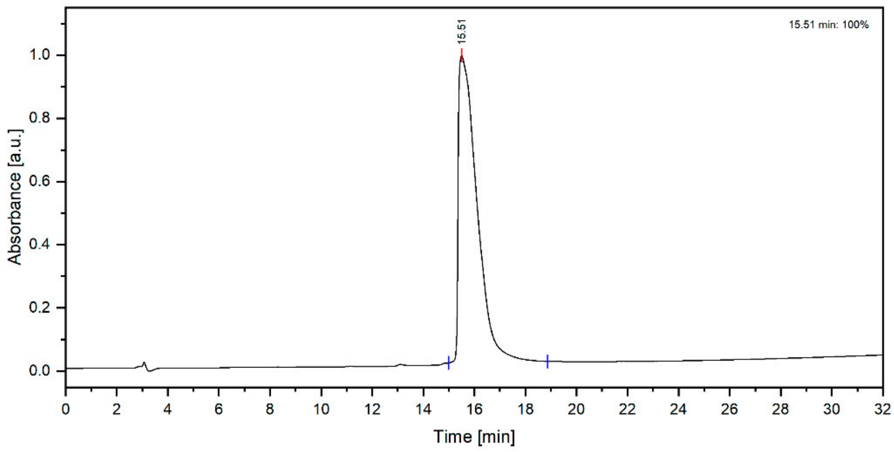

Figure S72: Analytical RP-HPLC chromatogram (System A) of compound **4**.

5. Mass spectra of literature unknown compounds

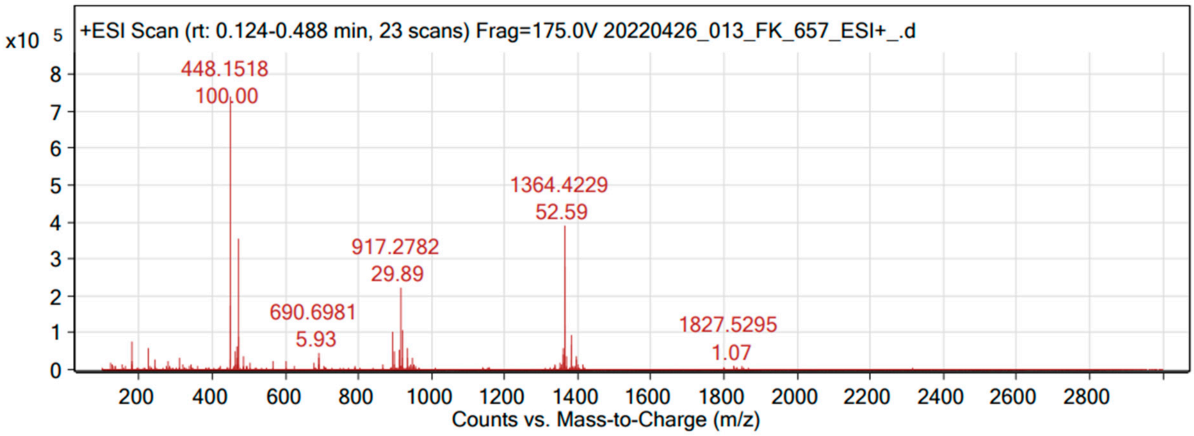

Figure S73: HR-MS-Spectrum (ESI+) of compound **25**.

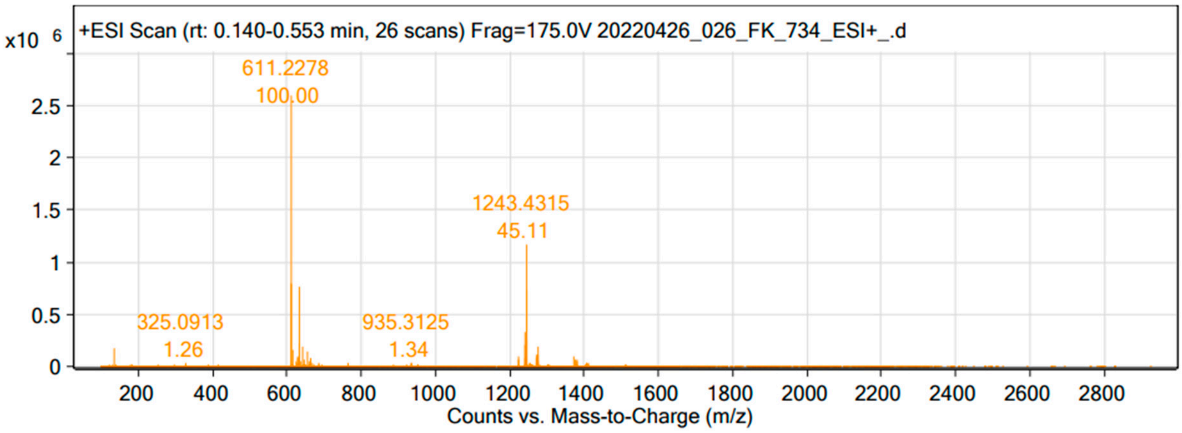

Figure S74: HR-MS-Spectrum (ESI+) of compound **27**.

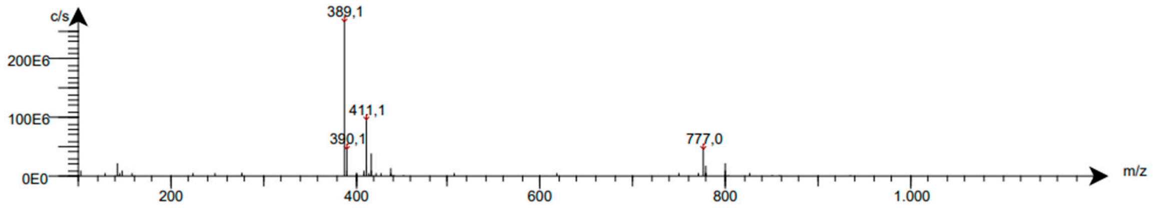

Figure S75: MS-Spectrum (ESI+) of compound **13**.

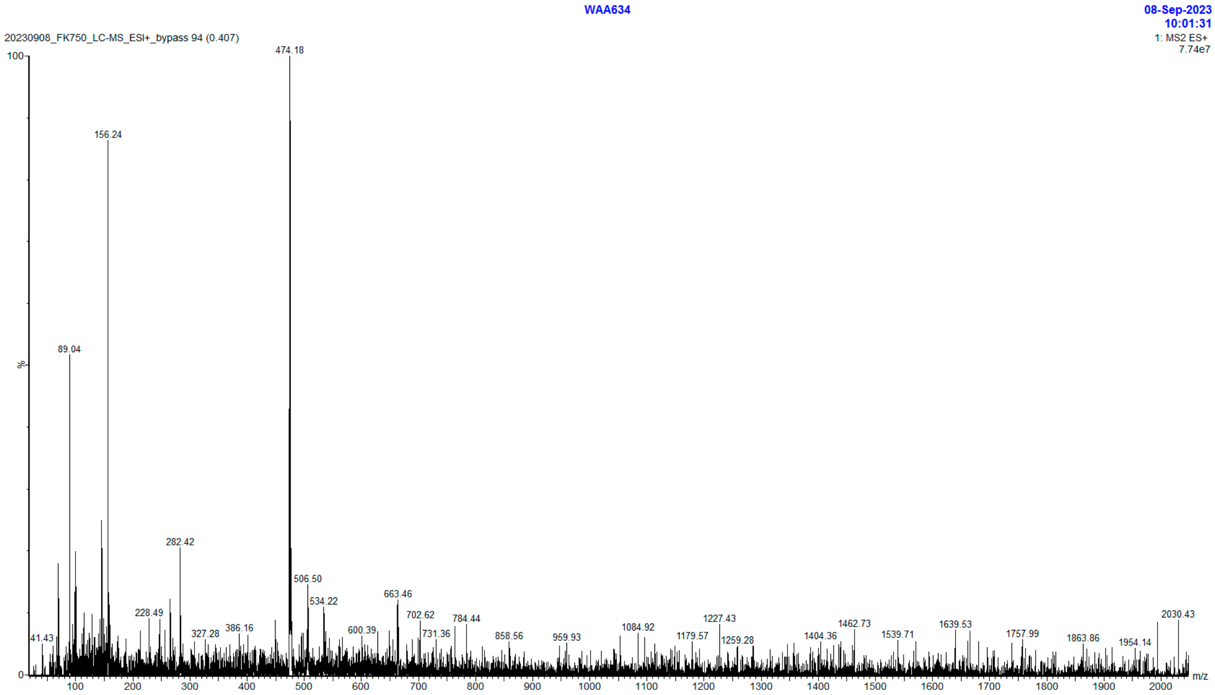

Figure S76: MS-Spectrum (ESI+) of compound **24**.

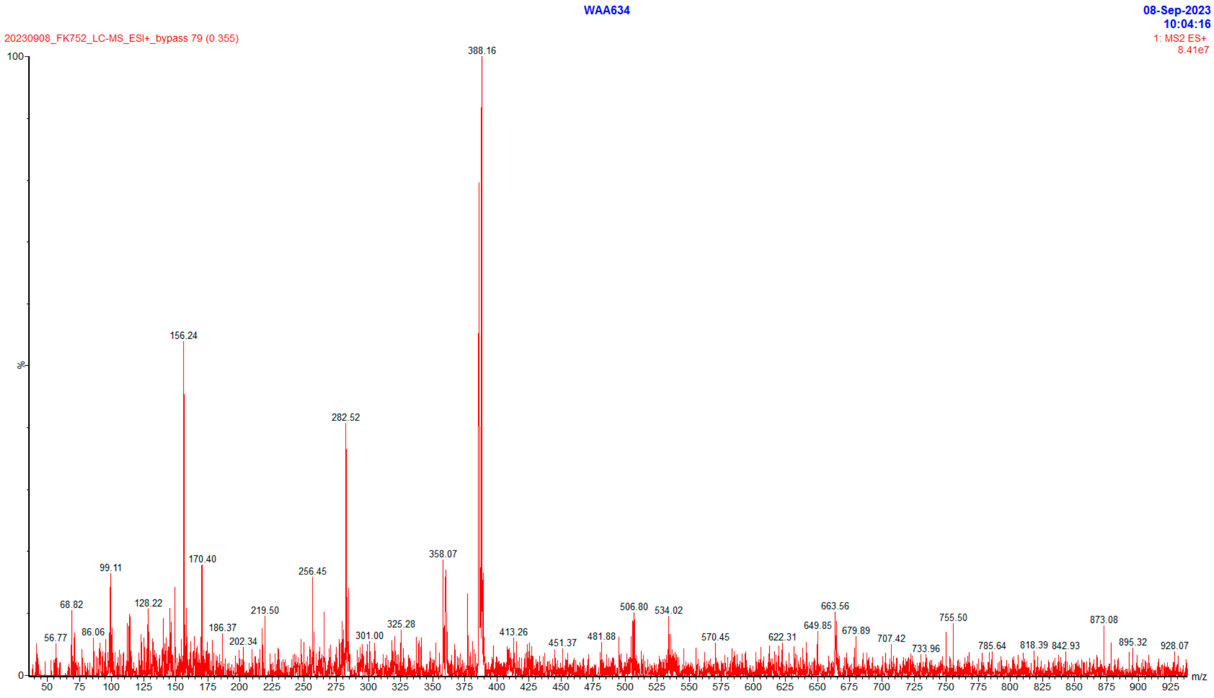

Figure S77: MS-Spectrum (ESI+) of compound **17**.

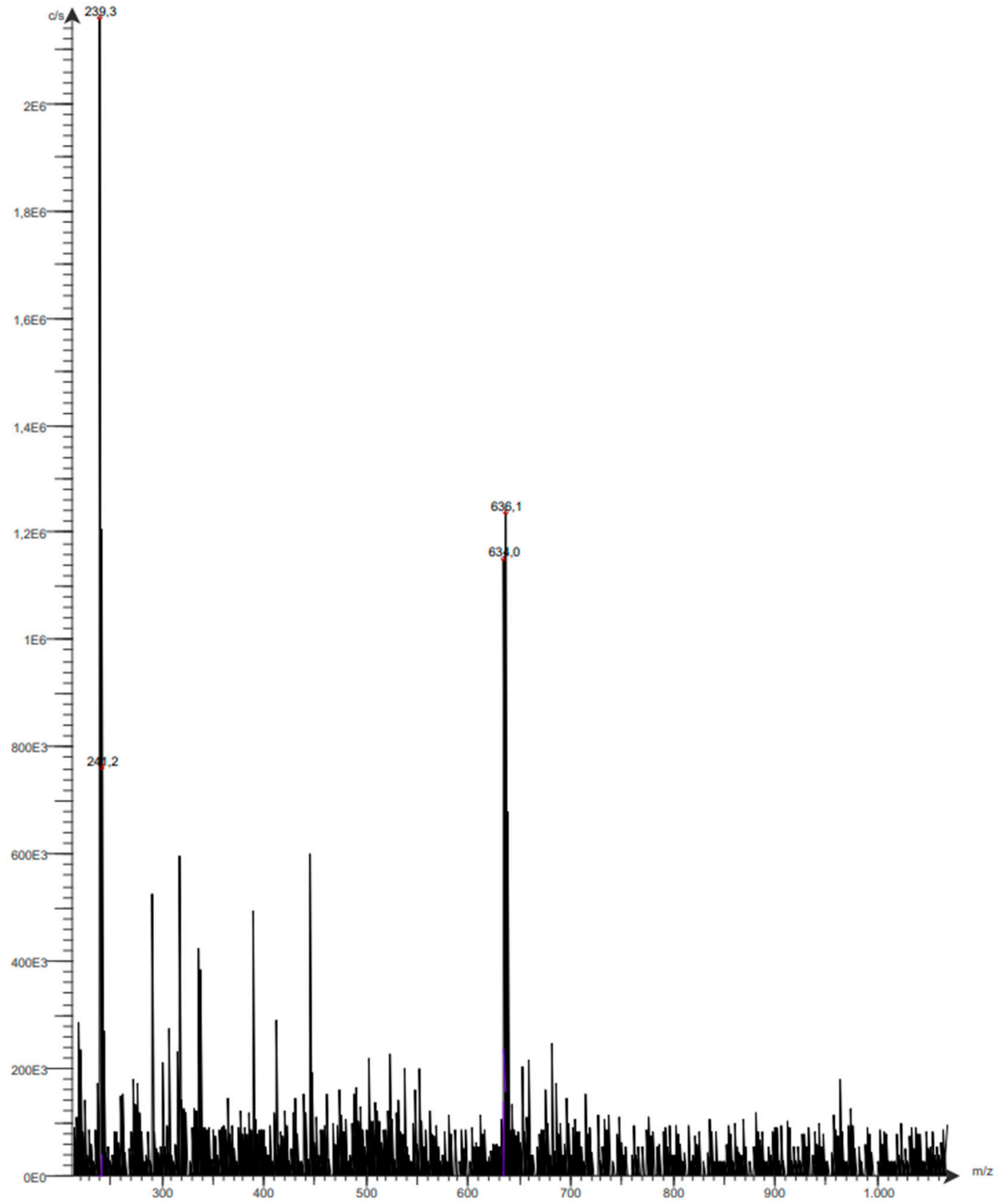

Figure S78: MS-Spectrum (ESI+) of compound **11.**

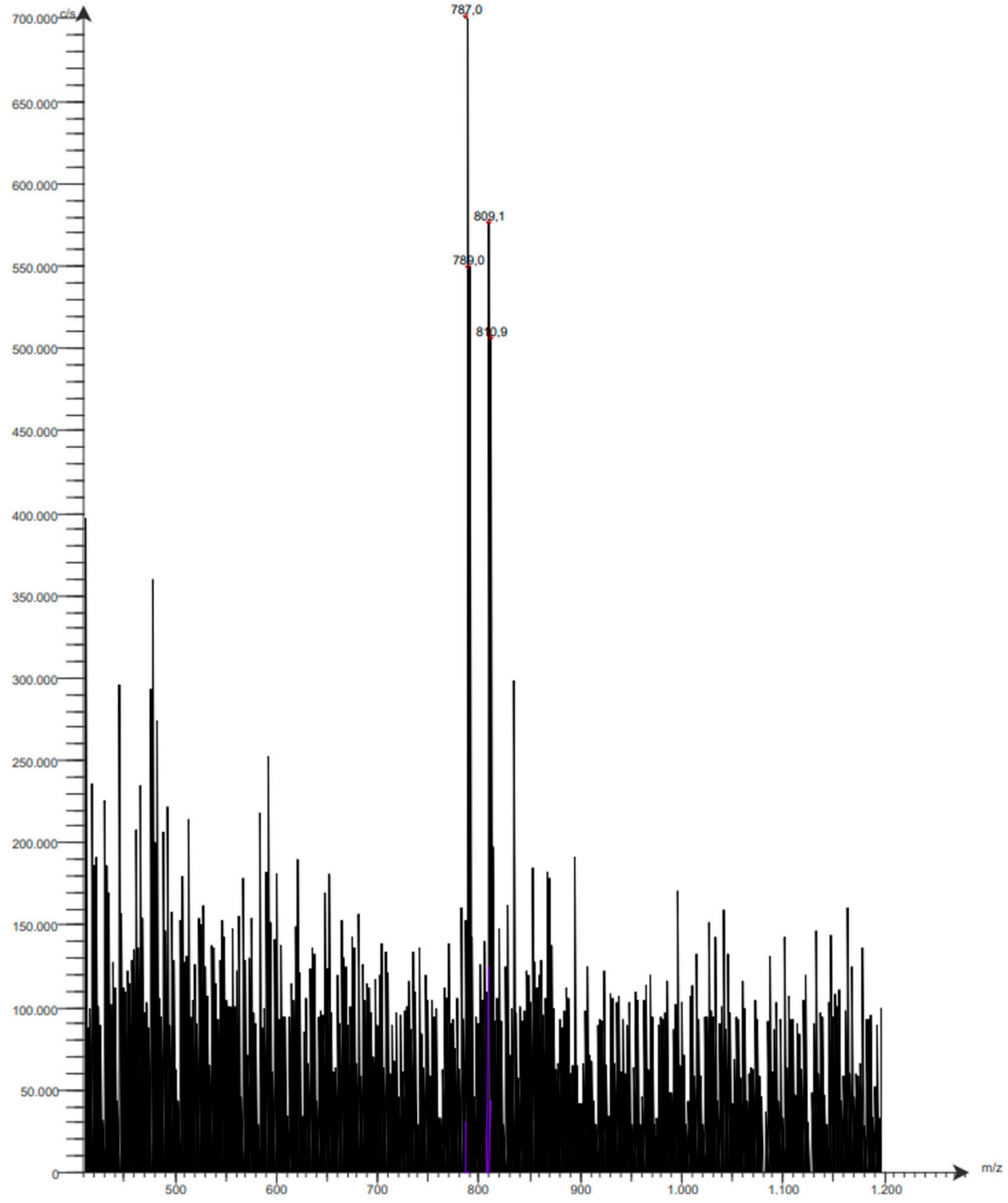

Figure S79: MS-Spectrum (ESI+) of compound **35.**

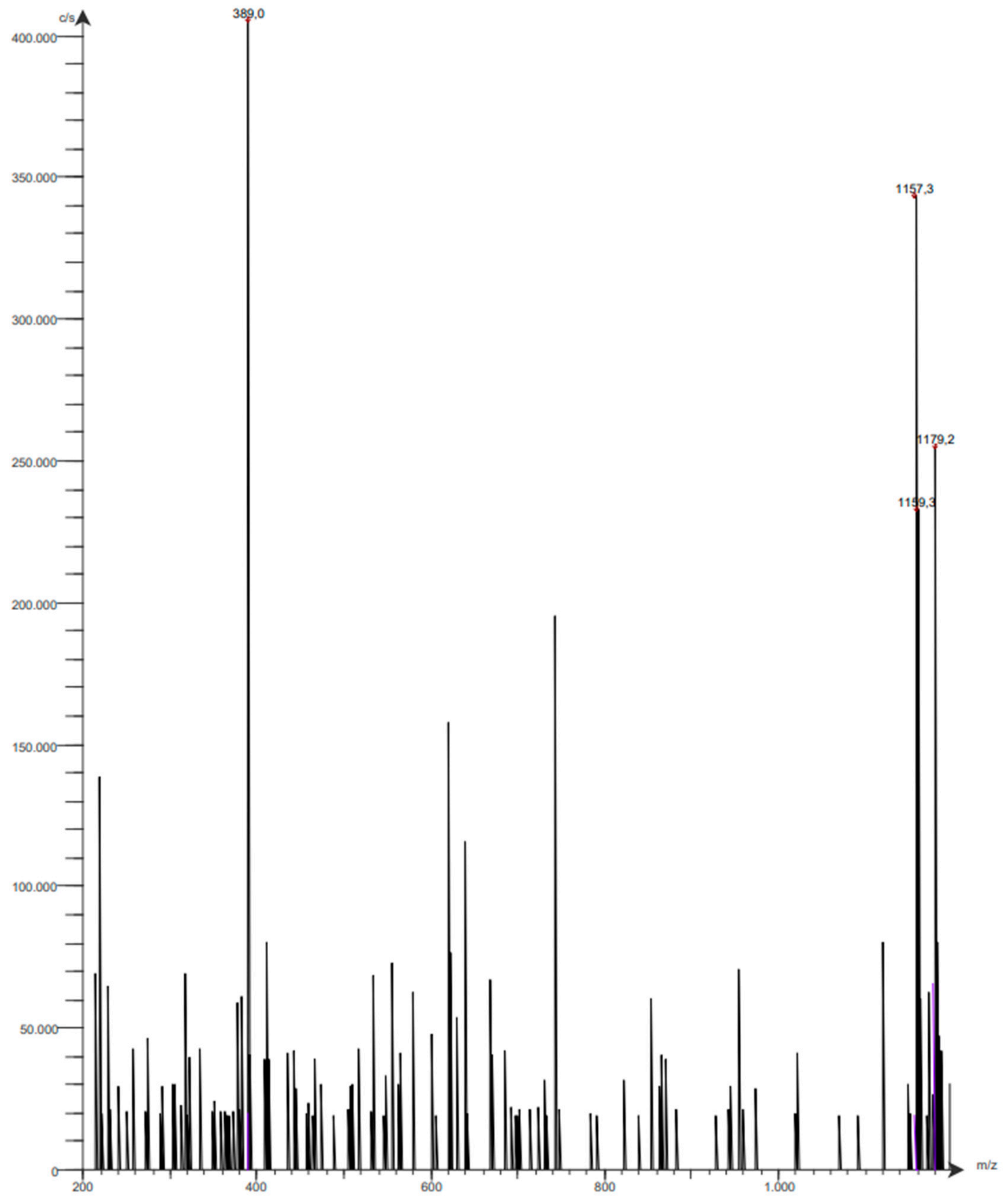

Figure S80: MS-Spectrum (ESI+) of compound **6.**

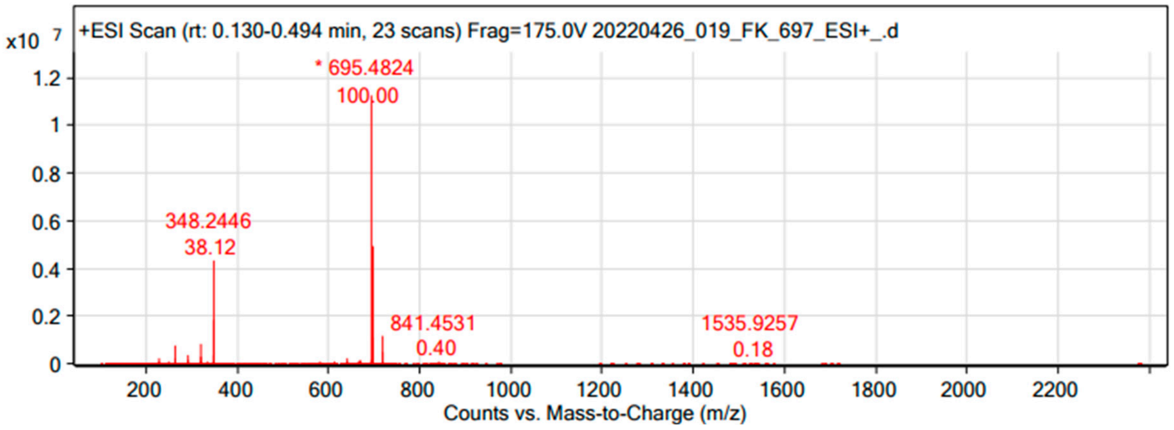

Figure S81: HR-MS-Spectrum (ESI+) of compound **5**.

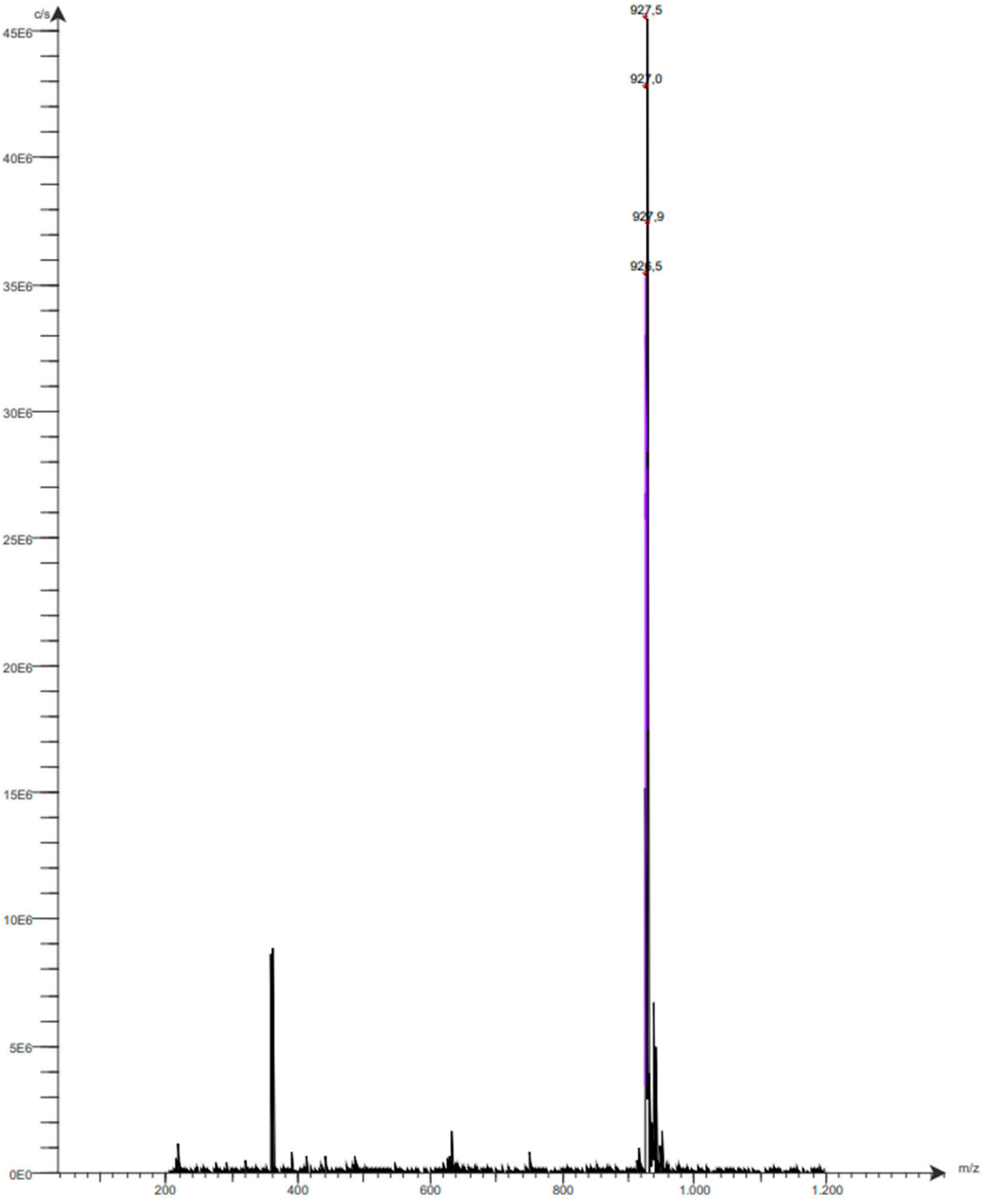

Figure S82: MS-Spectrum (ESI+) of compound **37**.

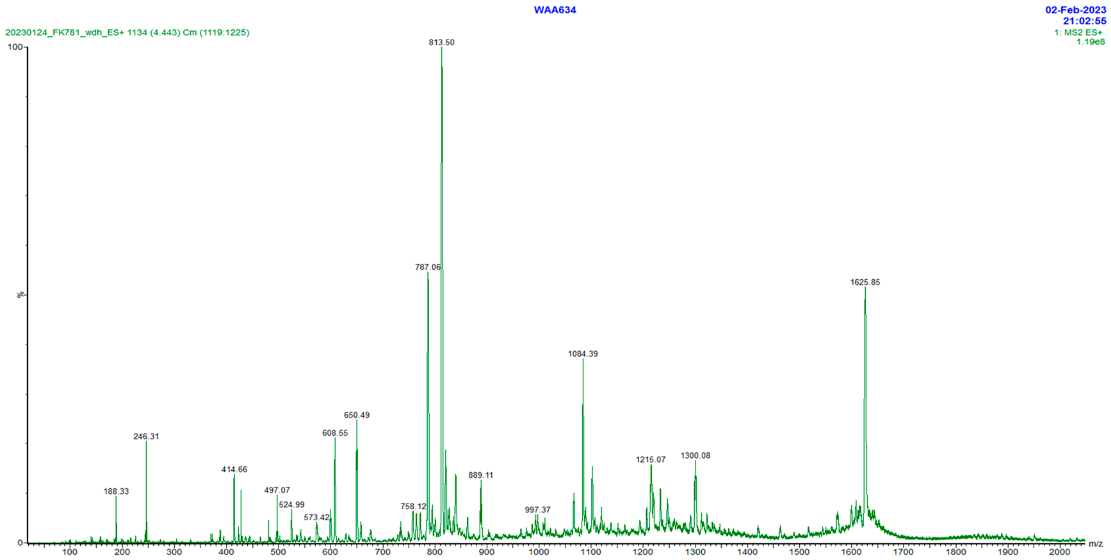

Figure S83: MS-Spectrum (ESI+) of compound **1**.

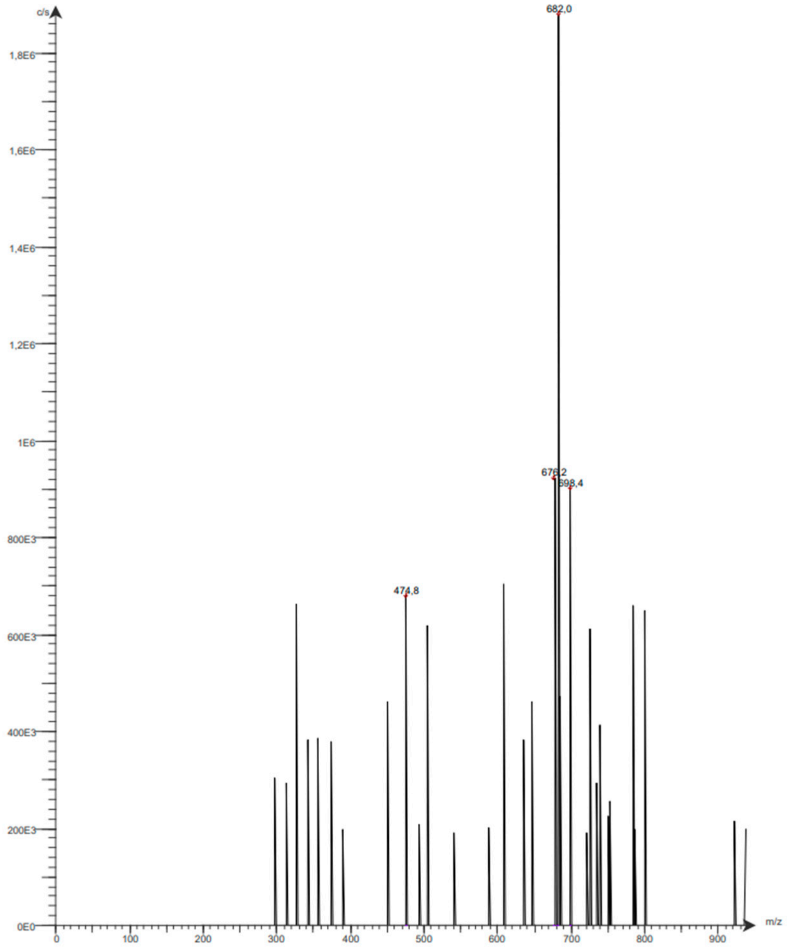

Figure S84: HR-MS-Spectrum (ESI+) of compound **12**.

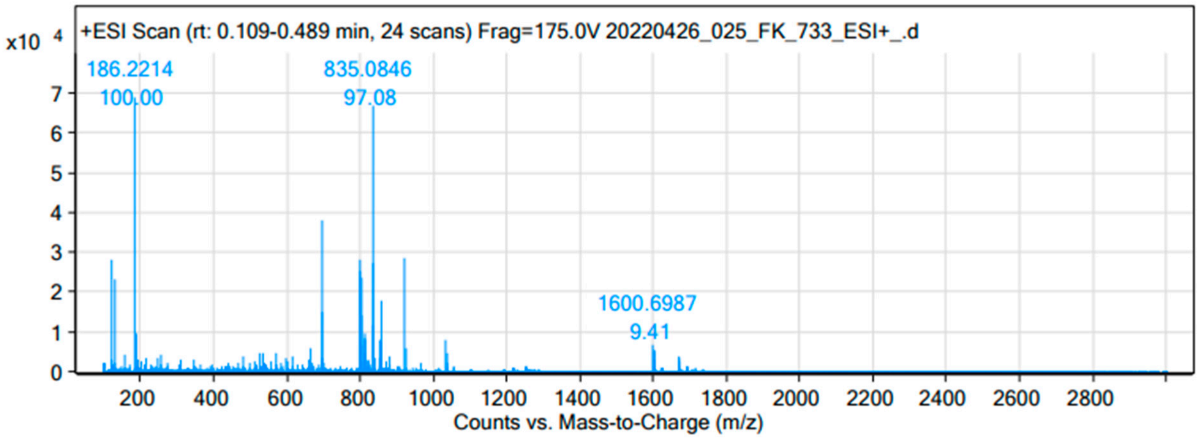

Figure S85: HR-MS-Spectrum (ESI+) of compound **36.**

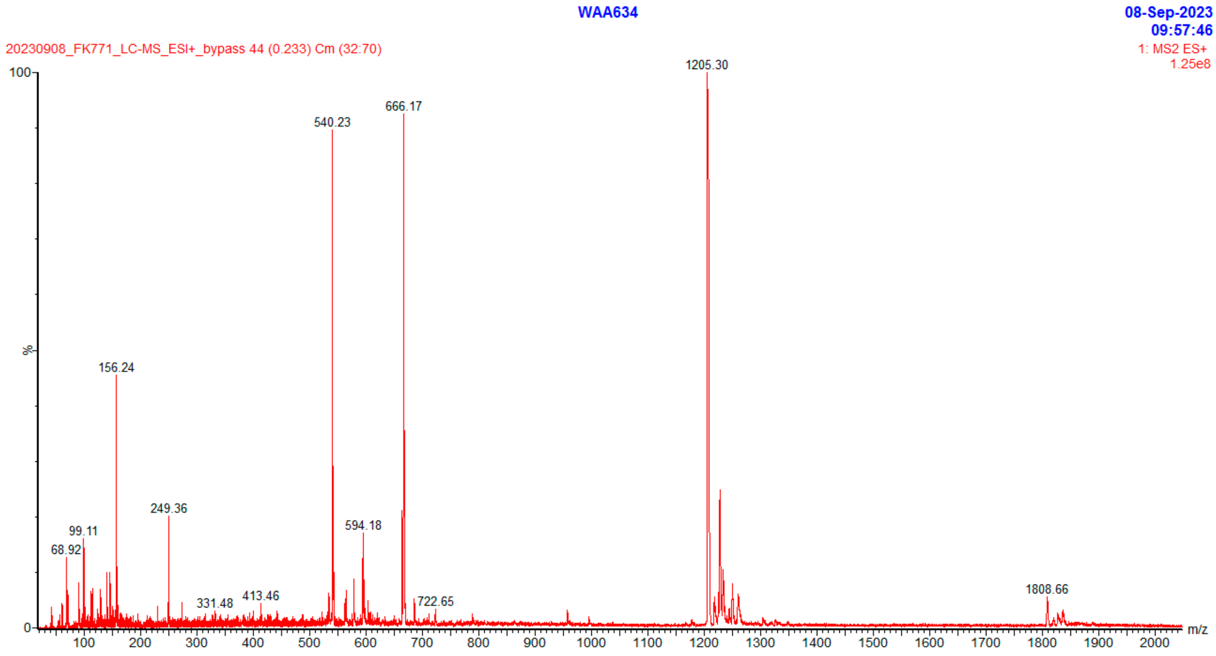

Figure S86: MS-Spectrum (ESI+) of compound **7**.

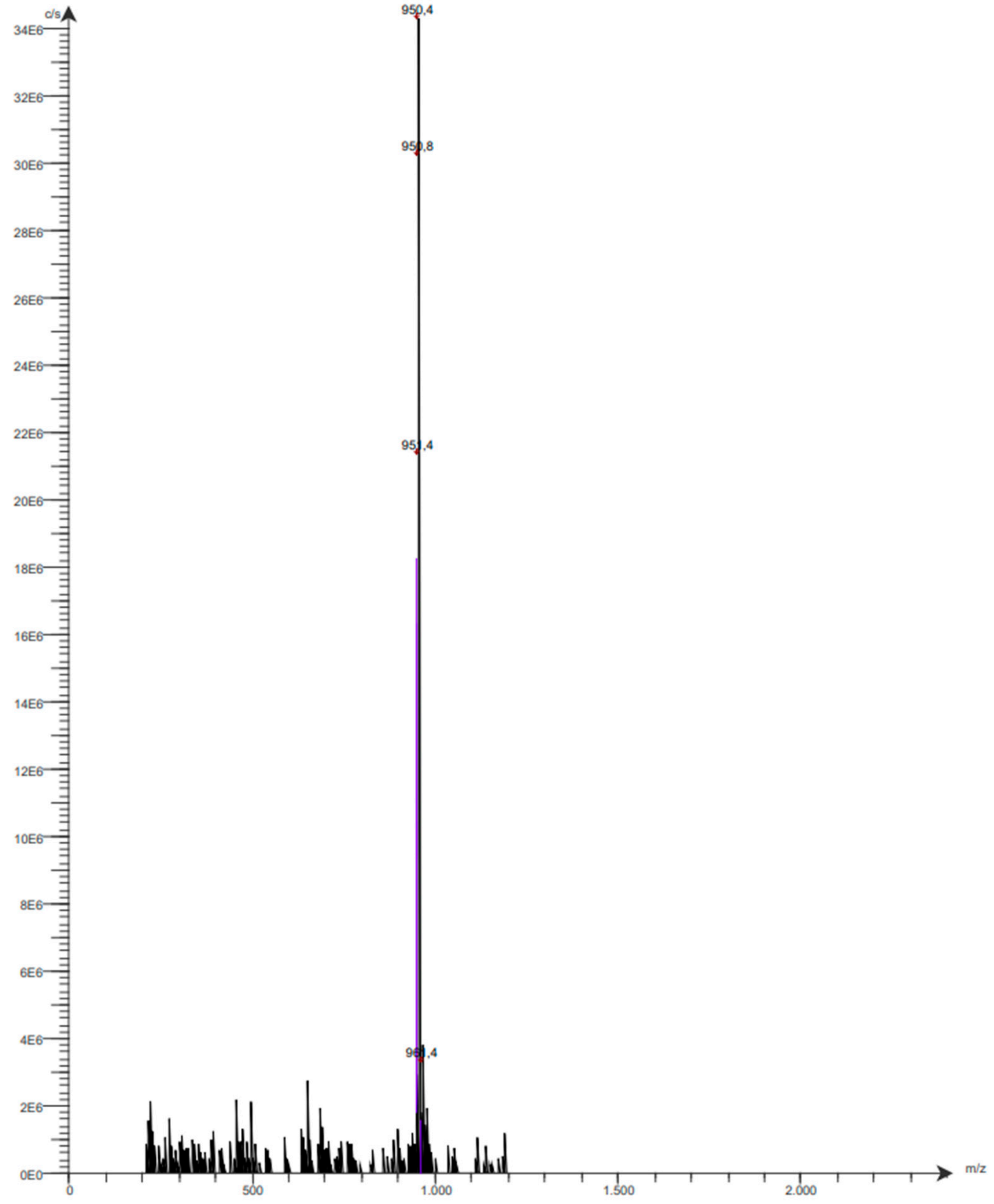

Figure S87: MS-Spectrum (ESI+) of compound **38**.

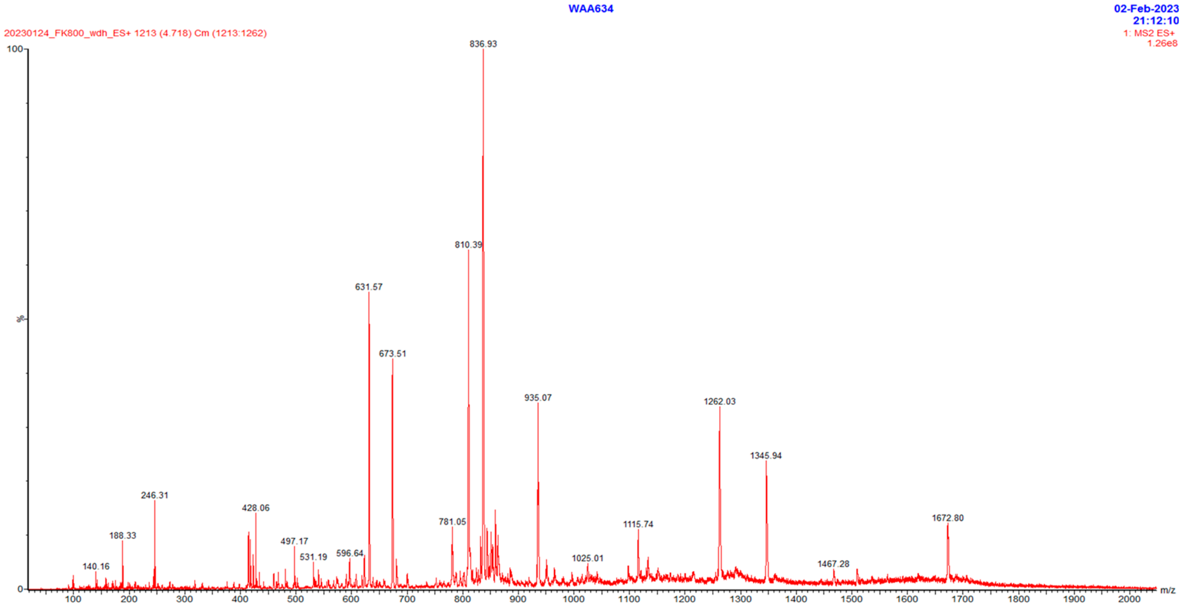

Figure S88: MS-Spectrum (ESI+) of compound **2**.

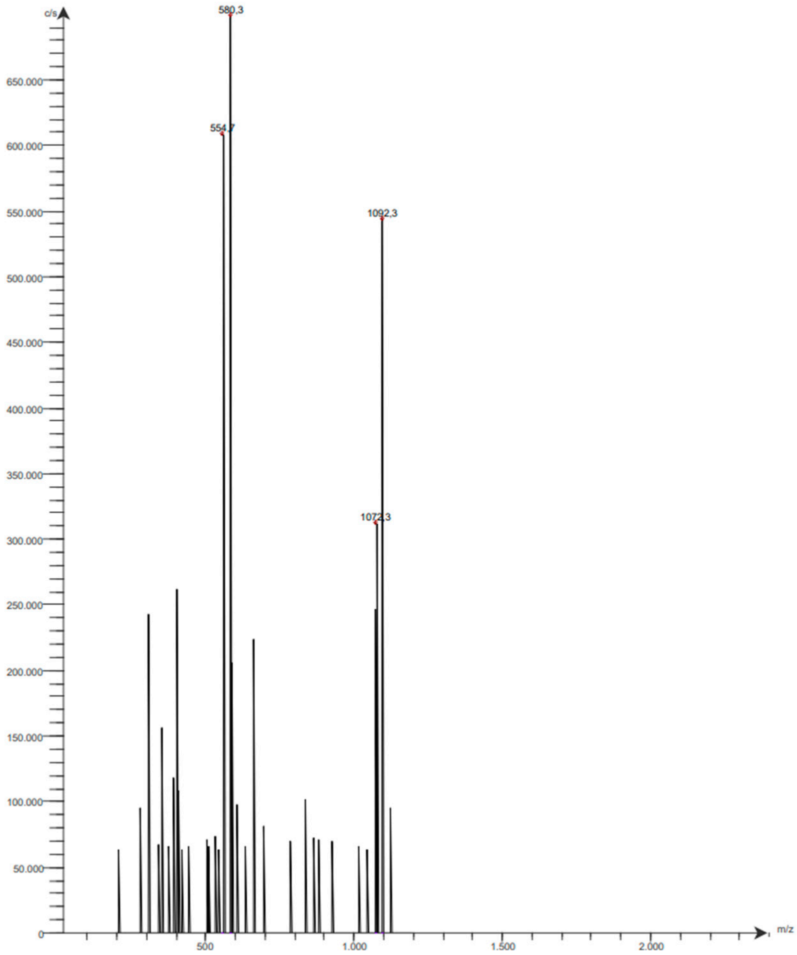

Figure S89: MS-Spectrum (ESI+) of compound **8**.

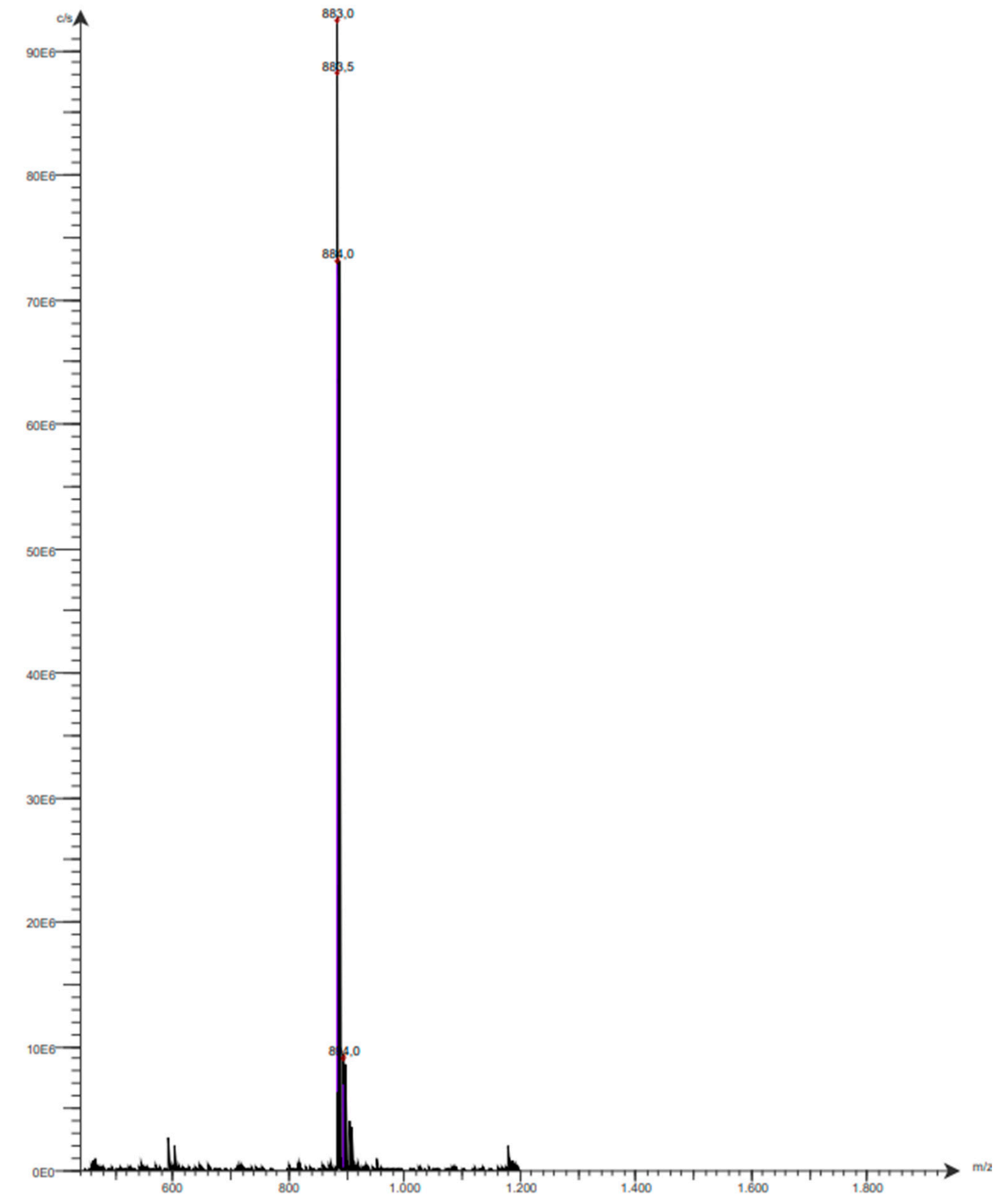

Figure S90: MS-Spectrum (ESI+) of compound **39**.

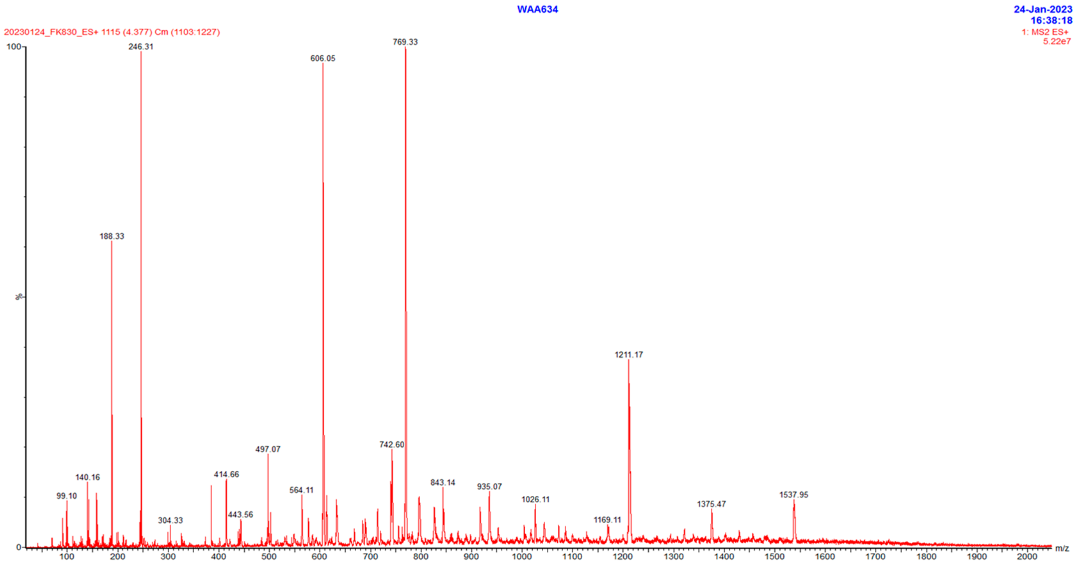

Figure S91: MS-Spectrum (ESI+) of compound **3**.

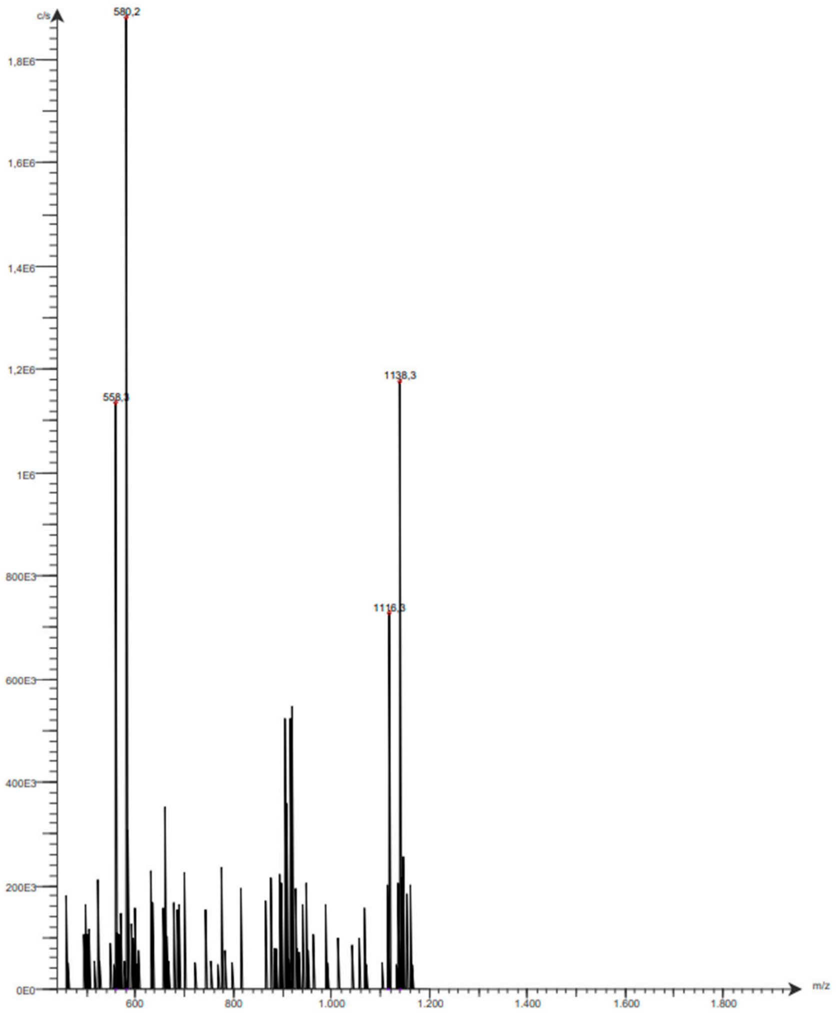

Figure S92: MS-Spectrum (ESI+) of compound **9**.

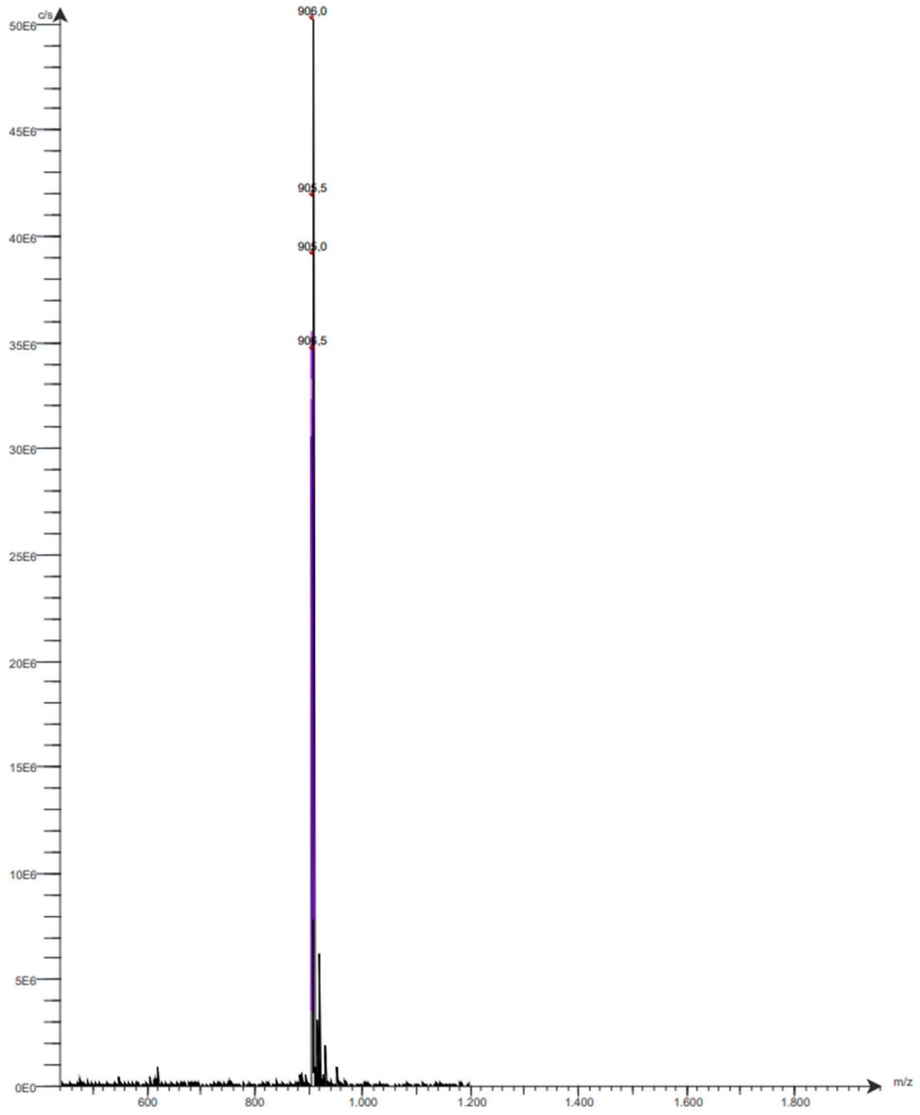

Figure S93: MS-Spectrum (ESI+) of compound **40**.

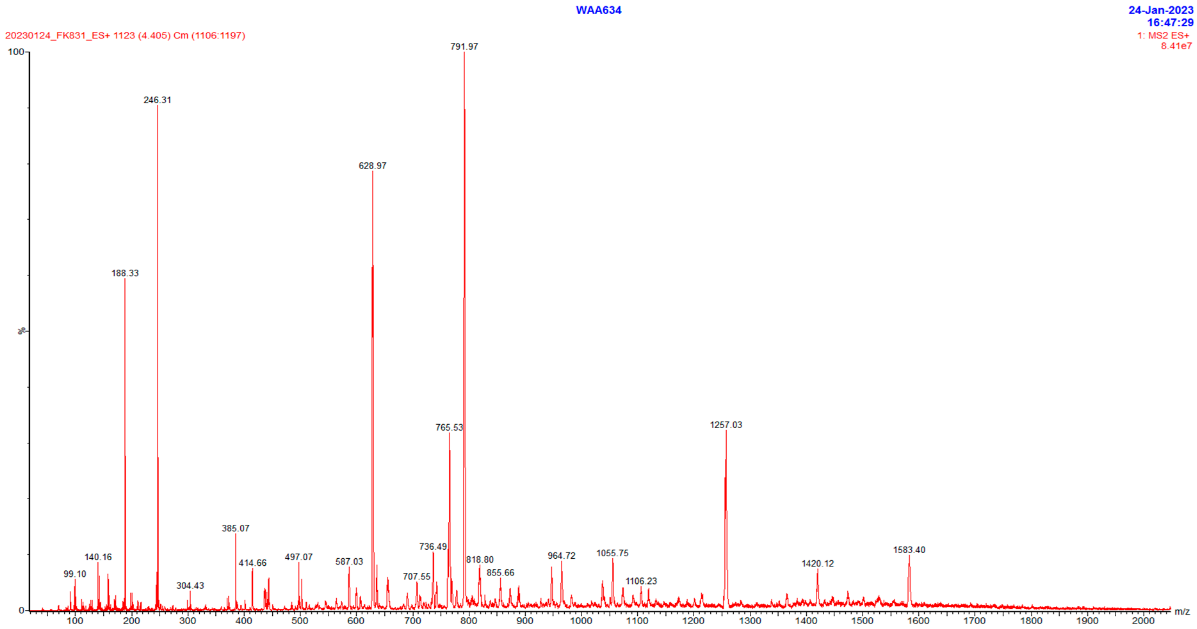

Figure S94: MS-Spectrum (ESI+) of compound **4.**

6. Biological data

Table S1: *In vivo* standardized uptake values (SUV_{max} ± standard deviation) of ^{64}Cu-labeled compounds [^{64}Cu]Cu-**1**–**4** at 1.5 (1–2), 4.5 (4–5) and 24.5 (24–25) hours post injection (p.i.).

Compound (# animals)	Volume of interest	1.5 h (1–2 h) p.i		4.5 h (4–5 h) p.i		24.5 h (24–25 h) p.i	
		SUV_{max}	SD	SUV_{max}	SD	SUV_{max}	SD
[^{64}Cu]Cu-**1** (n=2)	PD-L1 positive tumor	**1.60**	**1.17**	**1.28**	**0.39**	**2.93**	**1.04**
	PD-L1 negative tumor	**0.63**	**0.04**	**1.49**	**0.55**	**2.77**	**0.20**
	Liver	7.96	0.48	9.15	0.16	8.71	0.16
	Kidney	1.90	0.23	0.94	0.14	1.39	0.25
	Joints	2.24	0.36	1.57	0.65	1.93	0.00
[^{64}Cu]Cu-**1** (n=2) +0.5/1 µmol **1**	PD-L1 positive tumor	**0.91**	**0.20**	**0.55**	**0.28**	**0.59**	**0.36**
	PD-L1 negative tumor	**0.70**	**0.02**	**0.36**	**0.29**	**0.27**	**0.15**
	Liver	4.90	0.13	4.83	0.07	4.34	0.15
	Kidney	11.73	14.24	0.71	0.11	0.60	0.23
	Joints	7.27	0.21	9.34	2.06	8.62	0.46
[^{64}Cu]Cu-**2** (n=2)	PD-L1 positive tumor	**1.57**	**0.58**	**0.50**	**0.06**	**0.73**	**0.28**
	PD-L1 negative tumor	**1.30**	**0.51**	**0.56**	**0.08**	**0.64**	**0.25**
	Liver	4.39	0.11	1.11	0.08	1.41	0.33
	Kidney	7.16	2.09	0.83	0.09	0.42	0.11
	Joints	2.59	0.08	7.95	3.82	8.39	6.10
[^{64}Cu]Cu-**2** (n=2) +0.5 µmol **2**	PD-L1 positive tumor	**1.45**	**0.27**	**1.56**	**1.12**	**4.22**	**0.60**
	PD-L1 negative tumor	**1.00**	**0.07**	**1.68**	**0.37**	**3.20**	**0.52**
	Liver	1.83	0.43	6.44	0.93	5.45	0.56
	Kidney	9.50	11.48	1.59	0.04	1.73	0.18
	Joints	6.92	0.22	2.42	0.37	2.63	0.73
[^{64}Cu]Cu-**3** (n=2)	PD-L1 positive tumor	**1.20**	**0.42**	**2.08**	**1.02**	**3.01**	**0.46**
	PD-L1 negative tumor	**1.00**	**0.17**	**1.32**	**0.73**	**3.14**	**1.21**
	Liver	5.84	0.30	6.81	0.02	5.03	0.42
	Kidney	24.92	0.61	0.86	0.59	1.08	0.41
	Joints	1.63	0.52	1.21	0.46	1.31	0.51
[^{64}Cu]Cu-**3** (n=2) +0.5 µmol **3**	PD-L1 positive tumor	**1.91**	**1.29**	**0.63**	**0.11**	**1.46**	**0.33**
	PD-L1 negative tumor	**0.79**	**0.16**	**0.40**	**0.27**	**0.73**	**0.04**
	Liver	2.73	0.08	2.85	0.08	2.86	0.26
	Kidney	15.52	18.84	0.62	0.11	0.51	0.13
	Joints	4.00	0.15	4.81	0.39	4.55	1.07
[^{64}Cu]Cu-**4** (n=2)	PD-L1 positive tumor	**1.80**	**0.00**	**1.09**	**1.01**	**0.89**	**0.14**
	PD-L1 negative tumor	**0.77**	**0.28**	**0.80**	**0.33**	**1.63**	**0.29**
	Liver	5.24	0.76	3.60	2.47	2.18	0.20
	Kidney	1.37	0.04	0.38	0.18	0.81	0.40
	Joints	1.91	0.28	3.64	2.73	4.29	2.35
[^{64}Cu]Cu-**4** (n=2) +0.5 µmol **4**	PD-L1 positive tumor	**1.23**	**0.45**	**0.88**	**0.11**	**3.55**	**0.83**
	PD-L1 negative tumor	**0.64**	**0.20**	**1.71**	**0.92**	**3.18**	**0.22**
	Liver	2.15	0.03	3.67	1.51	3.74	0.42
	Kidney	1.89	0.19	0.43	0.15	0.67	0.30
	Joints	3.36	0.33	2.29	1.92	1.07	0.15
